# Revision of the Afrotropical Phaeogenini (Ichneumonidae, Ichneumoninae), with description of a new genus and twelve new species

**DOI:** 10.3897/zookeys.354.5968

**Published:** 2013-11-21

**Authors:** Pascal Rousse, Simon van Noort, E. Diller

**Affiliations:** 1 Natural History Department, Iziko South African Museum, PO Box 61, Cape Town, 8000, South Africa; 2 Stellenbosch University, Department of Botany and Zoology, Evolutionary Genomics Group, Private Bag X1, Stellenbosch 7602, South Africa; 3 Department of Zoology, University of Cape Town, Private Bag, Rondebosch, 7701, South Africa; 4 Zoologische Staatssammlung München, Münchhausenstrasse 21, D–81247 München, Germany

**Keywords:** Africa, Afrotropical region, Hymenoptera, identification key, Ichneumonidae, Phaeogenini, systematics, taxonomy

## Abstract

We revise the 10 genera and 23 species of the tribe Phaeogenini (Ichneumonidae: Ichneumoninae) known to occur in the Afrotropical region. We describe the following 13 new taxa: *Kibalus* Rousse, van Noort & Diller, **gen. n.**; *K. toro* Rousse, van Noort & Diller, **sp. n.**; *K. mubfs* Rousse & van Noort, **sp. n.**; *Arearia oxymoron* Rousse & van Noort, **sp. n.**; *Chauvinia nyanga* Rousse & van Noort, **sp. n.**; *Dicaelotus asantesana* Rousse & van Noort, **sp. n.**; *D. hoerikwaggoensis* Rousse & van Noort, **sp. n.**; *D. tablemountainensis* Rousse & van Noort, **sp. n.**; *Heterischnus mfongosi* Rousse & van Noort, **sp. n.**; *H. mkomazi* Rousse & van Noort, **sp. n.**; *Lusius flummox* Rousse & van Noort, **sp. n.**; *Tycherus amatola* Rousse & van Noort, **sp. n.**; and *T. nardousberg* Rousse & van Noort, **sp. n.** New distribution records: *Heterischnus africanus* (Heinrich, 1936) from South Africa, Tanzania and Uganda; *H. krausi* Schönitzer, 1999 from Rwanda; *Lusius tenuissimus* (Heinrich, 1938) from Democratic Republic of Congo, Malawi, Nigeria, South Africa, and Zimbabwe. A doubtful record of *Aethecerus foveolatus* Gregor, 1940 from Sao Tome is additionaly reported in the appendix. We provide illustrated diagnoses and identification notes. Online interactive dichotomous and matrix Lucid keys to genera and species are available at http://www.waspweb.org.

## Introduction

The Phaeogenini Förster, 1869 is a small tribe that encompasses about 10% of the species in the Ichneumoninae (Yu et al. 2012). These species are among the smallest in the subfamily (Perkins 1959, Selfa and Diller 1994). Phaeogenini often exhibit unusual habitus, which makes them easily recognizable within Ichneumoninae. They are characterized by circular spiracles on the propodeum, a feature which is also present in some Platylabini (Tereshkin 2009). The latter tribe, however, differs by a depressed petiole and a convex and fully carinate scutellum; in Phaeogenini, the petiole is rarely wider than high, and the scutellum is flat and at most partially carinate (Tereshkin 2009). This tribe currently includes 32 genera and about 400 species. Like the remaining Ichneumoninae, they are endoparasitoids of Lepidoptera.

Taxapad (Yu et al. 2012) reports 11 species of Phaeogenini from the Afrotropical region. This low number obviously underestimates their actual diversity in the region, reflecting how little has been published on this tribe. Heinrich’s review of African Ichneumoninae (1967) excludes the Phaeogenini. The only keys to genera currently available are those in Townes and Townes (1973) and Heinrich (1938), the latter dealing only with Malagasy species. Diller and Schönitzer (1999) provided a key to the species of Afrotropical *Heterischnus* Wesmael, 1845. The number of known Afrotropical species is here updated to 23, in 10 genera, of which one genus and 12 species are newly described. This revision encompasses predominantly the long–term collecting efforts undertaken in Madagascar (MNHN, ZMPA), Eastern and Southern Africa (SAMC, BMNH) and Democratic Republic of Congo (MRAC). It also includes the material collected during some occasional expeditions throughout Subsaharan Africa and stored in various museums in Europe and North America. Given the scarcity of these data (specimens are very rare in collections) and the high number of new taxa described here, we can, however, expect a significant number of additional species (and probably generic) discoveries in the future. The identification tools provided here and on www.waspweb.org are provided to facilitate the future documentation of this tribe in the region.

## Materials and methods

### Photographs

Specimens were point mounted on black, acid-free cards for examination (using a Leica M205C stereomicroscope with LED light source), photography and long–term preservation. Images were taken using the EntoVision® multiple–focus imaging system. This system combines a Leica® M16 microscope with a JVC® KY–75U 3–CCD digital video camera attached that feeds image data to a notebook computer. The program Cartograph®5.6.0 was then used to merge an image series (representing typically 10–15 focal planes) into a single in–focus image. Lighting was achieved using techniques summarized in Buffington et al. (2005), Kerr et al. (2008) and Buffington and Gates (2009). All images presented in this paper are available at http://www.waspweb.org.

### Specimens examined

Specimens from the major European and North American museums housing Afrotropical ichneumonids were studied. Specimens of Afrotropical Phaeogenini are, however, very rare in collections.

### List of depositories (abbreviations after Arnett et al. 1993)

BMNH Natural History Museum, London, UK (Gavin Broad)

CNCI Canadian National Collection of Insects, Ottawa, Canada (Andrew Bennett)

MNHN Muséum National d’Histoire Naturelle, Paris, France (Claire Villemant)

MNHU Museum fur Naturkunde, Humboldt Universität, Berlin, Germany (Franck Koch)

MRAC Muséum Royal de l’Afrique Centrale, Tervueren, Belgium (Eliane de Coninck)

NMKE National Museums of Kenya, Nairobi, Kenya (Martha Gikunga)

NMSA KwaZulu–Natal Museum, Pietermaritzburg, South Africa (Burgert Muller)

SAMC Iziko South African Museum, Cape Town, South Africa (Simon van Noort)

ZMPA Instytut Zoologiczny Polska Akademia Nauk, Warsaw, Poland (Tomasz Huflejt)

ZSMC Zoologische Staatsammlung, München, Germany (Stefan Schmidt)

Although all types from ZMPA are labeled as lectotypes designated by J. Sawoniewicz, the designation was not published. They are considered here as syntypes.

### Nomenclature and abbreviations

The morphological terminology follows Wahl and Sharkey (1993), but the wing venation nomenclature follows Gauld (1991). Most morphological terms are also defined on the HymAToL website (http://www.hymatol.org) and HAO website (http://portal.hymao.org/projects/32/public/ontology/). If not indicated otherwise, all geographical records are extracted from the Taxapad database (Yu et al. 2012). The following morphometric abbreviations are used (in order of appearance in the descriptions):

B: body length, from toruli to metasomal apex (mm)

A: antenna length, from base of scape to flagellar apex (mm)

F: front wing length, from tegula to wing apex (mm)

HdWi (head dorsal width index): maximal width / central length of head in dorsal view

HfWi (head frontal width index): maximal width / central height of head in frontal view

Di (dental index): length of upper mandibular tooth / length of lower tooth

Mi (malar line index): malar line / basal mandibular width

IOi (inter–oceller index): shortest distance between posterior ocelli / ocellus diameter

OOi (oculo–ocellar index): shortest distance between eye and posterior ocellus / ocellus diameter

Flin (length index of flagellomere n): length / width of flagellomere n

OTi (ovipositor sheath–tibia index): length of ovipositor sheath / length of hind tibia

Unless otherwise specified, the first three measurements (absolute measures) were measured on all specimens in the type series, with measurements from the primary type reported separately in brackets if necessary. The relative indices were measured on the primary type specimen only, or on one selected individual if no type could be examined (*Diadromus collaris* and *Lusius tenuissimus*).

## Results and discussion

### Key to genera and species of Afrotropical Phaeogenini

**Table:** 

	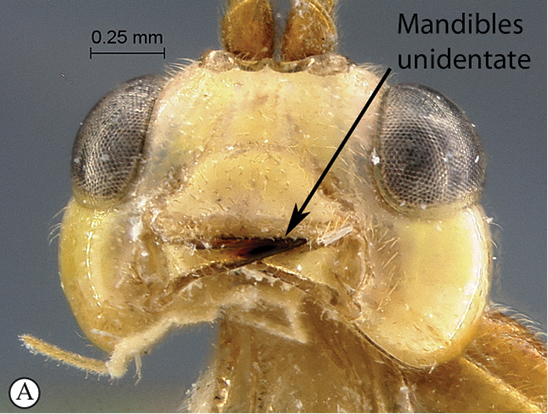	
1	Mandibles unidentate, falcate (A)	2
	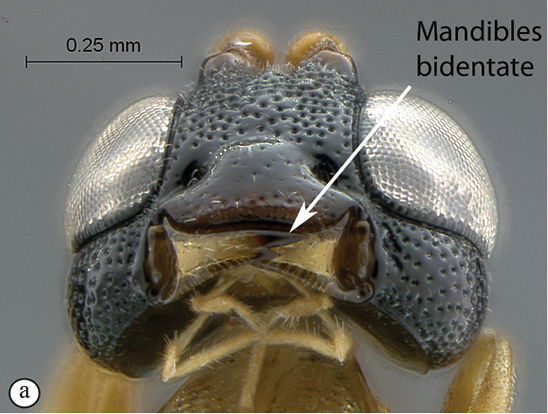	
–	Mandibles bidentate, shape various (a)	7
	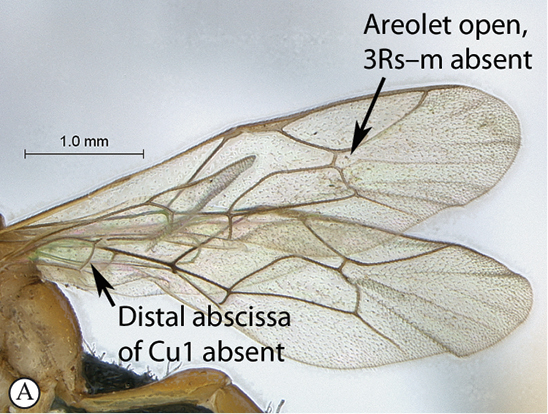	
2(1)	Areolet open, 3Rs–m absent (A); hind wing with distal abscissa of Cu1 absent (A)	*Lusius* Tosquinet, 1903 (3)
	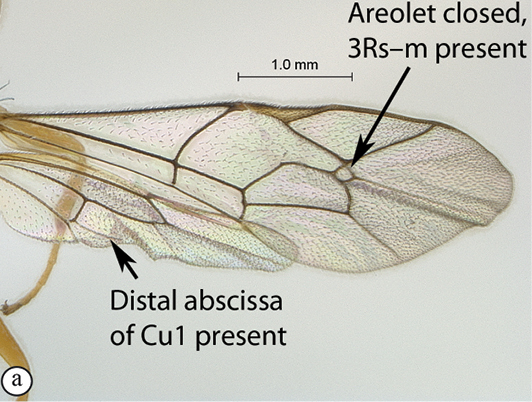	
–	Areolet closed, 3Rs–m present, sometimes non–tubular (a); hind wing with distal abscissa of Cu1 present, sometimes very faint (a)	*Heterischnus* Wesmael, 1859 (4)
	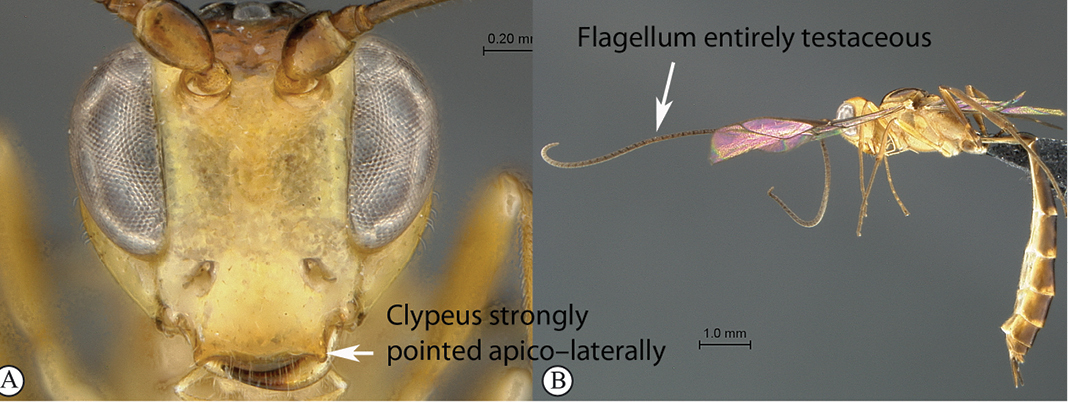	
3(2)	Clypeus wide, about twice as wide as high, strongly pointed apico–laterally (A); flagellum entirely testaceous (male unknown) (B)	*Lusius flummox* sp. n.
	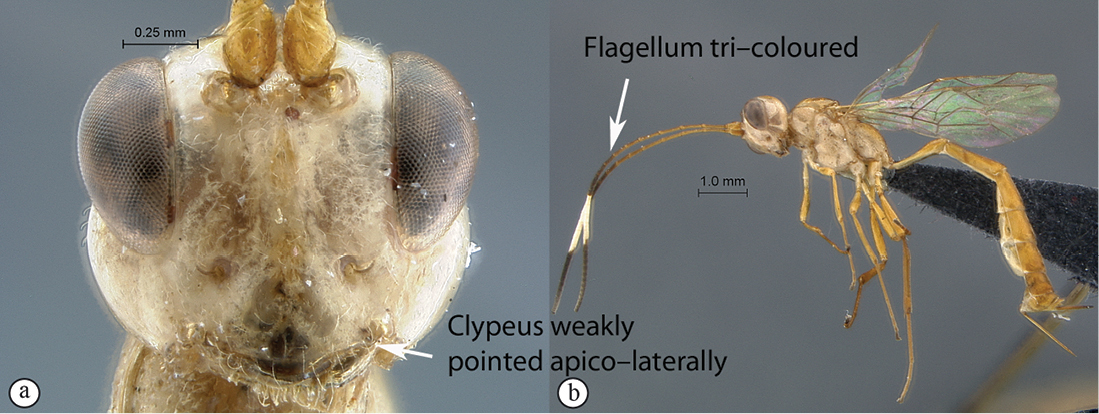	
–	Clypeus narrower, about one and a half times wider than high, weakly pointed apico–laterally (a); flagellum tri–coloured in both sexes: basally testaceous, medially white and apically black (b)	*Lusius tenuissimus* (Heinrich, 1938)
	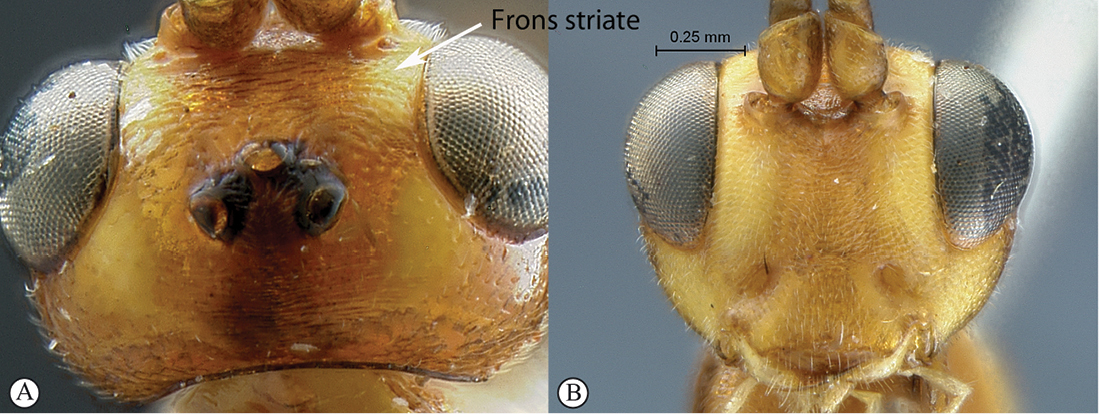	
4(2)	Frons transversely striate (A); head reddish and yellow (B)	*Heterischnus krausi* Schönitzer, 1999
	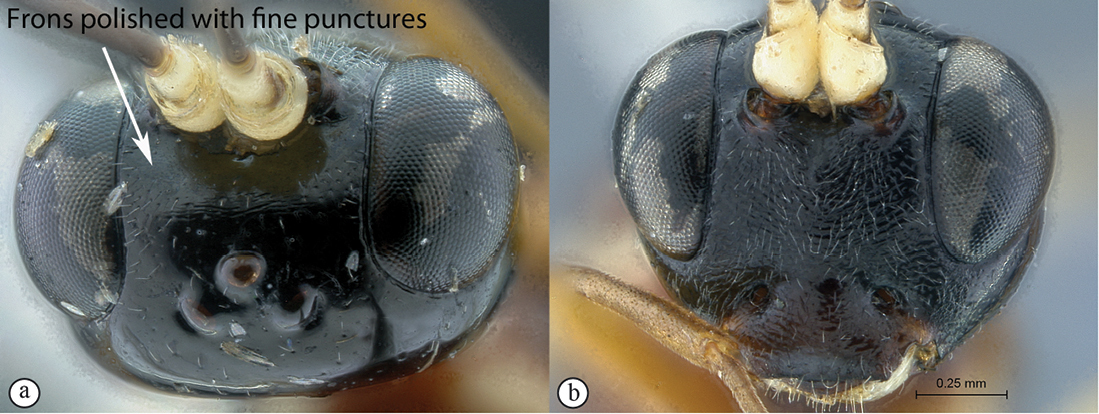	
–	Frons polished with fine isolated punctures (a); head black (b)	*Heterischnus olsoufieffi* (Heinrich, 1938)
	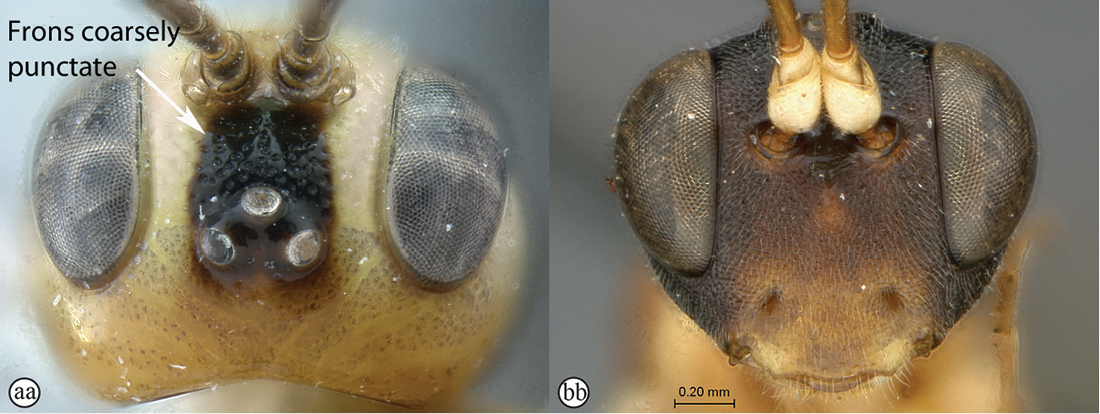	
–	Frons coarsely punctate (aa); coloration of head various (yellow, orange or red to black) (bb)	5
	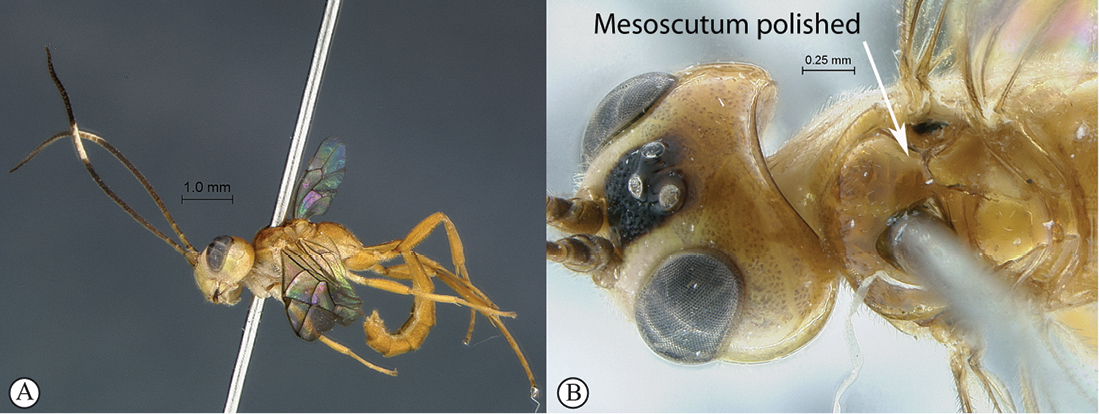	
5(4)	Colour entirely yellow to orange (A); mesoscutum polished with sparse punctation (B)	*Heterischnus mkomazi* sp. n.
	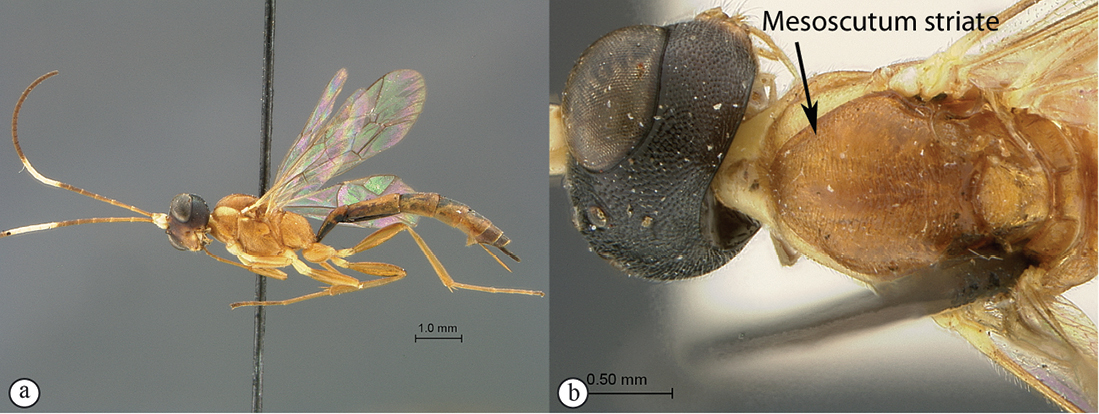	
–	Coloration darker, at least head black to dark red (a); mesoscutum transversely striate (b)	6
	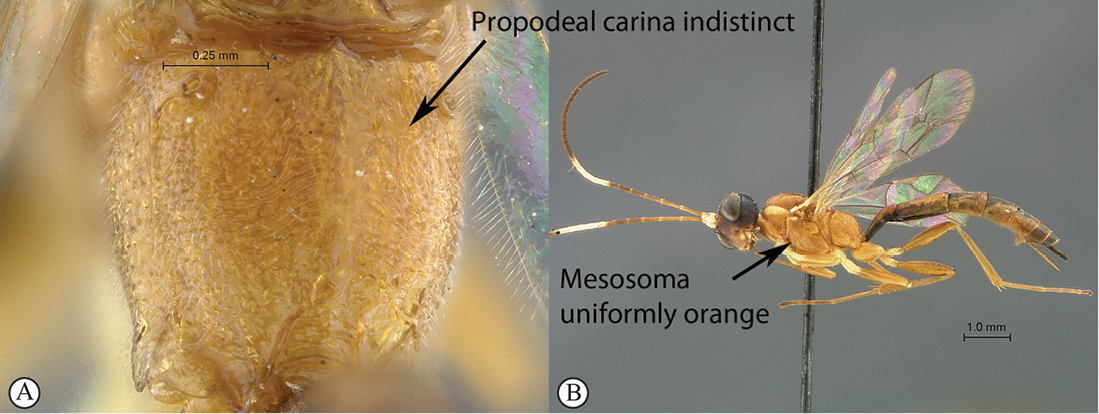	
6(5)	Propodeum without any distinct carina (A); mesosoma uniformly orange (B); flagellum of female with a white median ring (male unknown) (B); Southern Africa	*Heterischnus mfongosi* sp. n.
	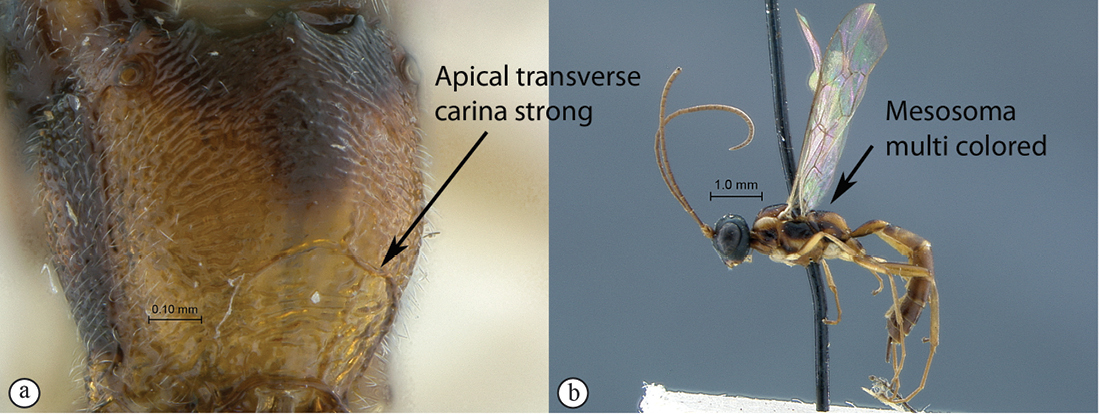	
–	Propodeum with apical transverse carina strong (a); mesosoma multi–colored, black, yellowish and testaceous (b); flagellum uniformly colored in both sexes (b); Eastern Africa	*Heterischnus africanus* (Heinrich, 1936)
	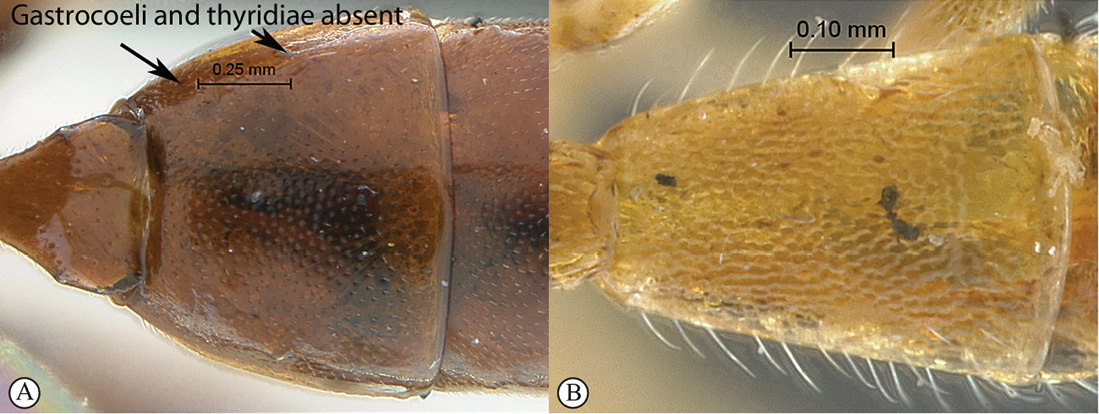	
7(1)	Metasomal tergite 2 with gastrocoeli and thyridiae totally absent (A, B)	8
	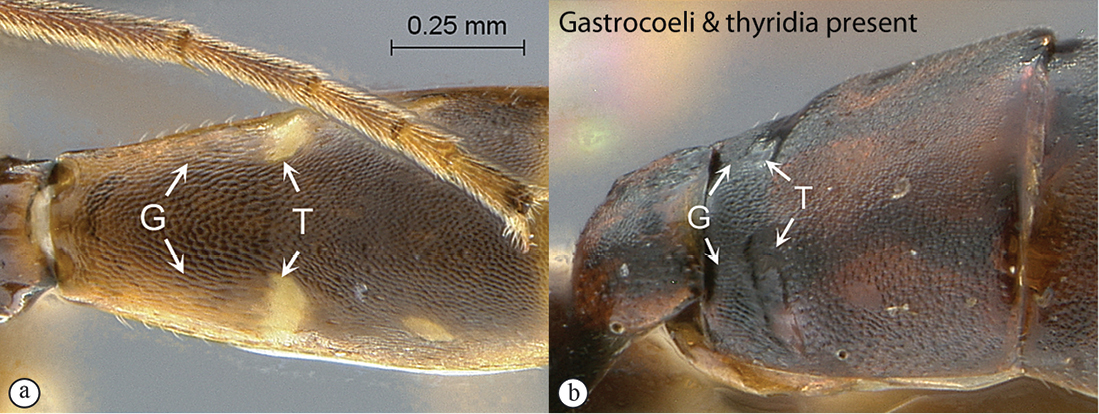	
–	Metasomal tergite 2 with gastrocoeli (G) present, faint to deep, and thyridiae (T) differentiated (a, b)	18
	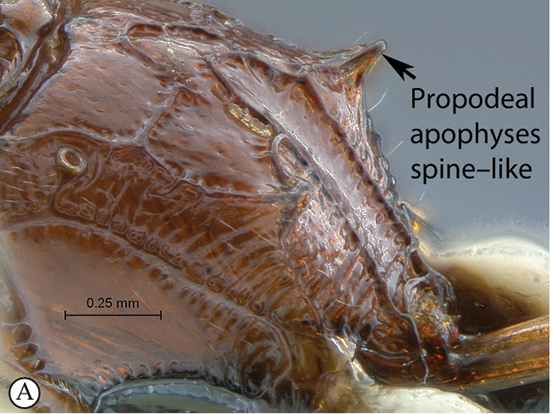	
8(7)	Propodeal apophyses strong, spine–like, at least as long as basally wide (A)	9
	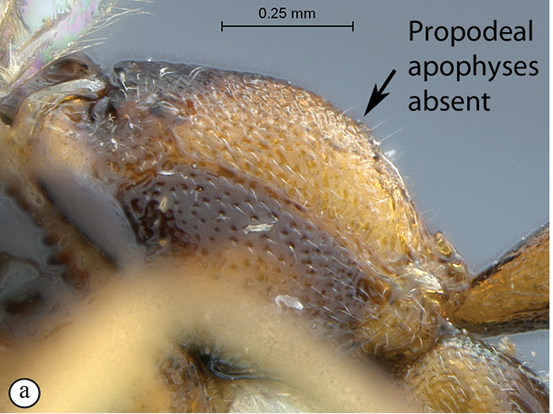	
–	Propodeum without spine–like apophyses, or apophyses hardly distinct (a)	10
	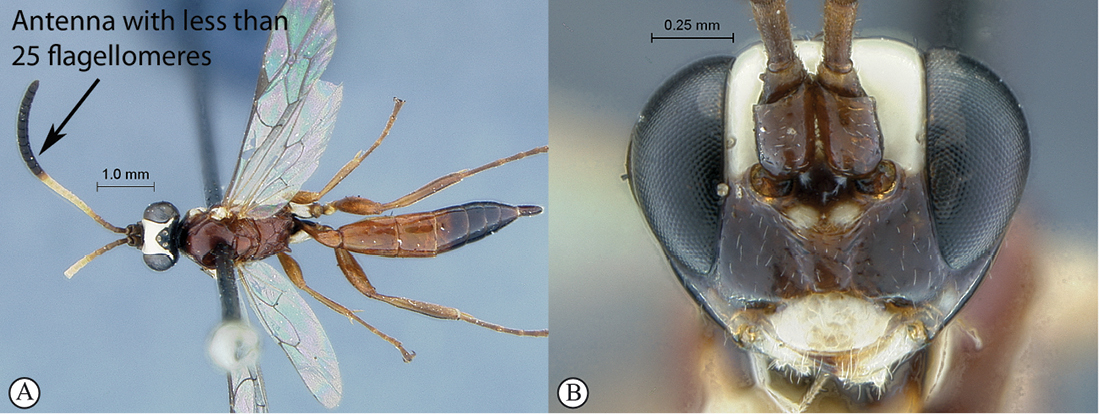	
9(8)	Antenna short with less than 25 flagellomeres (A); head mostly dark brown to black with mandibles, clypeus and frons pale yellow (B)	*Hoplophaeogenes curticornis* Heinrich, 1938
	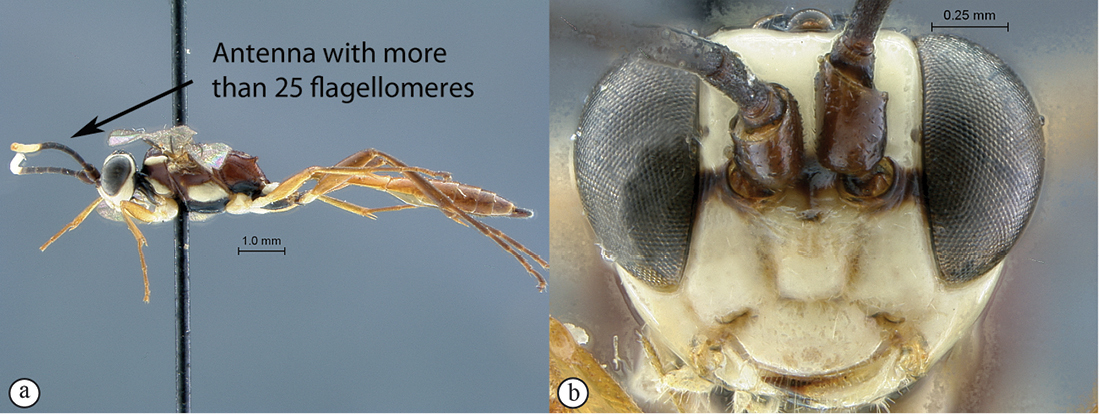	
–	Antenna longer with more than 25 flagellomeres; head mostly pale yellow with occiput and vertex black	*Hoplophaeogenes amoenus* Heinrich, 1938
	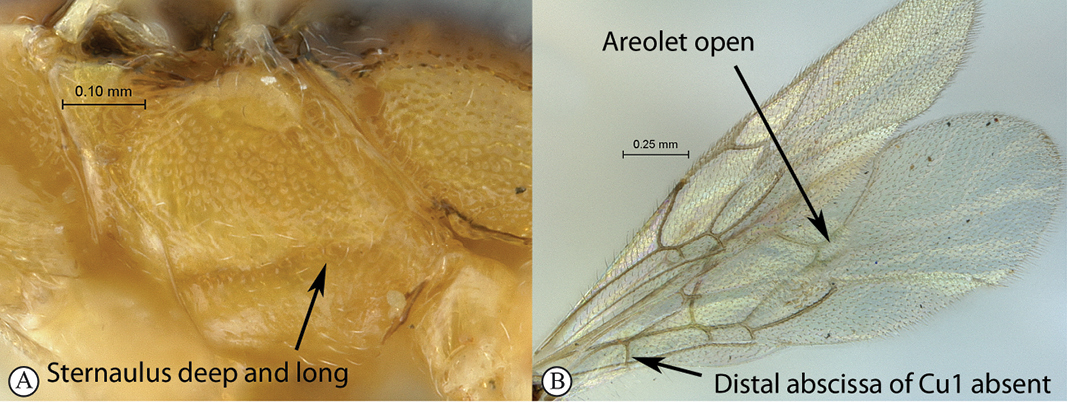	
10(8)	Sternaulus deep and long, reaching beyond mid–length of mesopleuron (A); areolet open, 3Rs–m absent (B); hind wing with distal abscissa of Cu1 absent (B)	*Arearia* Seyrig, 1952 (11)
	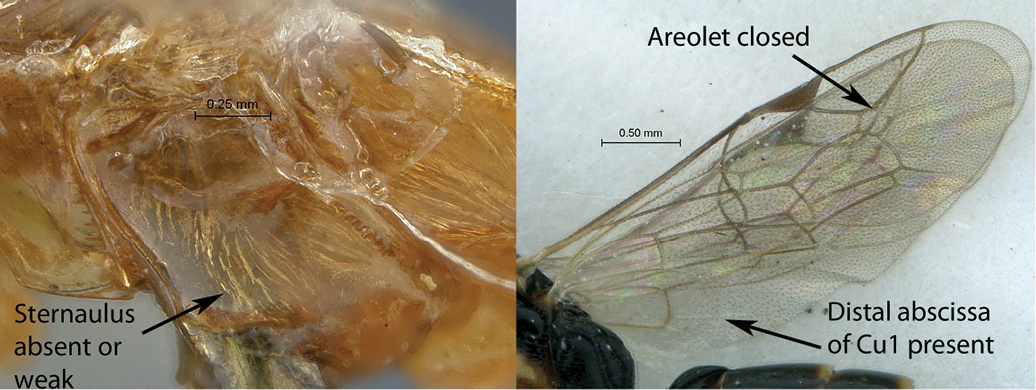	
–	Sternaulus absent or at least much weaker and shorter (a); areolet closed, 3Rs–m present (b); hind wing with distal abscissa of Cu1 present, sometimes non pigmented (b)	12
	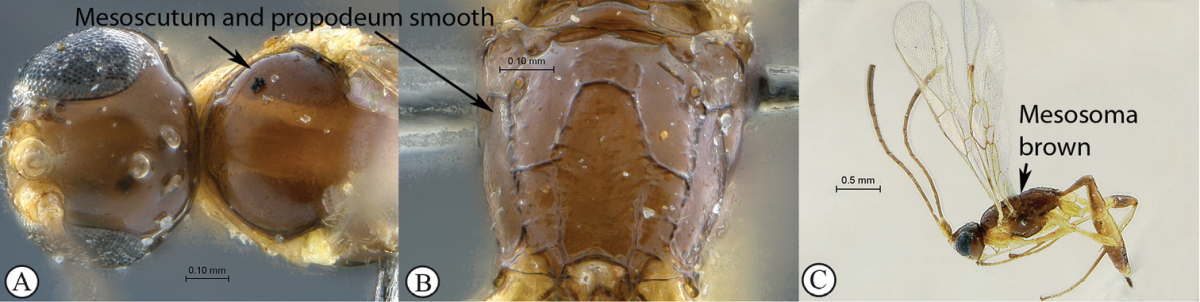	
11(10)	Mesoscutum and propodeum smooth (A–B); mesosoma mostly brown with pronotum yellow (C); Madagascar	*Arearia paradoxa* Seyrig, 1952
	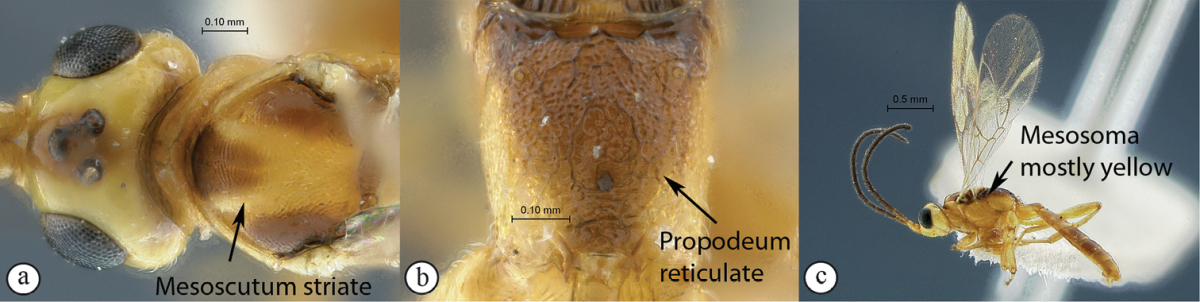	
–	Mesoscutum transversely striate, propodeum reticulate (a–b); mesosoma mostly yellow, dorsally black (c); South Africa	*Arearia oxymoron* sp. n.
	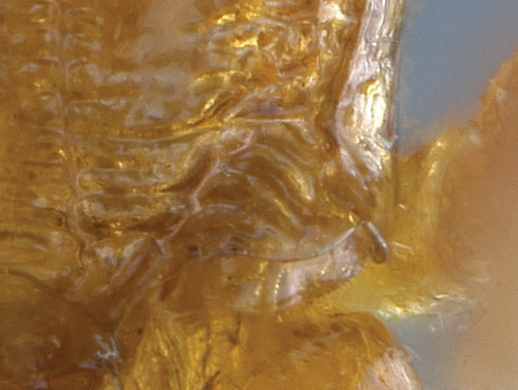	
12(10)	Propodeum smooth, unsculptured (at least anteriorly) with median areas fused into one single mid–longitudinal area (A)	*Chauvinia* Heinrich, 1938 (13)
	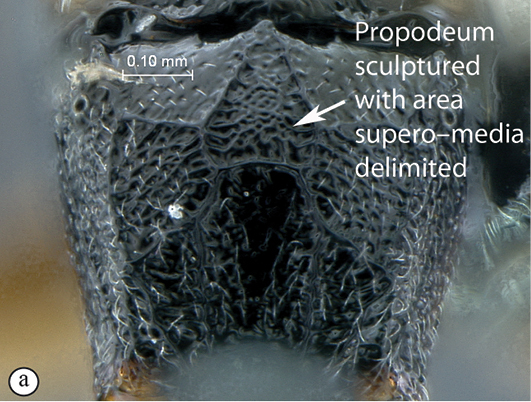	
–	Propodeum coarsely sculptured with area superomedia delimited (a); mainland Africa	*Dicaelotus* Wesmael, 1845 (15)
	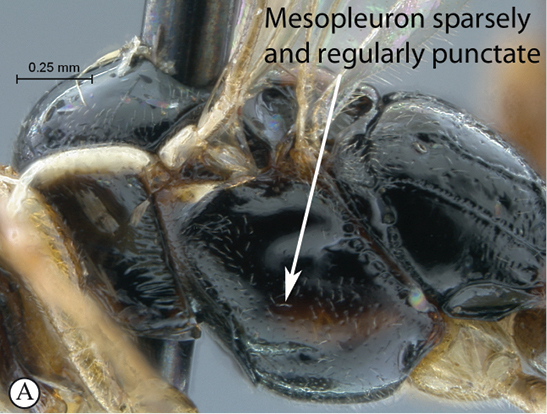	
13(12)	Mesosoma mostly black with mesopleuron sparsely and regularly punctate (A); tropical Africa	*Chauvinia nyanga* sp. n.
	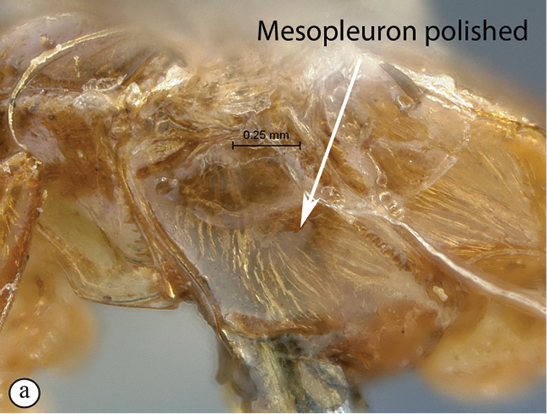	
–	Mesosoma yellowish orange, mesopleuron polished (a); Madagascar	14
	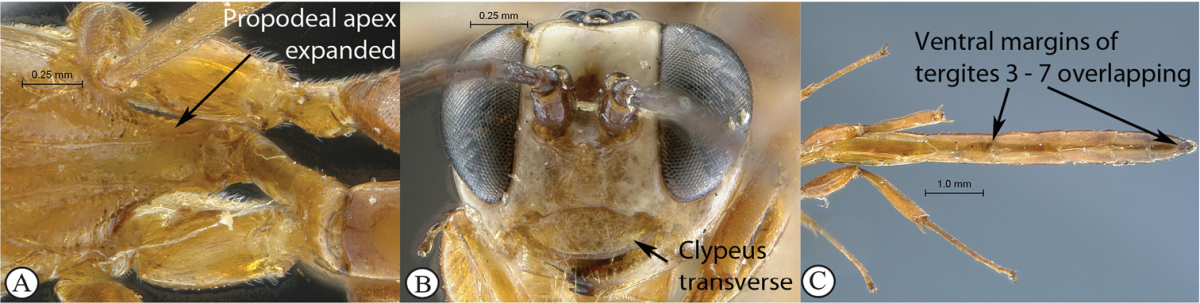	
14(13)	Apex of propodeum expanded between hind coxae, reaching half their length (A); clypeus strongly transverse (Ci < 3.0) (B); metasoma of female strongly elongate, longer than hind leg, with ventral margins of tergites 3 and following overlapping (C)	*Chauvinia pelecinoides* Heinrich, 1938
	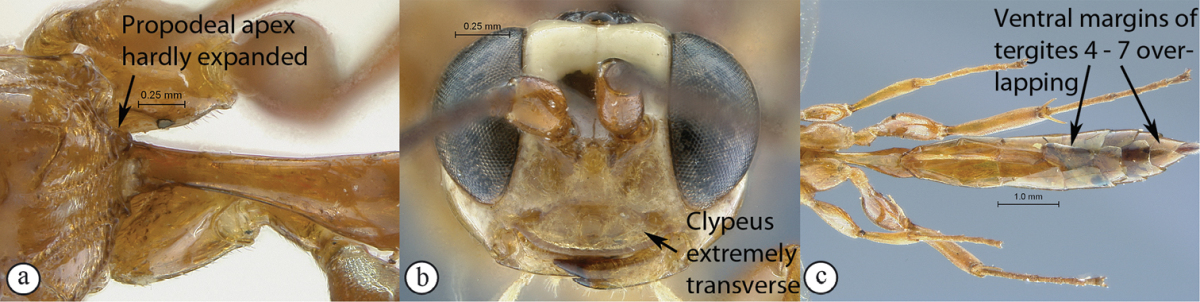	
–	Apex of propodeum hardly expanded between hind coxae (a); clypeus extremely transverse (Ci > 3) (b); metasoma of female shorter, shorter than hind leg, with ventral margins of tergites 4 and following overlapping (c)	*Chauvinia nitida* Heinrich, 1938
	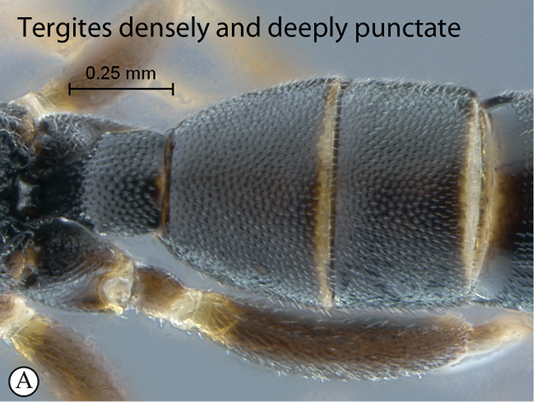	
15(12)	Tergite 2 and following densely and deeply punctate (A)	16
	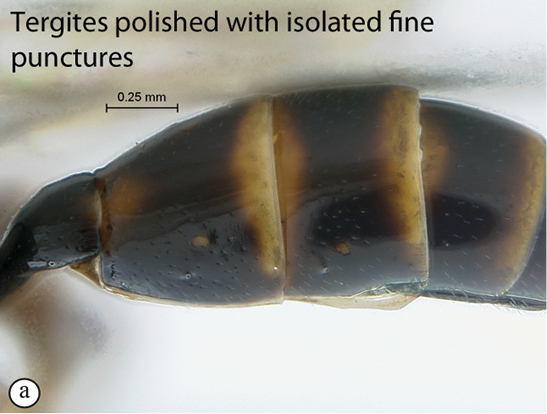	
–	Tergite 2 shallowly punctate, following tergites alutaceous to smooth with isolated fine punctures (a)	17
	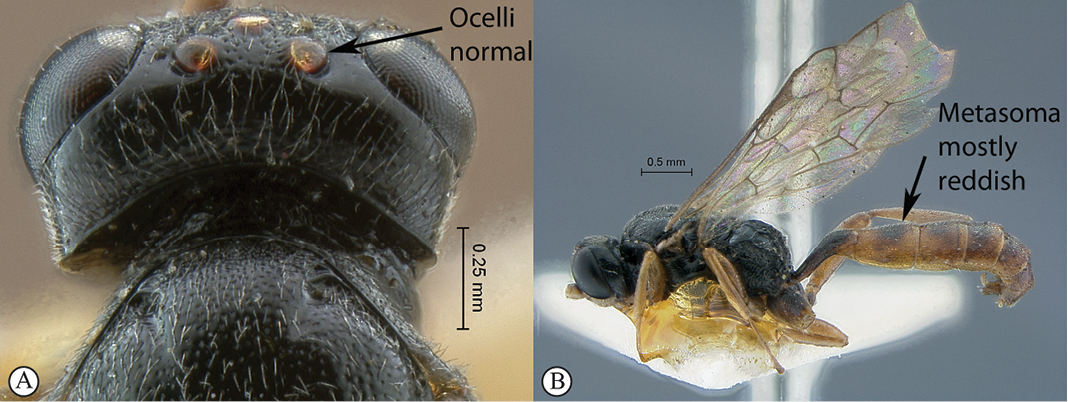	
16(15)	Ocelli moderately sized, IOi < 2.0 (A); metasoma mostly reddish (B)	*Dicaelotus cariniscutis* (Cameron, 1906)
	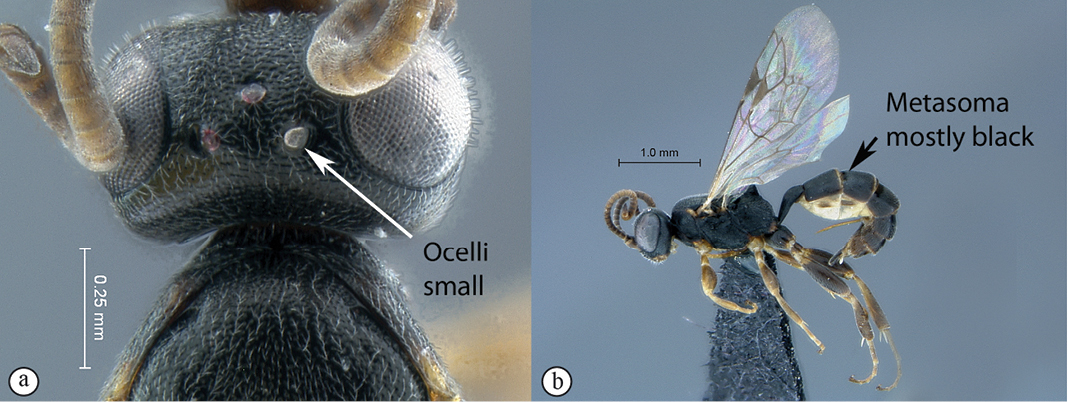	
–	Ocelli small, IOi ≥ 2.0 (a); metasoma mostly black (b)	*Dicaelotus asantesana* sp. n.
	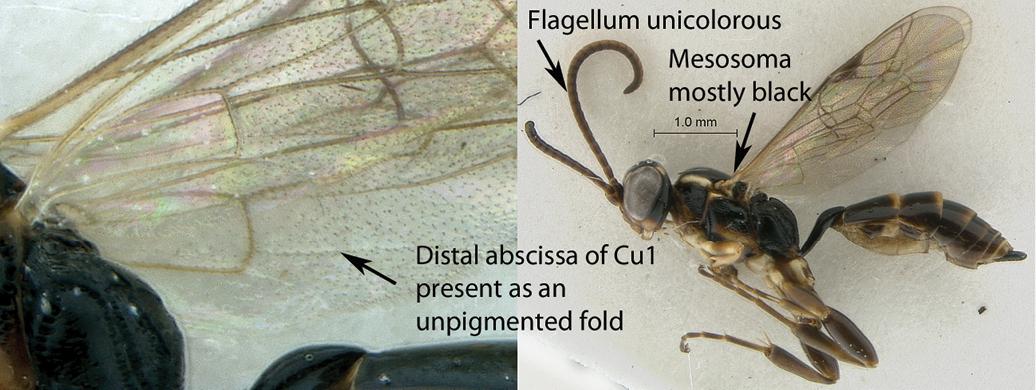	
17(15)	Hind wing with distal abscissa of Cu1 present as an unpigmented fold (A); mesosoma mostly black with one pale yellow longitudinal stripe on pronotum and another one on mesopleuron, mesopleuron and pronotum ventrally fading to reddish (B); flagellum unicolorous (male unknown) (B)	*Dicaelotus hoerikwaggoensis* sp. n.
	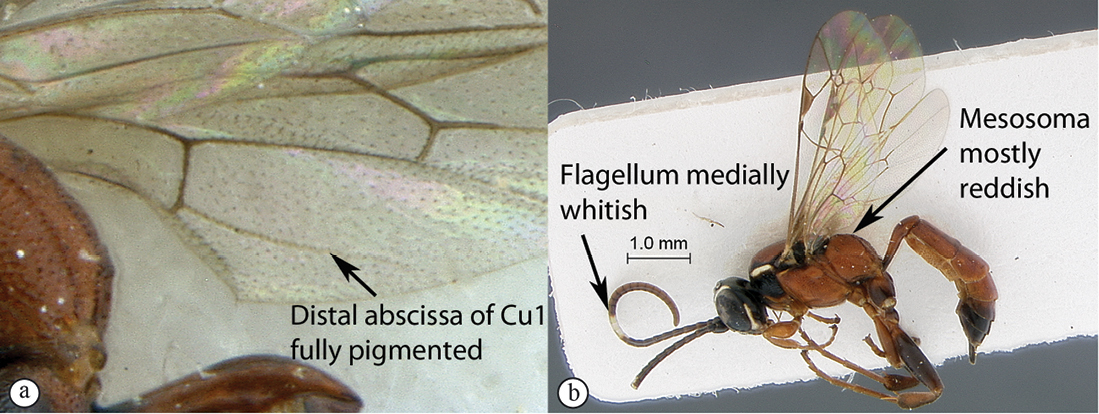	
–	Hind wing with distal abscissa of Cu1 fully pigmented (a); mesosoma mostly reddish, pronotum black with a yellow longitudinal stripe, subtegular ridge and axillar troughs black (b); flagellum medially whitish (male unknown) (b)	*Dicaelotus tablemountainensis* sp. n.
	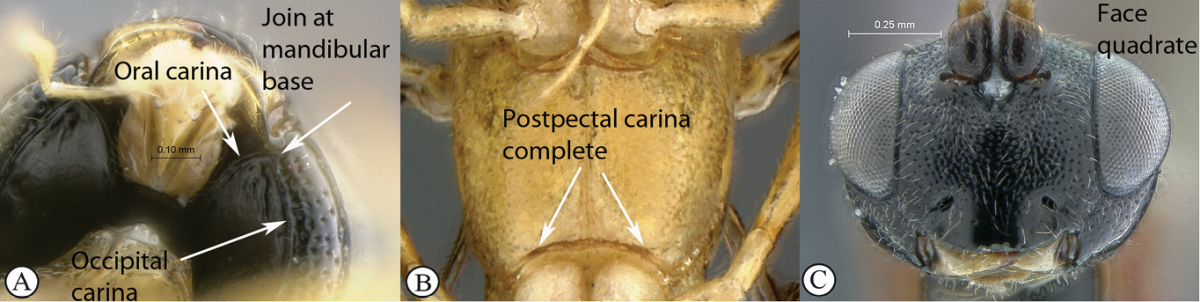	
18(7)	Occipital and hypostomal carinae joining at mandibular base, the junction separated from mandibular basis by less than carina width (A); postpectal carina complete (B); face quadrate without distinct mid–longitudinal bulge (C)	19
	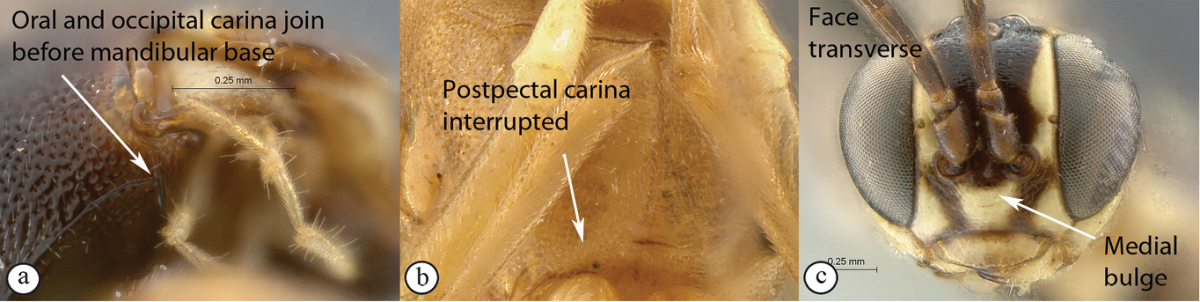	
–	Hypostomal and occipital carinae joined distinctly above mandibular base (a); postpectal carina ventrally widely interrupted in front of mid coxae (b); face short and transverse with a strong mid–longitudinal bulge laterally limited by grooves (c)	*Tycherus* Förster, 1869 (21)
	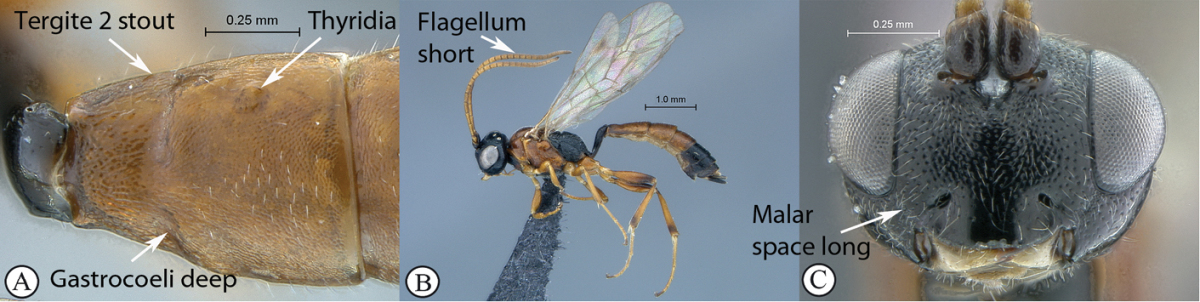	
19(17)	Metasomal tergite 2 stout, less than 1.5× longer than apically wide (A); gastrocoeli deep, thyridiae concolourous with remainder of tergite (A); flagellum shorter than fore wing (B); malar space long (Mi > 0.5) (C)	*Diadromus collaris* (Gravenhorst, 1829)
	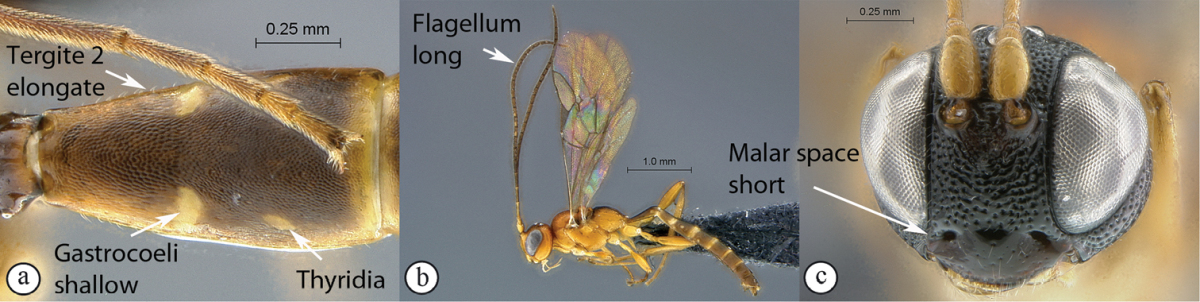	
–	Metasomal tergite 2 elongate, more than 2× longer than apically wide (a); gastrocoeli very shallow, thyridiae lighter than remainder of tergite (a); flagellum slender, longer than fore wing (b); malar space short (Mi < 0.5) (c)	*Kibalus* gen. n. (20)
	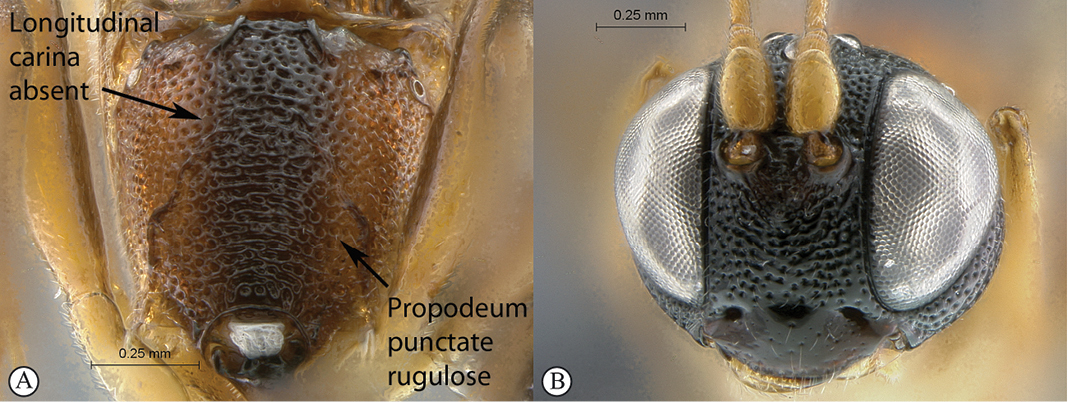	
20(19)	Propodeum without any longitudinal carina, no area defined; punctate rugulose (A); head mostly black (B)	*Kibalus toro* sp. n.
	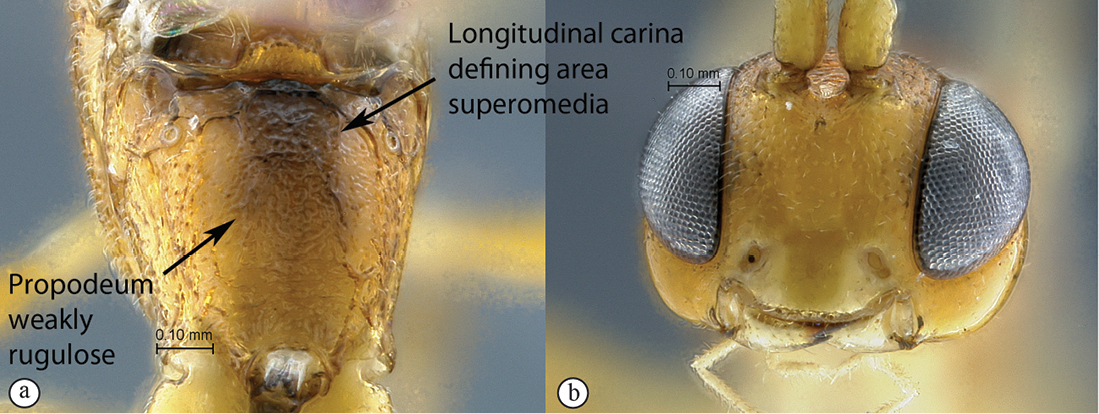	
–	Propodeum with longitudinal carina present, area superomedia complete; weakly rugulose (a); head mostly yellowish–orange (b)	*Kibalus mubfs* sp. n.
	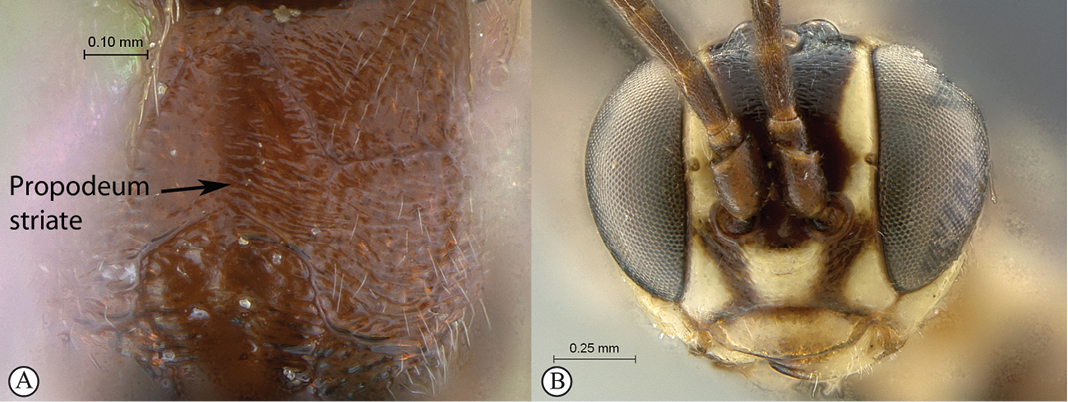	
21(18)	Propodeum striate (A); head mostly yellow in frontal view (B)	*Tycherus amatola* sp. n.
	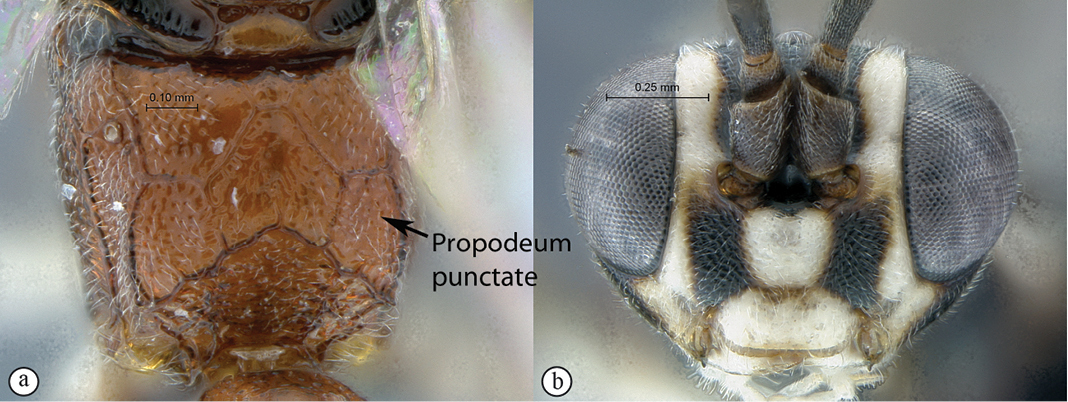	
–	Propodeum punctate (a); head mostly white in frontal view (b)	*Tycherus nardousberg* sp. n.

### Descriptions

#### Arearia

Seyrig, 1952

http://species-id.net/wiki/Arearia

##### Diagnosis.

Highly distinctive genus, characterized by sternauli deeply impressed and extending beyond mid–length of mesopleuron. Head polished and smoothly sculptured, dorsally expanded and flattened, somewhat triangular in profile; mandible bidentate, upper tooth 1.5–2.0× longer than lower tooth; subocular groove present; clypeus transverse, its ventral margin regularly convex; flagellum of female regularly enlarged toward apex; occipital and hypostomal carinae joining at mandibular basis; epomia weak; notaulus weakly impressed; postpectal carina complete and strong; propodeum moderately elongate, regularly curved in profile view, propodeal carination well developed to about absent; fore wing with areolet open, hind wing with 1/Cu&cu–a straight and distal abscissa of Cu1 absent; tarsal claws simple; gastrocoelus and thyridium indistinct; ovipositor distinctly extending beyond apex of metasoma.

Because of its strong and long sternaulus, this genus was first placed in the Cryptinae by Seyrig (1952), and subsequently moved to the Ichneumoninae by Townes (1971).

##### Species richness and distribution.

The genus was previously only known from Madagascar, with a single species. Here we report a new species from South Africa.

#### Arearia paradoxa

Seyrig, 1952

http://species-id.net/wiki/Arearia_paradoxa

Figs 1 –2

##### Material examined.

**HOLOTYPE** Female: Madagascar, Trafanaomby, VIII.40, A. Seyrig (MNHN EY8804).

##### Diagnosis.

Testaceous overall with pronotum, base of antenna and legs yellow; mandible triangular, strongly tapered toward apex, teeth small; malar line short; torulus on a distinctly bulging platform; ocellar triangle reduced, equilateral; antenna with 21 flagellomeres (Seyrig 1952); mesosoma smooth and polished, including sternaulus; propodeal carination well developed, areae superomedia and petiolaris fused, both forming a concave surface; legs elongate; metasoma superficially punctate, gastrocoelus indistinct; ovipositor extending distinctly beyond metasomal apex. B 2.9; A NA; F 2.5; HdWi 1.3; HfWi 1.3; Ci 1.5; Mi 0.3; Di 1.6; IOi 0.9; OOi 2.1; Fli_1_3.1; Fli_15_1.5; OTi 0.1. Male unknown.

##### Distribution.

Madagascar (Toliara province) (Seyrig 1952).

**Figure 1.**
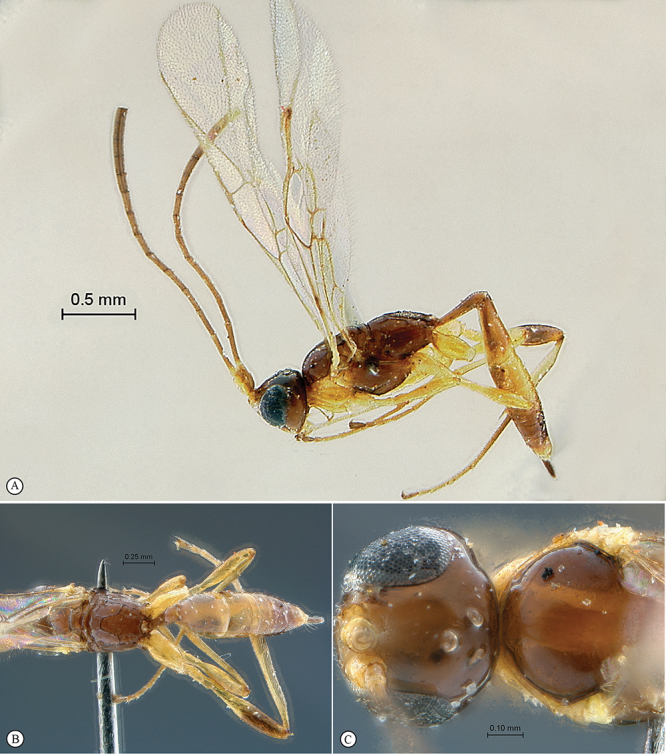
*Arearia paradoxa* Holotype female. **A** habitus lateral view **B** habitus dorsal view **C** head, mesosoma, dorsal view.

**Figure 2.**
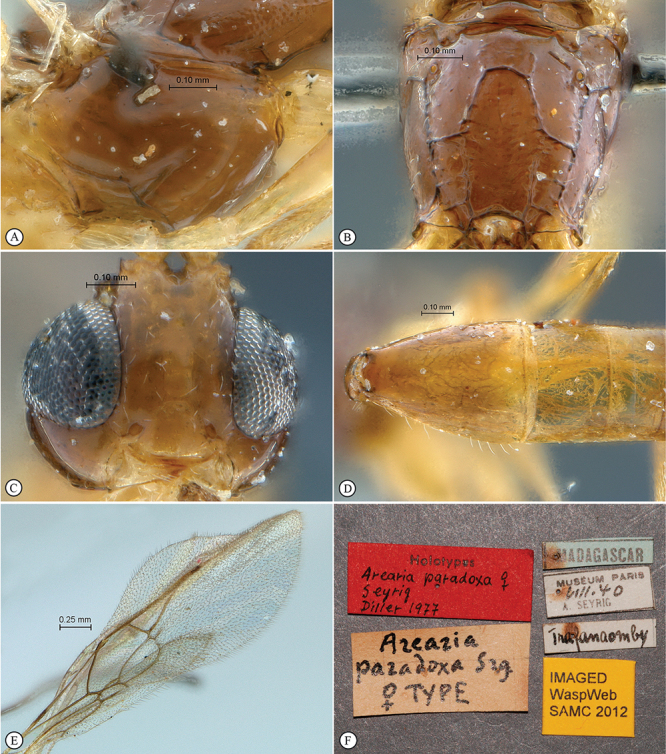
*Arearia paradoxa* Holotype female. **A** mesopleuron, lateral view **B** propodeum dorsal view **C** head, anterior view **D** tergites 2–4, dorsal view **E** wings **F** data labels.

#### Arearia oxymoron

Rousse & van Noort sp. n.

http://zoobank.org/C524CEEB-F2B6-40F3-9480-7769BA7C8D68

http://species-id.net/wiki/Arearia_oxymoron

Figs 3 –4

##### Type material.

**HOLOTYPE** Male: South Africa, R.E. Turner, Brit. Mus. 1931–37, Cape Province, Somerset East, 10–22.xii.1930 (BMNH). **PARATYPES** 1 male: South Africa, R.E. Turner, Brit. Mus. 1927–117, Orange F. State, Harrismith, Feb 1927 (BMNH); 3 males: same label data except: Brit. Mus. 1927–147, March 1–20 1927 (BMNH).

##### Diagnosis.

Female unknown. Male: bright yellow overall with variable dorsal testaceous to black markings on mesosoma and metasoma; face and clypeus strongly convex in profile; toruli on a weak platform; malar line long; ocellar triangle equilateral; antenna with 19–20 flagellomeres; mesosoma laterally punctate but scutellum and most of pronotum smooth; sternaulus crenulate, extending to mid coxa; mesosoma dorsally alutaceous but propodeum coarsely reticulate with carination strongly reduced; metasoma deeply punctate–reticulate. HdWi 1.3; HfWi 1.3; Ci 1.6; Mi 0.9; Di 2.2; IOi 1.7; OOi 1.7; Fli_1_4.2; Fli_15_1.7; OTi NA.

##### Description.

MALE (5 specimens). B 2.5–2.8; A 1.9–2.1; F 2.1–2.3 (Holotype: B 2.7; A: 1.9; F 2.2).

*Color*. Bright yellow with variable testaceous to black dorsal parts: vertex, flagellum, upper occiput, mesoscutal lobes, axillar area around scutellum, propodeum, and all tergites but their apical margin; wings hyaline, venation light yellow.

*Head*. Head subspherical, posteriorly truncate in profile; clypeus quite smooth, polished, transverse, its ventral margin regularly rounded; malar line long, subocular sulcus deep; face strongly protruding, bearing toruli on a weak platform, about smooth with some faint oblique striations laterally; frons, vertex and temple quite smooth; ocellar triangle equilateral; temple strongly rounded, head distinctly swollen behind eyes; antenna with 19–20 flagellomeres.

*Mesosoma*. Mesosoma elongate, slightly depressed dorso–ventrally; pronotum, mesopleuron and metapleuron densely punctate, almost punctate–reticulate, with speculum smooth; sternaulus thin, deep and long, reaching mid coxa; mesoscutum smoothly sculptured, anteriorly transversely striate, posteriorly punctate–alutaceous to punctate–reticulate; notaulus hardly distinct; scutellum quite smooth; propodeum coarsely reticulate without distinct carination.

*Metasoma*. All tergites deeply scaly punctate–reticulate, but posterior tergites sometimes variably smooth.

FEMALE. Unknown.

##### Etymology.

In line with Seyrig’s derivation of his specific epithet *paradoxa*, the new species name refers to the rhetoric figure of speech pinpointing the apparent incompatibility between Ichneumoninae and strong sternauli.

##### Distribution.

South Africa (Eastern Cape and Free State).

**Figure 3.**
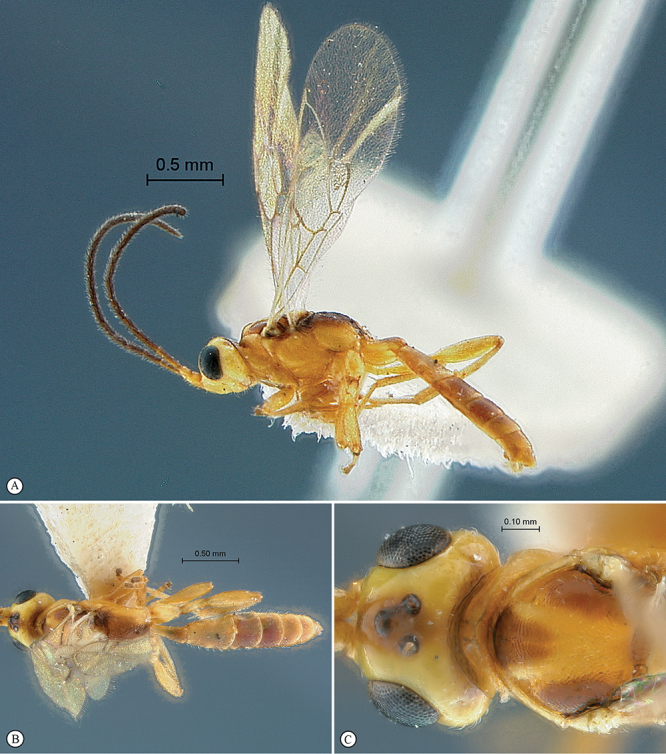
*Arearia oxymoron* Holotype male. **A** habitus lateral view **B** habitus dorsal view **C** head, mesosoma, dorsal view.

**Figure 4.**
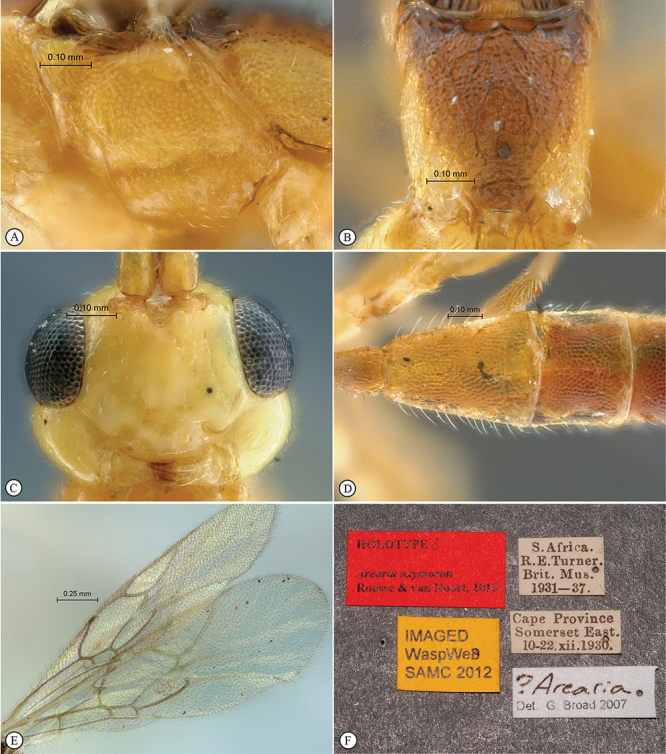
*Arearia oxymoron* Holotype male. **A** mesopleuron, lateral view **B** propodeum dorsal view **C** head, anterior view **D** tergites 2–4, dorsal view **E** wings **F** data labels.

#### Chauvinia

Heinrich, 1938

http://species-id.net/wiki/Chauvinia

Chauviniella Heinrich, 1938: 125

##### Diagnosis.

*Chauvinia* is a highly distinctive genus, mainly characterized by the conformation of the propodeum and the metasoma. Mandible bidentate; clypeus strongly transverse, its ventral margin sharp and more or less regularly rounded; flagellum of female enlarged from middle; temples moderately swollen behind eyes; occipital and hypostomal carinae joining above mandibular base; epomia present, moderate; notaulus indistinct; propodeum elongate, in profile slightly and regularly rounded to uniformly sloping backwards in a single plane; median areas of propodeum fused into a single mid–longitudinal area, lateral areas fully carinate; postpectal carina interrupted in front of mid coxae; fore wing with areolet pentagonal, closed; hind wing with distal abscissa of Cu1 present, unpigmented; tarsal claws simple; metasoma of female elongate to strongly elongate, ventral margins of apical tergites overlapping, hiding sternites; metasoma of male not so unusually modified; gastrocoelus and thyridium indistinct; ovipositor sheath wide, barely extending beyond metasomal apex.

##### Species richness and distribution.

Strictly Afrotropical genus, with three species of which one is newly described here.

#### Chauvinia nitida

(Heinrich, 1938)

http://species-id.net/wiki/Chauvinia_nitida

Figs 5 –6

Chauviniella nitida Heinrich, 1938: 125

##### Material examined.

**SYNTYPES 1 female:** Madagaskar, Rogez, 600 m, I–II.1931 leg. A. Seyrig (ZMPA); 1 male: same label data except for: V–VI.1931 (ZMPA).

##### Diagnosis.

Female: head more or less extensively yellowish–orange ventrally, remainder of head pale yellow with vertex and occiput black; remainder of body uniformly yellowish–orange, apex of metasoma sometimes infuscate; antenna short with 23 flagellomeres, basally dark testaceous, medially white and apically dark brown; head strongly transverse in frontal view; upper mandibular tooth 3× as long as lower tooth; clypeus nearly 4× wider than high; head and body very faintly sculptured, mostly smooth and polished; propodeal carination moderate; metasoma slightly elongate but shorter than hind leg; tergite 4 and following ventrally overlapping. B 7.8–8.9; A 4.8–5.2; F 4.6–4.9 (holotype: B 8.9; A 5.2; F 4.9); HdWi 1.6; HfWi 1.4; Ci 3.9; Mi 0.5; Di 3.1; IOi 1.1; OOi 0.7; Fli_1_ 3.8; Fli_15_ 0.8; Fli_21_ 0.7; OTi 0.2. Male: head without yellowish–orange ventral coloration; metasoma brown, tergites 2–3 basally yellow, all metasomal tergites apically yellow margined; flagellum entirely dark brown, slenderer; otherwise similar to female. B 6.4; A 5.4; F 3.4.

##### Distribution.

Madagascar (Toamasina and Toliara provinces) (Heinrich 1938).

**Figure 5.**
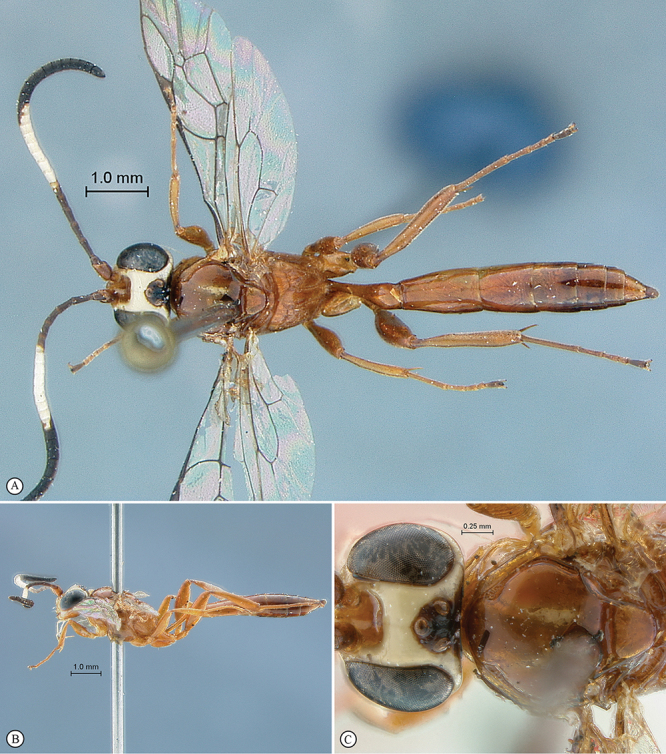
*Chauvinia nitida* Holotype female. **A** habitus dorsal view **B** habitus lateral view **C** head, mesosoma, dorsal view.

**Figure 6.**
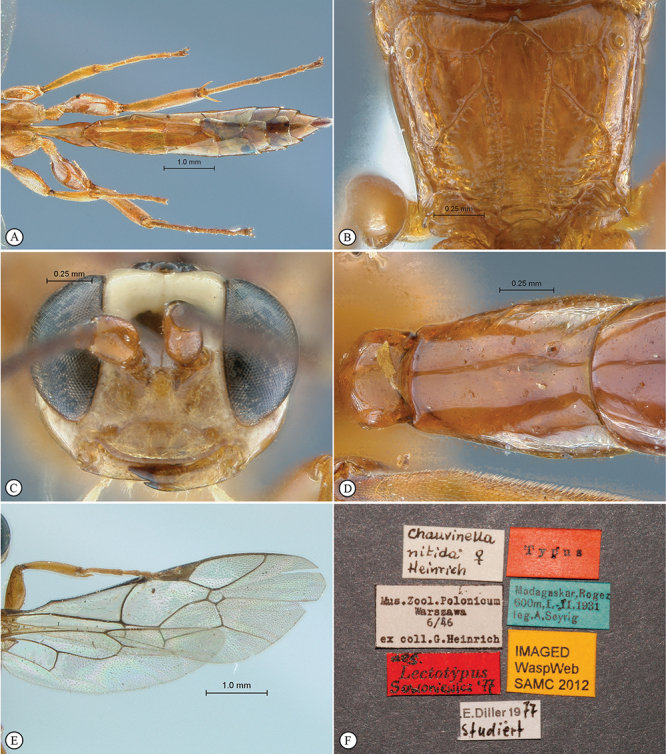
*Chauvinia nitida* Holotype female. **A** metasoma, ventral view **B** propodeum dorsal view **C** head, anterior view **D** tergites 2–3, dorsal view **E** wings **F** data labels.

#### Chauvinia nyanga

Rousse & van Noort sp. n.

http://zoobank.org/24F33A2D-2D37-465A-A748-8CD2F1AAFB4B

http://species-id.net/wiki/Chauvinia_nyanga

Figs 7 –8

##### Type material.

**HOLOTYPE** Female: Zimbabwe Nyanga Nat. Park, 7.xii.1993 18°18'S, 32°48'E leg.: F. Koch (MNHU). **PARATYPES** 1 male: same label data (MNHU); 1 female: Congo Belge, P.N.A. [Albert National Park] 18–III–1954 P. Vanschuytbroeck & H. Synave 7764 Secteur Tschiaberimu Kirungu (lieu–dit) 2.720m (MRAC); 1 male: Uganda Ruwenzori Range xii.1934–i.1935 B.M.E. Afr. Exp. B.M. 1995–203, Namwamba Valley, 6,500 ft. F.W.Edwards (BMNH). **Other material:** 1 female Kivu: Kavimira (Uvira) (à la lumière) XII–1954 G. Marlier (MRAC); 1 male N. Lac Kivu: Rwankwi XII–1951 J.V. Leroy (MRAC).

##### Diagnosis.

Head rufo–testaceous, dorsally black with upper orbits partially to totally yellow; female with flagellomeres 6–8 posteriorly pale yellow; mesosoma mostly black; legs mostly testaceous with coxae partially to totally pale yellow; metasoma basally black, often lightening towards apex; head mostly smooth with some sparse punctures on face and above toruli level; pronotum and metapleuron smooth, mesopleuron sparsely punctate, mesonotum finely and sparsely punctate, propodeum basally smooth and apically transversely rugose–striate; metasoma finely and sparsely punctate; face strongly bulging medially, clypeus strongly transverse; propodeal carination moderate; metasoma elongate but shorter than hind leg. HdWi 2.2; HfWi 1.1; Mi 0.5; Ci 2.9; Di 3.2; IOi 1.5; OOi 1.0; Fli_1_ 1.8; Fli_15_ 0.7; Fli_22_ 0.6; OTi 0.1.

##### Description.

FEMALE (2 specimens). B 7.0–7.3; A 2.5–2.7; F 3.6–3.9 (Holotype B 7.3; A 2.7; F 3.9).

*Color*. Head rufo–testaceous, dorsally black from torulus level but a variable part part of upper orbits yellow; antenna testaceous fading to dark brown with posterior half of flagellomeres 6–8 pale yellow; mesosoma black with upper margin of pronotum pale yellow, and tegula, anterior marin of pronotum, scutellum and a longitudinal stripe on mesopleuron sometimes lightening to pale yellow; legs light testaceous, hind leg darker, with fore and mid coxae pale yellow and hind coxa variously pale yellow maculated; tergite 1 black, remainder of metasoma variously lightening from dark brown to orange, apical margins of tergites 1–3 often yellow; wings hyaline, venation light brown.

*Head*. Entirely polished with few hairs but the strongly setose labral margin; face very short and strongly transverse, medially strongly bulging, sparsely punctate but sometimes distinctly smoother medially; clypeus highly transverse, smooth but some punctures dorso–laterally, ventral margin sharp and subtruncate; malar line short, finely granulate; frons, vertex and temple sparsely punctate; ocellar triangle wider than long; temple slightly then strongly rounded, head barely constricted behind eyes; antenna stout with 23 flagellomeres, flagellomeres of apical half distinctly wider than long.

*Mesosoma*. Entire mesosoma polished.Pronotum quite smooth with some smooth striations ventrally; mesopleuron finely and sparsely punctate, speculum smooth; metapleuron almost to quite smooth; mesoscutum and scutellum smooth with fine and sparse punctures; notaulus indistinct; propodeum regularly rounded in profile, anteriorly sparsely punctate, posterior half transversely rugose–stiate; carination moderately strong, characteristic of the genus.

*Metasoma*. Elongate but shorter than hind leg; all tergites polished, smooth with very sparse and shallow punctures; ventral margins of tergites 3 and following overlapping.

MALE (2 specimens). B 5.6–5.7; A 3.4–3.5; F 3.5–3.8. Antenna slenderer, not widened apically, without median pale ring, with 25 flagellomeres in the only specimen with complete antennae (Fli_1_ 1.9; Fli_15_ 1.0; Fli_24_ 1.0); ventral margins of tergites not overlapping; otherwise similar to female.

##### Etymology.

Named after the type locality. Noun in apposition.

##### Distribution.

Democratic Republic of Congo,Uganda, Zimbabwe.

##### Comments.

The two specimens from Kivu (MRAC) differ significantly from the type series: they are about half the size with shorter antennae; the mesopleuron is more smoothly sculptured, transversely strigose; and the OT index is 0.3. The coloration and sculpture are otherwise similar. Whether or not they actually belong to the same species is currently unclear. We refrain from delimiting these specimens as a separate species until further material is at hand to assess the degree of intra–specific variation within this taxon.

**Figure 7.**
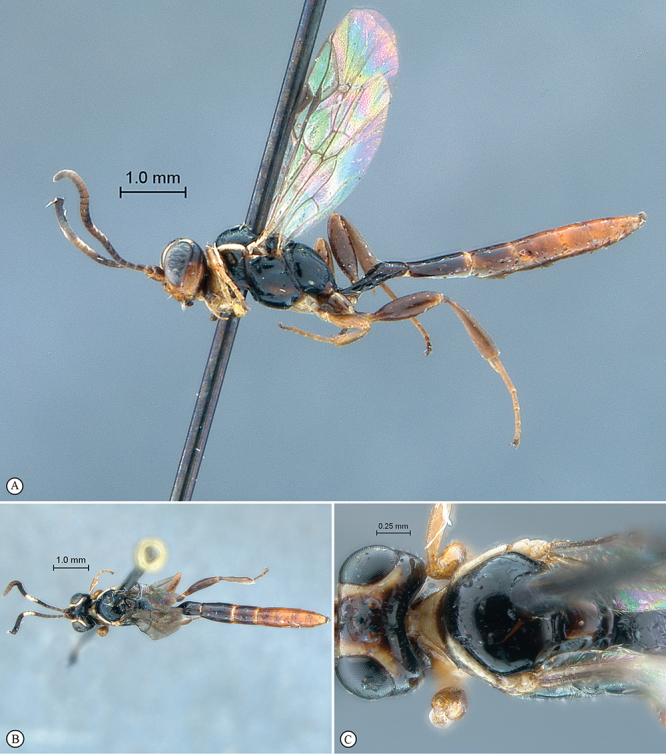
*Chauvinia nyanga* Holotype female. **A** habitus lateral view **B** habitus dorsal view **C** head, mesosoma, dorsal view.

**Figure 8.**
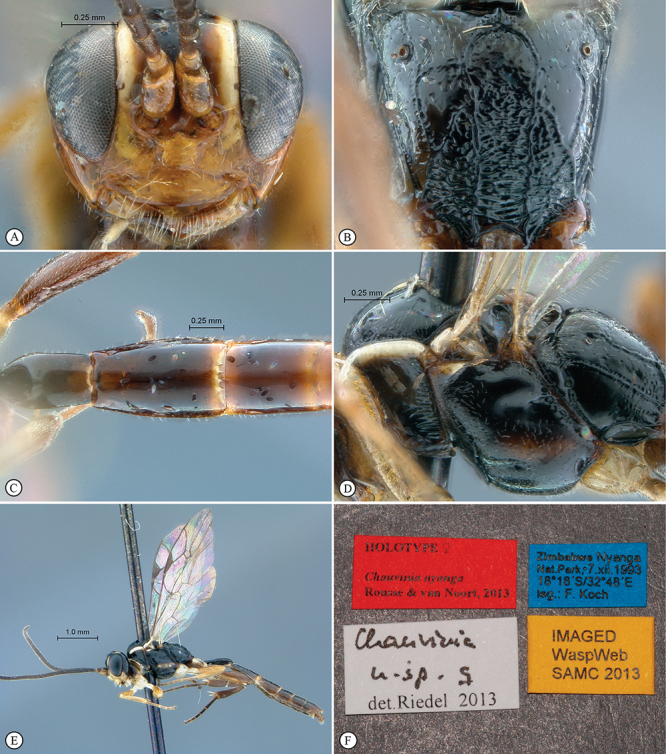
*Chauvinia nyanga* Holotype female (**A–D, F**), non–type male (**E**). **A** head, anterior view **B** propodeum dorsal view **C** tergites 1–3, dorsal view, **D** mesosoma, lateral view **E** male habitus lateral view **F** data labels.

#### Chauvinia pelecinoides

Heinrich, 1938

http://species-id.net/wiki/Chauvinia_pelecinoides

Figs 9 –10

##### Material examined.

**SYNTYPES 1 female:** Madagaskar, Rogez, 600 m, V–VI.1931 leg. A. Seyrig (ZMPA). 1male: same label data (ZMPA).

##### Diagnosis.

Female: head more or less extensively yellowish–orange ventrally, remainder of head pale yellow with vertex and occiput black; remainder of body yellowish–orange, apex of metasoma sometimes infuscate; antenna short with 22 flagellomeres, basally dark testaceous, medially white and apically dark brown; upper tooth of mandible twice as long as lower tooth; head and body very faintly sculptured, mostly smooth and polished; propodeum strongly extending apically between hind coxae to their half–length; propodeum mid–longitudinally deeply concave, carination moderate; metasoma strongly elongate, distinctly longer than hind leg; tergite 3 and following ventrally overlapping. B 11.7; A 4.2; F 5.2. HdWi 1.9; HfWi 1.1; Ci 2.8; Mi 0.7; Di 1.9; IOi 1.2; OOi 0.8; Fli_1_ 4.0; Fli_15_ 1.1; Fli_21_ 0.8; OTi 0.1. Male: head without yellowish–orange ventral coloration; metasoma dark brown, tergites 2–3 basally yellow, all metasomal tergites apically yellow margined; flagellum entirely dark brown, slenderer; metasoma hardly reaching beyond apex of hind tibia; otherwise similar to female. B 7.2; A 3.8; F 4.8.

##### Distribution.

Madagascar (Toamasina province) (Heinrich 1938).

**Figure 9.**
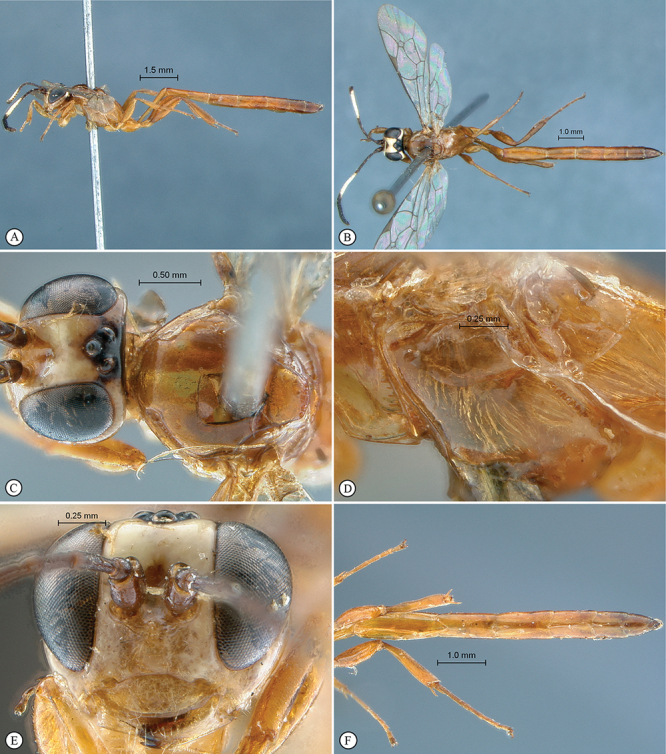
*Chauvinia pelecinoides* Holotype female. **A** habitus lateral view **B** habitus dorsal view **C** head, mesosoma, dorsal view **D** mesopleuron, lateral view **E** head, anterior view **F** metasoma, ventral view.

**Figure 10.**
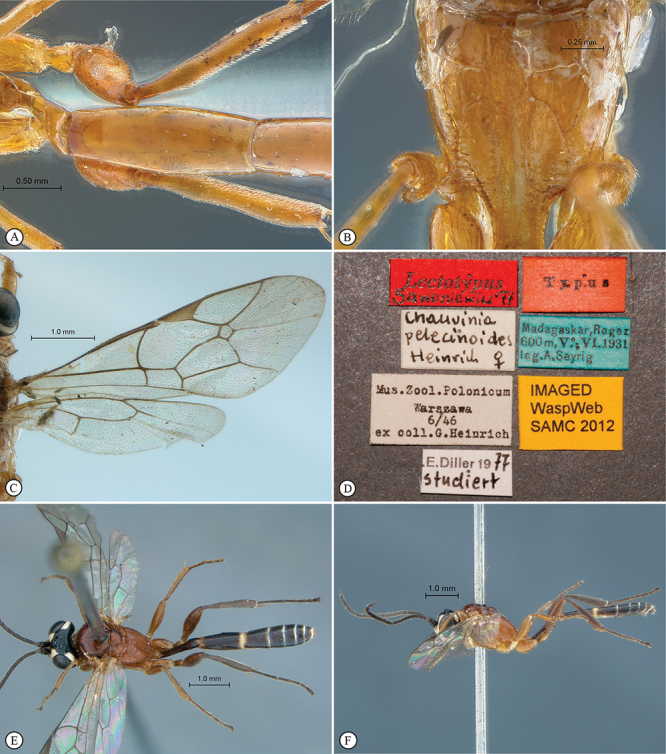
*Chauvinia pelecinoides* Holotype female (**A–D**), non–type male (**E–F**). **A** tergites 1–3, dorsal view **B** propodeum dorsal view **C** wings, **D** data labels **E** habitus dorsal view **F** habitus lateral view.

#### Diadromus (Thyraeella)

Wesmael, 1845

Thyraella Holmgren, 1890

##### Diagnosis.

Mandible bidentate, triangular and evenly narrowed towards apex, with a weak ventral flange, upper tooth twice as long as lower tooth; face transverse, wider than high; clypeus distinctly transverse, lenticular, hardly separated from face medially, its ventral margin somewhat impressed; occipital carina complete; occipital and hypostomal carinae joining at mandibular base; flagellum of female moderately enlarged and flattened beyond middle, flagellum of male without tyloids; epomia present and strong; mesoscutum steeply elevated above pronotum; postpectal carina complete and strong; propodeum moderately short, basal half about horizontal and apical half sloping down in lateral view; carination complete and strong, spiracle quite round; fore wing with areolet pentagonal, closed, 3Rs–m non–tubular and faintly pigmented; hind wing with distal abscissa of Cu1 faint; gastrocoeli large and moderately deep; tarsal claws simple; thyridiae moderately weak but distinct, transverse and wide with interval narrow, distant from anterior margin by more than their width; hypopygium hiding base of ovipositor sheath; ovipositor very shortly projecting beyond metasomal apex.

##### Species richness and distribution.

*Diadromus* is a moderately large genus of 31 species, occurs almost worldwide, but has not been reported from South America or tropical Africa. The subgenus *Thyraeella* is highly unusual among the other species of the genus: it is mainly defined by the peculiar position of the junction between the hypostomal and occipital carinae, the complete postpectal carina and by having the ventral margin of the clypeus less impressed than in *Diadromus* *s. s.* The subgenus only includes the species *Diadromus collaris*.

#### Diadromus collaris

(Gravenhorst, 1829)

http://species-id.net/wiki/Diadromus_collaris

Figs 11 –12

Diadromus cabrerai Berthoumieu, 1903; *Diadromus punicus* Berthoumieu, 1898; *Diadromus rufiscapus* Pic, 1902; *Heterischnus hispanicus* Berthoumieu, 1904; *Ischnus collaris* Gravenhorst, 1829; *Ischnopsidea brevicauda* Hellén, 1949; *Phaeogenes bellulus* Kriechbaumer, 1894; *Phaeogenes similis* Bridgman, 1881.

##### Material examined.

**South Africa** 1 female: South Africa Brits, Tvl. viii.X.1993 R. Kfir AcP 9336; ex pupae of *Plutella xylostella* on cabbage IIE 23016 (BMNH); 2 females: RSA [Republic of South Africa], S. DBerg, Sani Pass, 29°37'S, 29°23'E, Malaise trap, V. Kolyada and M. Mostovski coll., 2–5.03.04 (NMSA); 2 females: SAfr, KZN, PMB [Pietermaritzburg], Hilton, 29°32'30"S, 30°18'18"E, 1131m, 10–23.12.03, Malaise trap / garden M. Mostovski coll. (NMSA). **Sweden** 1 female: Sweden SK. Dalby. 13.v.1938. D. M.S. P. & J. F. P. B. M.1938–414 (BMNH).

##### Diagnosis.

Head black, body usually reddish–orange with apex of mesosoma, and base and apex of metasoma black; body sometimes far more extensively black marked; face, frons, vertex and temple shallowly and densely to moderately densely punctate; malar line long; antenna with 23–30 flagellomeres, slightly widened from basal third; mesosoma entirely polished and moderately setose; pronotum moderately punctate with a large median smooth area; mesopleuron densely punctate, speculum smooth; metapleuron coarsely punctate–rugose; mesonotum moderately punctate; scuto–scutellar groove smooth; scutellum carinate to mid–length; propodeum shallowly punctate rugose, area petiolaris concave, carination complete with area superomedia hexagonal, slightly wider than long; hind wing with distal abscissa of Cu1 discernible though faint; metasoma alutaceous but apical half of tergite 1 and base of tergite 2 longitudinally striate. B 4.5–5.3; A 3.1–3.2; F 3.3–3.7 (ranges measured on all observed material); HdWi 1.7; HfWi 1.2; Ci 1.8; Mi 0.9; Di 3.2; IOi 1.6; OOi 1.6; Fli_1_ 3.5; Fli_15_ 1.4; Fli_23_ 1.1; OTi 0.4 (indices measured on BMNH South African female specimen).

##### Distribution.

South Africa, Mexico. Otherwise widespread from Europe and the Middle East to the Indo–Australian region, China and Japan. Introduced into numerous countries in the Indo–Australian and West Indies regions for biocontrol purpose.

##### Ecology.

Commonly used as a biological control agent of *Plutella xylostella* (Lepidoptera: Plutellidae) on cultivated cruciferous crops (*Brassica* spp.). Also reared from *Acrolepiopis assectella* (Lepidoptera: Plutellidae) (Kalmès and Rojas–Rousse 1980). The geographical origin of this species is not clear, neither is that of its host *Plutella xylostella*, which was originally considered to be European. Kfir (1998), based on an assessment of the origins of the moth’s host plants and the complex of natural enemies attacking the diamondback moth, suggested that the host species is from southern Africa. *Diadromus collaris* is very common in South Africa, where it reproduces sexually (Kfir 1997, 1998), and in Europe, where it was reported to be thelytokous with mainly females known. It hence was speculated to be of African origin (Kfir 1998). However, Liu et al. (2001) report that *Diadromus collaris* is arrhenotokous in Australia, Malaysia, China, Taiwan and France, and suggested that the diamondback moth originates in China. *Diadromus collaris* is probably present throughout the Afrotropical region; however, it is not present in Reunion, where the diamondback moth is only a minor pest (Rousse pers. obs.).

**Figure 11.**
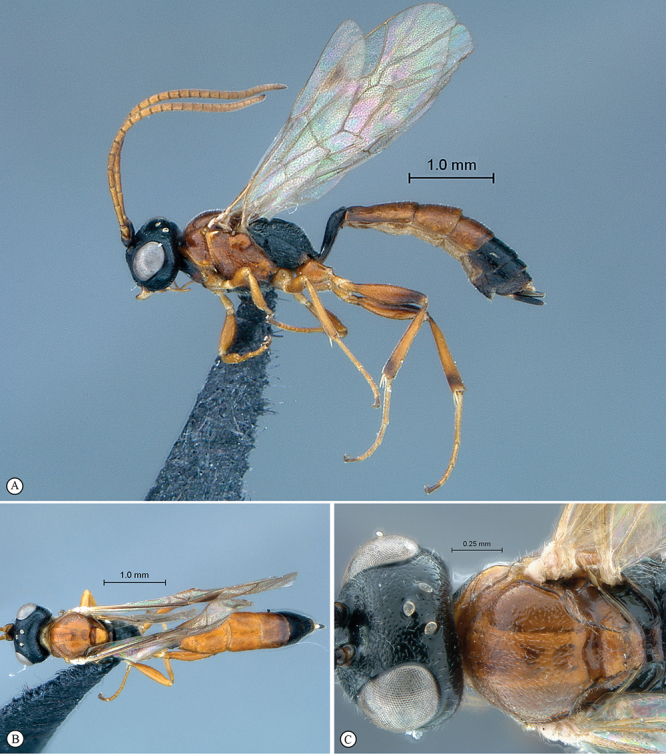
*Diadromus collaris* Holotype female. **A** habitus lateral view **B** habitus dorsal view **C** head, mesosoma, dorsal view.

**Figure 12.**
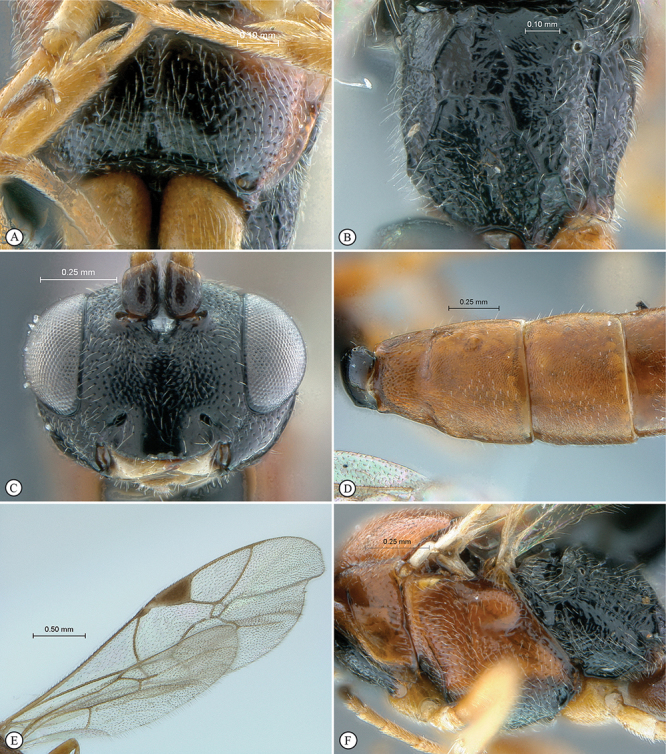
*Diadromus collaris* Holotype female. **A** metapleuron, ventral view **B** propodeum dorsal view **C** head, anterior view **D** tergites 2–4, dorsal view **E** wings **F** mesosoma, lateral view.

#### Dicaelotus

Wesmael, 1845

http://species-id.net/wiki/Dicaelotus

Cinxaelotus Holmgren, 1890; *Deloglyptus* Förster, 1869; *Euryptilus* Holmgren, 1890; *Holocreptis* Förster, 1869; *Leptodemas* Förster, 1869.

##### Diagnosis.

Mandible bidentate; occipital and hypostomal carinae joining close to or distinctly above mandibular base; area superomedia defined, receiving costula at or beyond middle; gastrocoelus indistinct, thyridium absent; fore wing with areolet closed; hind wing with distal abscissa of Cu1 present, hardly distinct to fully pigmented (Perkins 1959, Selfa and Diller 1994).

##### Species richness and distribution.

The genus is represented by 57 species, with a worldwide distribution with the exception of the Australasian and Antarctic regions. *Dicaelotus cariniscutis* (Cameron, 1906) was the only Afrotropical species previously known. We describe here three new species from South Africa.

#### Dicaelotus asantesana

Rousse & van Noort sp. n.

http://zoobank.org/5702DCF4-D6EB-49DB-880C-A7403203AD0C

http://species-id.net/wiki/Dicaelotus_asantesana

Figs 13 –14

##### Type material.

**HOLOTYPE** Female:South Africa, Eastern Cape, Asante Sana Game Reserve, 32°14.930'S, 24°56.975'E, 1642m, 6.x.2010–17.i.2011, S. van Noort, Malaise trap, Southern Karoo Riviere *Leucosidea* dominated, ASA09–OUB1–M16, SAM–HYM–P047364 (SAMC). **PARATYPES** 10 females: same label data as holotype; 4 females and 5 males: same data as holotype except: 32°14.990'S, 24°55.962'E, 2183m, 29.x.2009–23.ii.2010, Karoo Escarpment Grassland, ASA09–GRA1–M02, SAM–HYM–P047365 (BMNH, SAMC); 1 female same data as holotype except: 32°16.762'S, 24°57.309'E, 1186m, 7 Apr–28 July 2010, Southern Karoo Riviere Riverine Woodland, ASA09–WOO1–M10, SAM–HYM–P047366 (SAMC); 1 female: South Africa, Western Cape, Gamkaberg Nature Reserve, 33°39.504'S, 21°53.947'E, 322m, 5–23 May 2009, S. van Noort, Malaise trap, Gamka Thicket, GB09–SUC04–M09, SAM–HYM–P047367 (SAMC).

##### Diagnosis.

Mostly black species with yellowish to pale yellow markings on head, mesosoma and legs (pale coloration more extensive in males); entire head densely setose; antenna short and stout with 22 flagellomeres, flagellomeres quadrate to shorter than wide in female, slightly longer in male; ocelli reduced, inter–ocellar distance twice as long as ocellar diameter; mesosoma of female distinctly depressed, more than twice as long as high in profile; hind wing with distal abscissa of Cu1 weak; metasomal tergites deeply and regularly punctate–reticulate. HdWi 2.0; HfWi 1.3; Mi 1.0; Ci 2.6; Di 5.0; IOi 2.1; OOi 1.1; Fli_1_ 1.0, Fli_15_ 0.8; Fli_21_ 0.8; OTi 0.3.

##### Description.

FEMALE (17 specimens). B 3.3–4.4; A 1.4–1.6; F 2.1–2.8 (Holotype B 3.9; A 1.5; F 2.5).

*Color*. Head black with middle of face sometimes dark reddish, clypeus and mandible more or less extensively yellow, facial orbits often pale marked, palpi pale yellow, and antenna blackish brown; mesosoma black with upper margin of pronotum sometimes pale yellow; metasoma black fading apically to dark brown, apical margin of tergite 2 and following pale yellow; legs dark testaceous with fore and mid coxae and all trochanters more or less extensively yellow; wings hyaline, venation light brown.

*Head*. Entirely densely setose, including eyes, setae long; face medially bulging, deeply and coarsely punctate–reticulate, punctation finer medially; clypeus dorsally punctate, almost smooth ventrally, strongly transverse, distinctly convex in profile, its ventral margin regularly convex; mandible slender, elongate, lower tooth strongly reduced; malar line long, subocular sulcus present as a granulate area; hypostomal carina joining occipital carina shortly but distinctly above mandibular base; frons, vertex and temple densely and regularly punctate; ocellar triangle wide, wider than long, ocelli reduced; antenna unusually short and stout, apically pointed, with 22 flagellomeres, all but the first ones shorter than wide.

*Mesosoma*. Distinctly depressed, twice longer than high in profile view, polished, regularly, deeply and densely punctate if not otherwise specified; most of pronotum longitudinally striate, epomia moderate; speculum entirely punctate, punctation somewhat finer than on remainder of mesopleuron, sternaulus deep on anterior third of mesopleuron; mesonotum rather flat with finer punctation, notaulus indistinct; area petiolaris slightly concave with punctation shallower and coarser, carination complete, area superomedia heart–shaped, receiving costula a little posterior to middle. *Wings*. Hind wing with distal abscissa of Cu1 weak, emitted by 1/Cu&cu–a at its posterior fifth.

*Metasoma*. All tergites deeply and regularly punctate–reticulate.

MALE (5 specimens). B 2.8–4.3; A 2.0–2.3; F 2.0–2.9. Pale coloration more extensive; pale yellow: inner orbits, face, clypeus, mandible, palpi, anterior and dorsal margin of pronotum, tegula, antero–dorsal corner of mesopleuron; mesopleuron often with a faint, hardly delimited mid–longitudinal pale stripe; legs light testaceous with coxae and trochanters pale yellow but hind coxa with large black markings, flagellum entirely testaceous ventrally; antenna slenderer (Fli_1_ = Fli_15_ = Fli_21_ = 1.4); pilosity on head sparser and shorter; mesosoma stouter, less than twice as long as high in profile; otherwise similar to female.

##### Etymology.

Named after the type locality. Asante Sana is Swahili for “thank you very much”. Noun in apposition.

##### Distribution.

South Africa (Eastern and Western Cape).

**Figure 13.**
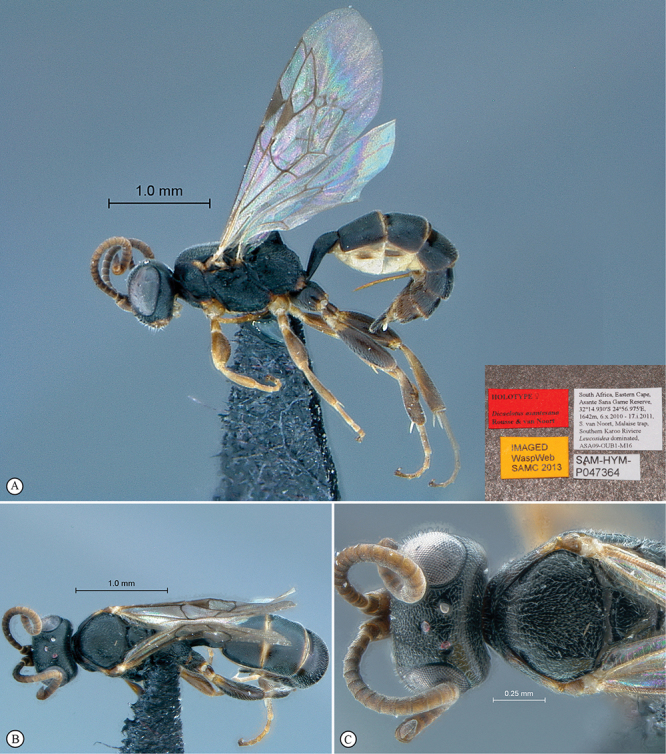
*Dicaelotus asantesana* Holotype female. **A** habitus lateral view (inset, data labels) **B** habitus dorsal view **C** head, mesosoma, dorsal view.

**Figure 14.**
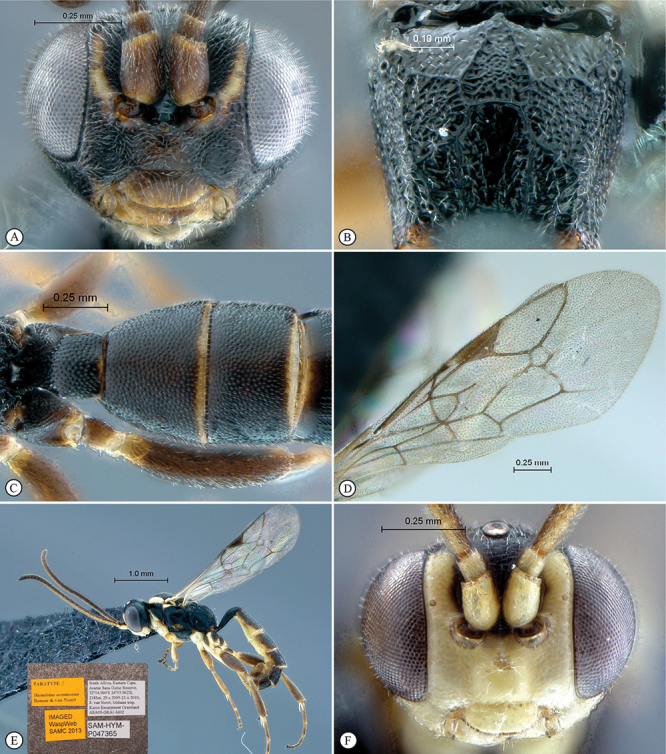
*Dicaelotus asantesana* Holotype female (**A–D**), Paratype male (**E–F**). **A** head, anterior view **B** propodeum dorsal view **C** tergites 1–4, dorsal view **D** wings **E** habitus, lateral view (inset, data labels) **F** head, anterior view.

#### Dicaelotus cariniscutis

(Cameron, 1906)

http://species-id.net/wiki/Dicaelotus_cariniscutis

Figs 15 –16

Leptodemas cariniscutis Cameron, 1906: 330.

##### Material examined.

**HOLOTYPE.** Male: Cape P. [South Africa] Cameron Coll. 1914–110, B. M. Type Hym 3.G.356 (BMNH).

##### Diagnosis

(female unknown). Head black with face, clypeus and mandible pale yellow, mesosoma black, metasoma mostly reddish to brown with tergite 1 almost black; temple straight in dorsal view; head finely punctate to coarsely punctate–reticulate; mesosoma densely and deeply punctate but area petiolaris transversely strigose; propodeum short, its posterior half almost vertical; propodeal carination strong and complete; area petiolaris strongly concave; hind wing with distal abscissa of Cu1 very faint, reaching wing margin; metasomal tergites deeply and regularly punctate; B 4.8; A NA; F 3.3; HdWi 2.1; HfWi 1.2; Mi NA; Ci NA; Di NA; IOi 1.3; OOi 1.0; Fli_1_ NA; Fli_15_ NA; Fli_21_ NA; OTi NA. Male unknown.

##### Comments.

The holotype of *Dicaelotus cariniscutis* is in relatively poor condition, with the antennae broken and legs either broken or covered in glue. The ventral third of the head is also hidden by glue, making most of the relative measurements impossible.

##### Distribution.

South Africa (Cape Province).

**Figure 15.**
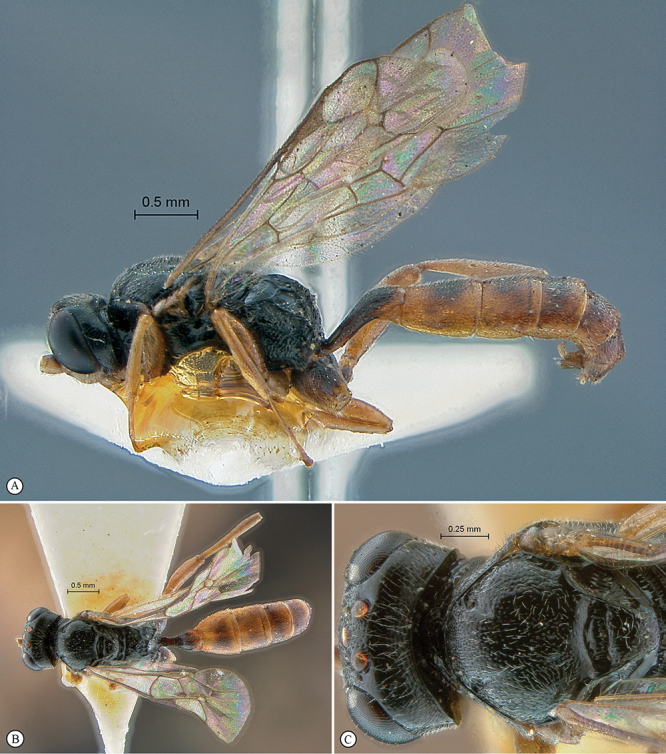
*Dicaelotus cariniscutis* Holotype female. **A** habitus lateral view **B** habitus dorsal view **C** head, mesosoma, dorsal view.

**Figure 16.**
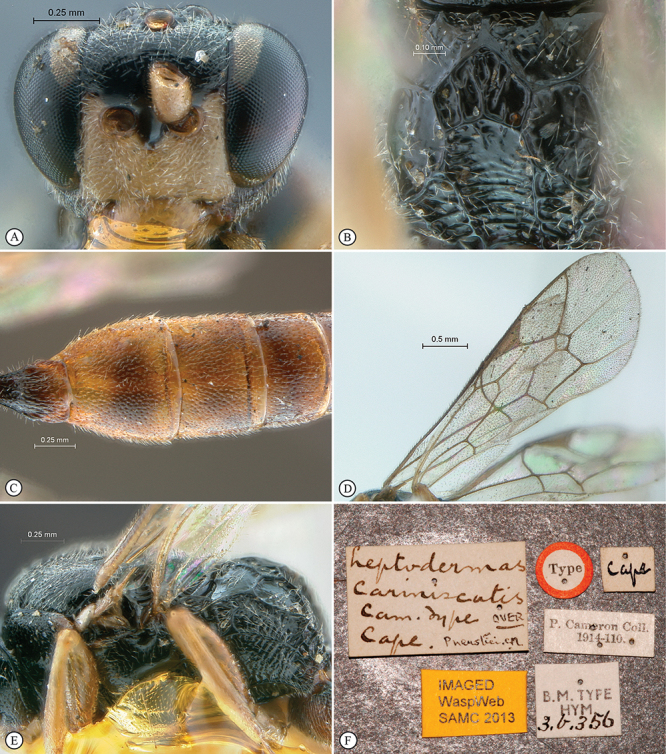
*Dicaelotus cariniscutis* Holotype female. **A** head, anterior view **B** propodeum dorsal view **C** tergites 1–4, dorsal view **D** wings **E** mesosoma, lateral view **F** data labels.

#### Dicaelotus hoerikwaggoensis

Rousse & van Noort sp. n.

http://zoobank.org/01318595-47F2-4470-95A6-7669B1144DDA

http://species-id.net/wiki/Dicaelotus_hoerikwaggoensis

Figs 17 –18

##### Type material.

**HOLOTYPE.** Female: South Africa, W. Cape, Cape Town, abov. Tokai Forest, Constantiaberge, above Donkerboskloof, 460 m altitude, 34°02'S, 18°23.5'E, 9–15 Feb 1994, S. van Noort, mesic mountain fynbos on sandstone, *Protea* dominated. Malaise trap, SAM–HYM–P005586 (SAMC). **PARATYPE** 1 Female: Natal, Van Reenen, Drakensberg 1–22.i.1927, S. Africa, R.E. Turner, Brit. Mus. 1927–54 (BMNH).

##### Diagnosis.

Mostly black species interspersed with numerous brown and yellow maculae; entire body shining; head sparsely to moderately densely punctate; face distinctly protruding medially; antenna short and stout; mesosoma coarsely punctate or strigose laterally, more finely punctate dorsally; mesoscutum without differentiated lobes; propodeal carination strong and complete; hind wing with distal abscissa of Cu1 present but non–pigmented; metasomal tergites sparsely punctate to almost smooth. HdWi 2.6; HfWi 1.2; Mi 0.6; Ci 2.4; Di 2.5; IOi 1.8; OOi 1.2; Fli_1_ 1.6; Fli_15_ 1.2; Fli_24_ 1.0; OTi 0.4. Male unknown.

##### Description.

FEMALE (2 specimens). B 4.8–5.1; A 3.0–3.2; F 3.0–3.4 (Holotype B 5.1; A 3.2; F 3.4).

*Color*. Head yellow with black and brown parts; black: frons, vertex but two small triangles antero–posteriorly, occiput, temple and genae; brown: antenna and face around median protuberance; mesosoma mainly black with a yellow longitudinal stripe on lateral part of pronotum, another one on mesopleuron, propleuron and ventral part of pronotum and mesopleuron fading to reddish; wings hyaline, venation light brown; fore and middle legs testaceous with coxae and trochanters yellow, hind leg brownish with coxa and trochanter largely tinged with yellow; metasomal tergite 1 black, the following blackish brown and apically yellow.

*Head*. Transverse in dorsal view, shining; mandible sparsely punctate, moderately long, regularly narrowed to apex; malar line long, subocular sulcus present as a wide and shagreened groove; clypeus sparsely punctate, transverse, lenticular; face transverse, moderately densely punctate, medially protruding into a blunt square tubercle connected to antennal socket by a short and faint longitudinal carina; frons and vertex finely and sparsely punctate; ocellar triangle about equilateral; hypostomal carina joining occipital carina distinctly above mandibular base; antenna stout and short, flagellum not enlarged nor flattened, with 24–25 flagellomeres.

*Mesosoma*. Entirely shining; pronotum longitudinally strigose, except upper third moderately punctate and antero–ventral corner smooth; mesopleuron densely punctate, longitudinally strigose postero–dorsally, speculum smooth; sternaulus deep and crenulate at anterior third; epicnemial carina ventrally moderately expanded between fore coxae; postpectal carina ventrally absent; metapleuron transversally strigose, dorsally smooth; mesonotum finely and moderately punctate, notaulus hardly visible near base; scutellum flat, not carinate; propodeum punctate–rugose but area superomedia centrally smooth and area petiolaris transversely striate, carination complete and strong, area superomedia heart–shaped. *Wings*. Hind wing with distal abscissa of Cu1 present, non–pigmented. *Legs*. Stout and densely punctate; hind femur and hind tibia somewhat swollen.

*Metasoma*. Shining; tergites 1–2 sparsely punctate, following tergites almost smooth with isolated fine punctures; ovipositor straight and moderately long.

MALE. Unknown.

##### Etymology.

Named in honour of the Table Mountain National Park, the conservation area encompassing the type locality. The original inhabitants of the Cape, the KhoiKhoi, called the Table Mountain *Hoerikwaggo* meaning”sea mountain” or “mountain in the sea”.

##### Distribution.

South Africa (Kwazulu–Natal and Western Cape).

##### Discussion.

This species and the following one are sympatric and closely related. They share the same microsculpture, particularly the coarsely punctate and strigose mesosoma and the blunt median tubercle on the face. They are, however, distinct, and can be differentiated by the length of antennae, the pigmentation of the distal abscissa of Cu1 on the hind wing and the strikingly distinct coloration pattern.

**Figure 17.**
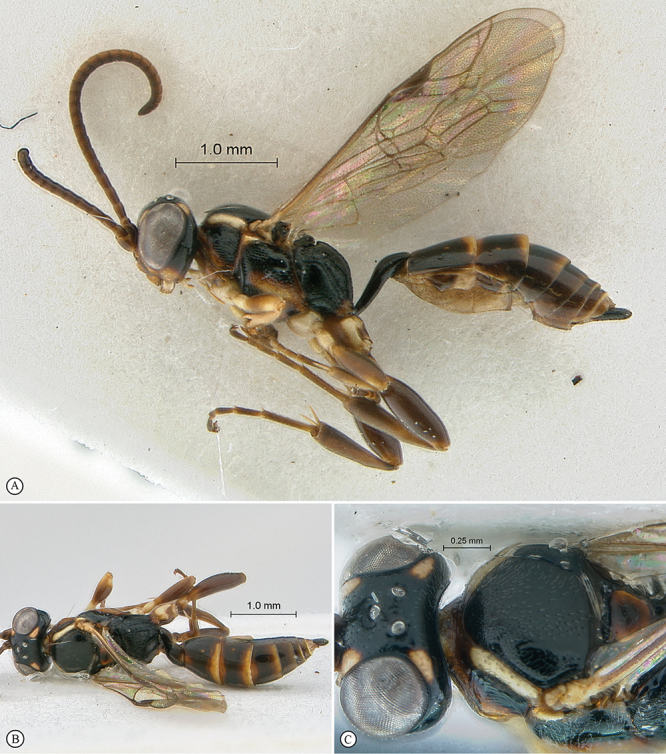
*Dicaelotus hoerikwaggoensis* Holotype female. **A** habitus lateral view **B** habitus dorsal view **C** head, mesosoma, dorsal view.

**Figure 18.**
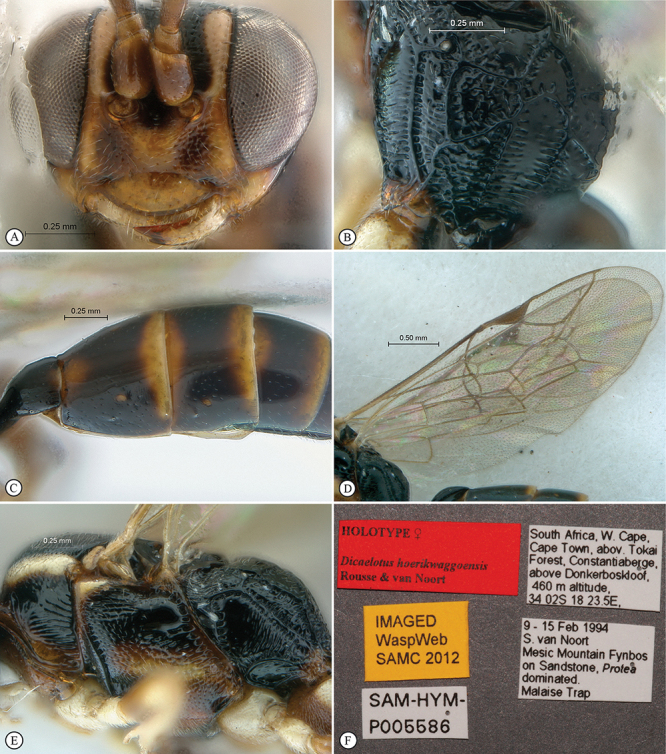
*Dicaelotus hoerikwaggoensis* Holotype female. **A** head, anterior view **B** propodeum dorsal view **C** tergites 1–4, dorso–lateral view **D** wings **E** mesosoma, lateral view **F** data labels.

#### Dicaelotus tablemountainensis

Rousse & van Noort sp. n.

http://zoobank.org/0353AB41-5B61-4476-8A94-3549B888E8E3

http://species-id.net/wiki/Dicaelotus_tablemountainensis

Figs 19 –20

##### Type material.

**HOLOTYPE.** Female: South Africa, W. Cape, Cape Town, abov. Tokai Forest, Constantiaberge, above Donkerboskloof, 460 m altitude, 34 02S 18 23.5E, 9–15 Feb 1994, S. van Noort, mesic mountain fynbos on sandstone, *Protea coronata* dominated, sweep SAM–HYM–P006392 (SAMC). **PARATYPE** 1 female: Cape Town, Table Mt., K. Barnard, Feb. 1919, SAM–HYM–P005531 (SAMC).

##### Diagnosis.

Mostly reddish interspersed with black and yellow markings; entire body shining; head sparsely to densely punctate; face distinctly protruding medially; mesosoma coarsely punctate or strigose laterally, more finely punctate dorsally; mesoscutum without differentiated lobes; hind wing with distal abscissa of Cu1 fully pigmented; propodeal carination complete but costula faint; metasomal tergites 1–3 densely and shallowly punctate, following tergites alutaceous. HdWi 2.4; HfWi 1.1; Ci 2.3; Mi 0.8; Di 1.3; IOi 1.8; OOi 1.2; Fli_1_ 1.8; Fli_15_ 1.0; Fli_28_ 0.8; OTi 0.3. Male unknown.

##### Description.

FEMALE (2 specimens). B 5.8–6.1; A 3. 6–3.7; F 3.7–3.9 (Holotype B 5.8; A 3.6; F 3.7).

*Color*. Head black with eye margin (except on temple) yellow, clypeus, mandible and palpi reddish, and flagellum tri–colored: basal half dark brown, apical half testaceous, flagellomeres 9–12 white; mesosoma reddish with a yellow longitudinal stripe on lateral part of pronotum, and black parts: remaining of pronotum, subtegular ridge and axillar furrows around scutellum and post–scutellum; wings hyaline, venation light brown; legs reddish with hind tibia and hind coxa largely infuscate; metasoma reddish with tergites 6–7 and extreme base of tergite 1 blackish.

*Head*. Shining; face transverse, densely punctate, medially protruding into a blunt square tubercle connected to antennal socket by a short transverse carina; clypeus lenticular, transverse, sparsely punctate; malar line moderately long, subocular sulcus present as a granulate groove; mandibles sparsely punctate, moderately long, regularly narrowed toward apex; frons, vertex and temple finely and sparsely punctate; ocellar triangle wider than high; antenna stout with 28–29 flagellomeres.

*Mesosoma*. Entirely shining; pronotum longitudinally strigose but upper third moderately punctate; mesopleuron densely punctate but posterior margin costulate, speculum smooth, sternaulus deep at anterior third; epicnemial carina ventrally moderate; postpectal carina ventrally absent; metapleuron transversely strigose–punctate, dorsally smooth; mesonotum finely and moderately punctate, notaulus hardly visible near base; scutellum flat, not carinate; propodeum punctate–rugose but area petiolaris transversally striate, carination complete and strong except costula partly obsolete, area superomediaelongate, pentagonal. *Wings*. Hind wing with distal abscissa of Cu1 fully pigmented, connected to 1/Cu&cu–a, cu–a nearly 3× shorter than 1/Cu. *Legs*. Stout and densely punctate; hind femur and hind tibia somewhat swollen.

*Metasoma*. Shining; metasomal tergites 1–3 densely and shallowly punctate, following tergites alutaceous; ovipositor straight and moderately long.

MALE. Unknown.

##### Etymology.

Named in honour of the Table Mountain National Park, to which the type locality belongs. In 1503, a Portuguese explorer, Antonio de Saldanha, climbed and named the mountain *Taboa do Cabo* (= Table of the Cape). Subsequently the mountain became known as Tafelberg (= Table Mountain) to the first European Dutch settlers of the Cape who arrived in 1652.

##### Distribution.

South Africa (Western Cape).

##### Discussion.

*cf.* *Dicaelotus hoerikwaggoensis* Rousse & van Noort, sp. n.

**Figure 19.**
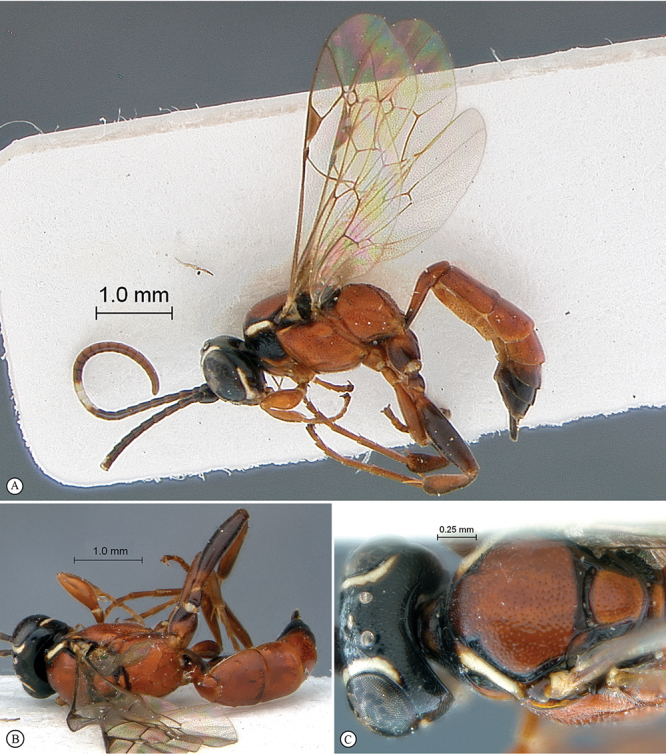
*Dicaelotus tablemountainensis* Holotype female. **A** habitus lateral view **B** habitus dorsal view **C** head, mesosoma, dorsal view.

**Figure 20.**
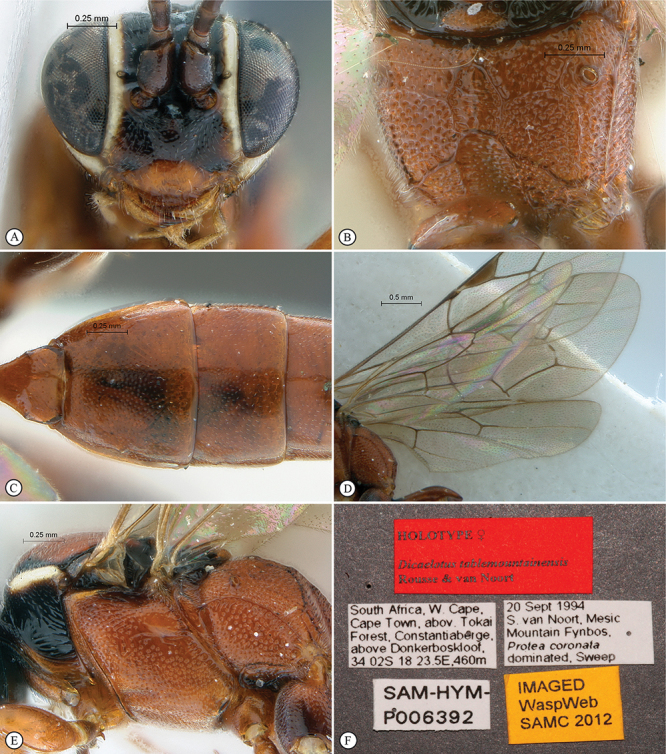
*Dicaelotus tablemountainensis* Holotype female. **A** head, anterior view **B** propodeum dorsal view **C** tergites 1–4, dorsal view **D** wings **E** mesosoma, lateral view **F** data labels.

#### Heterischnus

Wesmael, 1859

http://species-id.net/wiki/Heterischnus

Aethiopischnus Heinrich, 1938; *Ischnopsidea* Viereck, 1914; *Posocentrus* Provancher, 1875; *Rhexidermus* Förster, 1869.

##### Diagnosis.

*Heterischnus* is a very distinct genus of Phaeogenini and can be separated from other genera in the tribe by the combination of the following characters: mandible unidentate and falciform; clypeus separated from face laterally by a deep groove encompassing anterior tentorial pits; basal flagellar segments slender and long; vertex long and slightly convex behind ocelli; hypostomal carina joining occipital carina distinctly at or above mandibular base; epicnemial carina strongly raised ventrally and flexed over the base of the fore coxae; notaulus distinct anteriorly, deep and long; fore wing with areolet closed, hind wing with distal abscissa of Cu1 present and connected to 1/Cu&cu–a; tarsal claws simple; gastrocoelus nearly indistinct to deep, thyridiae wide; ovipositor extending relatively strongly beyond apex of metasoma (Perkins 1959, Selfa and Diller 1994).

##### Species richness and distribution.

The genus is represented by 28 species in the Afrotropical, Nearctic and Palearctic regions, with three described Afrotropical species. We add here one species from Southern Africa and one from Tanzania.

#### Heterischnus africanus

(Heinrich, 1936)

http://species-id.net/wiki/Heterischnus_africanus

Figs 21 –22

Aethiopischnus africanus Heinrich, 1936: 244.

##### Material examined.

**HOLOTYPE.** Female: [Kenya] Afrique Or. anglaise, M^t^ Kenya vers^t^ ouest zone inférieure, Alluaud & Jeannel, Riv. Burgurett, vallée boisée, 2200^m^, Janv 1912 St38 (MNHN EY8813). **PARATYPE.** Male: same label data (MNHN EY8814). **Other material**: **South Africa:** 1 male: Safr, KZN, PMB, Hilton 10–23.12.03 Malaise / Garden M. Mostovski coll. (NMSA); **Tanzania:** Tanganyika Terr.: Ngorongoro, Rest Camp, 2400–2500m, 6/19–VI–1957, Misssion Zoolog. I.R.S.A.C. en Afrique Orientale (P. Basilewsky et N. Leleup) (MRAC); **Uganda**: 1 male Rwenzori Mts. nr. Nyakalengija village along Mubuku river, ~1640 m 12.III.2013 A. Gumovsky; agricultural landscapes with cultivated plants + some ruderal plants, SAM–HYM–P047382 (SAMC).

##### Diagnosis.

Head black; mesosoma tri–colored black, yellow and testaceous; metasoma mostly testaceous with T1 partially black; legs yellow to light testaceous; head mostly punctate but face transversely puncto–striate; clypeus bluntly produced apico–laterally; hypostomal carina joining occipital carina distinctly above mandibular base; antenna with 30 flagellomeres; mesopleuron and metapleuron coarsely punctate with speculum smooth, remaining of mesosoma striate; propodeal carination reduced to apical transverse carina; metasoma densely punctate with T1 longitudinally puncto–striate; gastrocoelus and thyridium deep, inter–thyridia interval narrow. B 6.3; A 5.4; F 4.3; HdWi 2.1; HfWi 1.3; Ci 2.2; Mi 1.2; IOi 2.7; OOi 3.2; Fli_1_ 5.2; Fli_15_ 1.5; Fli_30_ 1.0; OTi 0.4. Male: similar to female but flagellum slenderer with 31 flagellomeres, sometimes with black spots on mesosoma greatly reduced; B 5.4; A 4.6; F 3.7.

##### Distribution.

Ethiopia, Kenya. South Africa, Tanzania, Uganda (new records).

**Figure 21.**
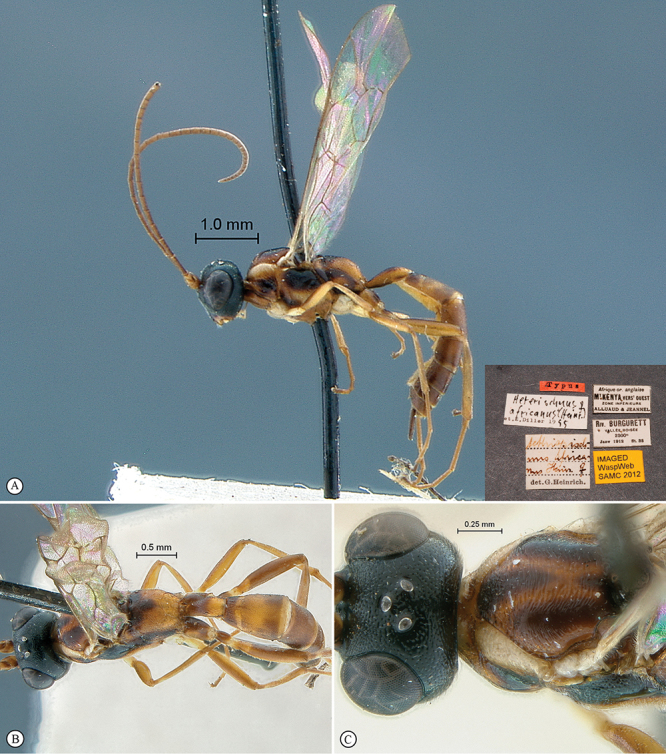
*Heterischnus africanus* Holotype female. **A** habitus lateral view (inset, data labels) **B** habitus dorsal view **C** head, mesosoma, dorsal view.

**Figure 22.**
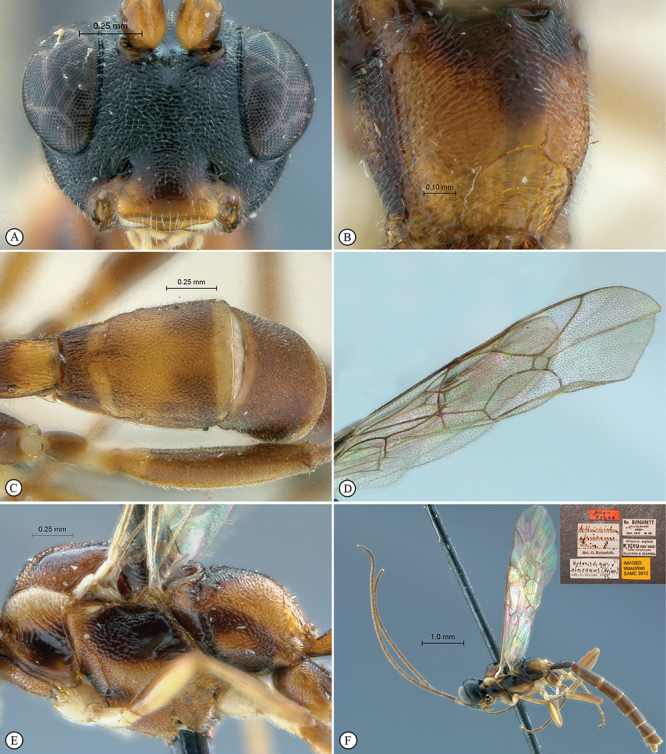
*Heterischnus africanus* Holotype female (**A–E**), Allotype male (**F**). **A** head, anterior view **B** propodeum dorsal view **C** tergites 1–3, dorsal view **D** wings **E** mesosoma, lateral view **F** habitus, lateral view (inset, data labels).

#### Heterischnus krausi

Schönitzer, 1999

http://species-id.net/wiki/Heterischnus_krausi

Figs 23 –24

##### Material examined.

**HOLOTYPE.** Female: Kenya–Limuru Katamayu–River 19.2.1948 (ZSMC). **Other material**: 1 female: Ruanda [Rwanda]: Gite de Nkuli 17.III.36 L. Lippens 26 (MRAC).

##### Diagnosis.

Mostly reddish–testaceous with yellow parts on head, mesosoma and coxae; head finely and very densely punctate, punctures somewhat arranged into transverse striations on face, into oblique striations on clypeus, frons and vertex transversely striate; clypeus apico–laterally sharply pointed; hypostomal carina joining occipital carina distinctly above mandibular base; antenna with 29 flagellomeres; mesosoma and metasoma densely and coarsely punctate but speculum and scutellum smooth, and most of pronotum, antero–dorsal corner of mesopleuron, most of mesoscutum, propodeum and most of tergite 1 striate; propodeum with a faint lateral longitudinal carina; gastrocoelus and thyridium deep, inter–thyridiae interval narrow; B 6.3; A NA; F 4.9. HdWi 1.6; HfWi 1.1; Ci 2.0; Mi 1.0; IOi 1.0; OOi 2.0; Fli_1_ 5.0; Fli_15_ NA; Fli_30_ NA; OTi 0. 3. Male unknown.

##### Distribution.

Kenya. Rwanda (new record).

**Figure 23.**
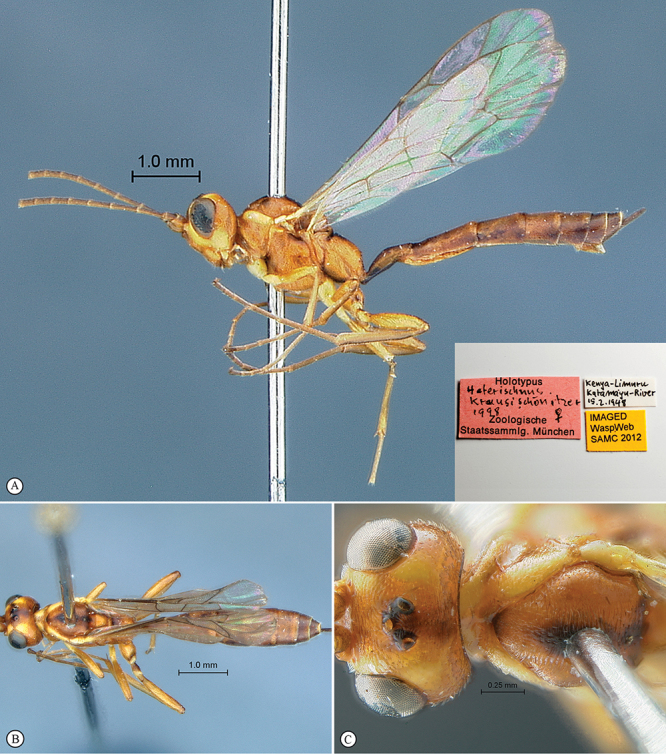
*Heterischnus krausi* Holotype female. **A** habitus lateral view (inset, data labels) **B** habitus dorsal view **C** head, mesosoma, dorsal view.

**Figure 24.**
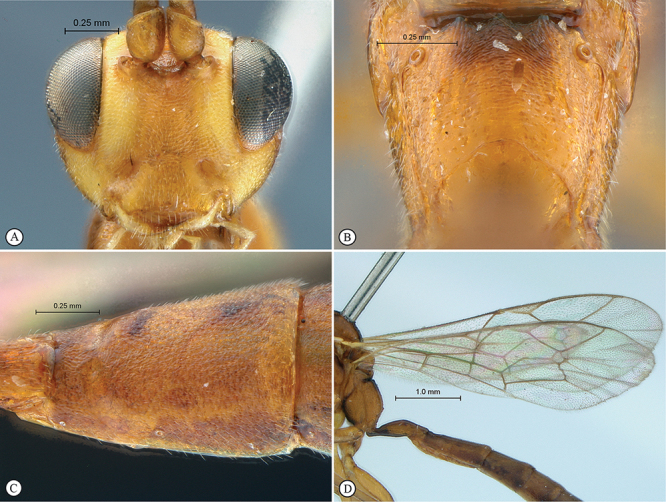
*Heterischnus krausi* Holotype female. **A** head, anterior view **B** propodeum dorsal view **C** tergite 2, dorsal view **D** wings.

#### Heterischnus mfongosi

Rousse & van Noort sp. n.

http://zoobank.org/51593E16-8562-4B0D-AC0E-B0151B44691F

http://species-id.net/wiki/Heterischnus_mfongosi

Figs 25 –26

##### Type material.

**HOLOTYPE.** Female: South Africa, Zululand, Mfongosi, Apr–May 1934, WE Jones, SAM–HYM–P001720 (SAMC). **PARATYPES.** 1 female: R.S.A. [Republic of South Africa] Karoo. Nat Park, 14.xi.1993 32°19'S, 22°30'E leg.: F. Koch (MNHU). 1 female: Zimbabwe: Salisbury [Harare] Chishawasha iii.1981. A. Watsham (BMNH); 1 female: same data as previous specimen, except: v.1981 (BMNH).

##### Diagnosis.

Head mostly black, mesosoma mostly yellowish orange, metasoma testaceous; antenna with a pale median ring; most of microsculpture densely punctate to punctate–reticulate but mesoscutum transversely striate; clypeus apico–laterally bluntly pointed; hypostomal carina joining occipital carina shortly but distinctly above mandibular base; propodeum without carination; gastrocoelus and thyridium deep, inter–thyridiae interval narrow. HdWi 1.7; HfWi 1.1; Ci 1.6; Mi 0.8; IOi 1.5; OOi 1.5; Fli_1_5.8; Fli_15_1.3; Fli_30_1.0; OTi 0.3. Male unknown.

##### Description.

FEMALE (4 specimens). B 7.5–7.6; A 5.5–5.7; F 4.7–4.9 (Holotype B 7.6; A 5.6; F 4.8).

*Color*. Head black with lower face and clypeus tending to dark red, mouthparts dark yellowish, scape and pedicel pale yellow, flagellum fuscous with basal flagellomeres lighter and flagellomeres 7–10 white; mesosoma yellowish–orange with mesoscutum somewhat darker and dorsal margin of pronotum yellow; legs yellowish–orange with all trochanters, fore and middle coxae pale yellow; wings hyaline, venation light brown; metasomal tergites testaceous with median black maculae of variable extent on tergites 3–5.

*Head*. Hemispherical, entirely densely and coarsely punctate to puncto–striate; mandible regularly tapered toward apex; malar line moderately long; clypeus triangular, its apical margin convex with two strong blunt teeth apico–laterally; face slightly transverse with a very weak median tubercle between toruli; ocellar triangle distinctly wider than high; temple long, regularly rounded behind eyes; hypostomal carina joining occipital carina distinctly above mandibular base; antenna hardly enlarged medio–apically, with 30–31 flagellomeres.

*Mesosoma*. Shining; pronotum rugulose, epomia weak; mesopleuron and metapleuron densely punctate–reticulate but speculum smooth; sternaulus moderate, reaching mid–length of mesopleuron; epicnemial carina ventrally moderately raised, slightly kinked medio–ventrally; postpectal carina interrupted in front of mid coxae; mesonotum transversely striate; notaulus anteriorly moderate, posteriorly obsolete; scutellum weakly convex, carinate to apical quarter; propodeum without carination, transversely aciculo–rugose, sculpture anteriorly weaker. *Wings*. Hind wing with distal abscissa of Cu1 joining 1/Cu&cu–a below its middle.

*Metasoma*. All tergites punctate–reticulate; tergite 1 moderately slender, regularly widened to apex; gastrocoelus within basal 1/5, wide and deep, thyridium transverse, inter–thyridiae interval very narrow; ovipositor straight.

MALE. Unknown.

##### Etymology.

Named after the type locality. Noun in apposition.

##### Distribution.

South Africa (Kwazulu–Natal, Western Cape), Zimbabwe.

**Figure 25.**
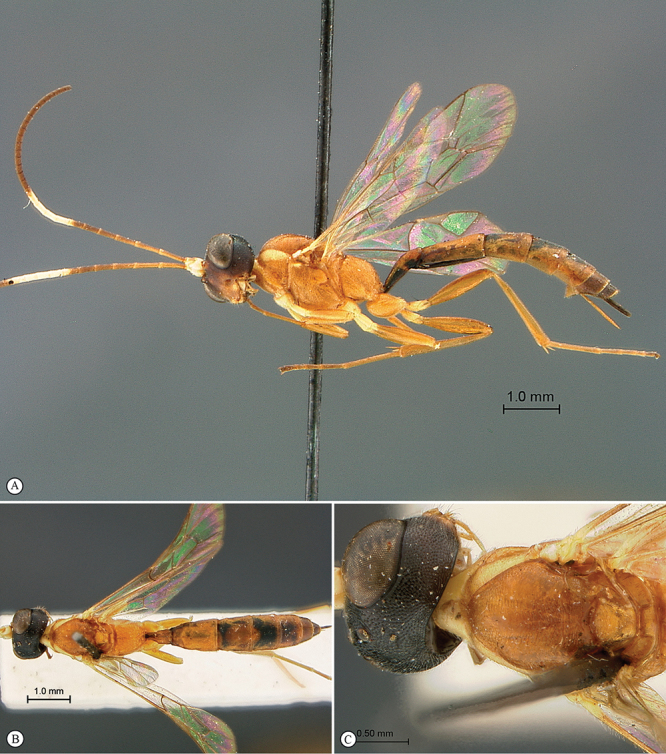
*Heterischnus mfongosi* Holotype female. **A** habitus lateral view **B** habitus dorsal view **C** head, mesosoma, dorsal view.

**Figure 26.**
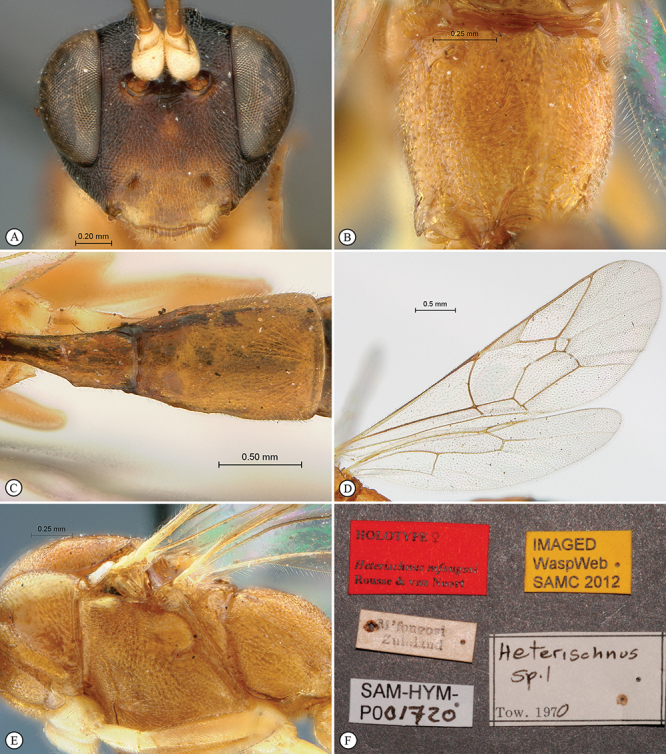
*Heterischnus mfongosi* Holotype female. **A** head, anterior view **B** propodeum dorsal view **C** tergites 1–2, dorsal view **D** wings **E** mesosoma, lateral view **F** data labels.

#### Heterischnus mkomazi

Rousse & van Noort sp. n.

http://zoobank.org/31C6B34A-5E84-4271-80B8-72F0A4316852

http://species-id.net/wiki/Heterischnus_mkomazi

Figs 27 –28

##### Type material.

**HOLOTYPE.** Male: Tanzania, Mkomazi Game Reserve, Kisima Plot, 16 April–2 May 1996, 4°06.06'S, 38°05.58'E, S. van Noort, Malaise Trap, *Acacia*/*Commiphora* bushland, SAM–HYM–P016166 (SAMC). **PARATYPE:** 1 male: Senegal, Parc nat. Niokolo Koba, 24/12/80, B. Sigwalt leg. 2643 (MNHN).

##### Diagnosis.

Female unknown. Male: entirely bright yellow to yellowish–testaceous, flagellum brown with a pale median ring; head densely and shallowly punctate but vertex and temple smooth and polished; clypeus transverse, with very weak lateral protuberances and one strong truncate submedian tooth on ventral margin; hypostomal carina joining occipital carina at mandibular base; mesosoma laterally densely and coarsely punctate, mesonotum polished and sparsely punctate, propodeum transversely punctate reticulate; propodeal carination reduced to incomplete apical transverse carina; metasoma densely and coarsely punctate; gastrocoelus and thyridium indistinct. HdWi 1.3; HfWi 1.2; Ci 2.0; Mi 0.5; IOi 0.9; OOi 1.5; Fli_1_ 3.0; Fli_15_ 1.3; Fli_33_ 2.2; OTi NA.

##### Description.

MALE (2 specimens). B 6.8–7.5; A 4.7–5.0; F 4.2–4.7 (Holotype B 7.5; A 5.0; F 4.7).

*Color*. Bright yellow overall, somewhat darker testaceous dorsally, with frons and inter–ocellar area black; flagellum dark brown with flagellomeres 12–16 pale yellow; wings hyaline, venation yellowish.

*Head*. Frons, face and clypeus densely and shallowly punctate; clypeus strongly transverse, its ventral margin sinuate with paired weak lateral protuberances and one strong and truncate submedian tooth; face hardly bulging medially; malar line moderate without distinct sculpture; vertex and temple smooth, polished; ocellar triangle barely wider than long; temple long and evenly rounded, head obviously swollen behind eyes; hypostomal carina joining occipital carina at mandibular base; antenna long and slender with 34–39 flagellomeres.

*Mesosoma*. Pronotum densely punctate with a large dorsal smooth area; mesopleuron densely and coarsely punctate, speculum smooth; metapleuron half similarly punctate to almost totally smooth; mesoscutum polished, deeply and moderately densely punctate grading toward apex to quite smooth; scutellum smooth to sparsely punctate; propodeum transversely punctate reticulate, mid–longitudinally concave; carination reduced to an incomplete apical transverse carina. *Wings*. Hind wing with distal abscissa of Cu1 joining 1/Cu&cu–a below its middle.

*Metasoma*. Metasoma densely setose; all tergites densely and coarsely punctate, punctation tending to be smoother toward apex; tergite 1 moderately slender, regularly widened to apex; gastrocoelus and thyridium indistinct.

FEMALE. Unknown.

##### Etymology.

Named after the holotype locality. Noun in apposition.

##### Distribution.

Senegal, Tanzania.

##### Comment.

Two males of this species have been collected. Surprisingly, the two collection sites are located at the two lateral extremities of the African continent, both specimens showing only slight differences in the density of punctation and the length of antennae. However, both localities fall within the arid Sahel belt that extends from Senegal in the west to Ethiopia and Somalia in the east, paralleling the southern edge of the Sahara desert. From the horn of Africa this arid habitat extends south down the eastern side of Africa through Kenya to northern Tanzania where Mkomazi Game Reseve is situtated. It must then be presumed that this species is far more widespread than indicated by the known distribution. We predict that the species will likely occur throughout the Sahel zone.

**Figure 27.**
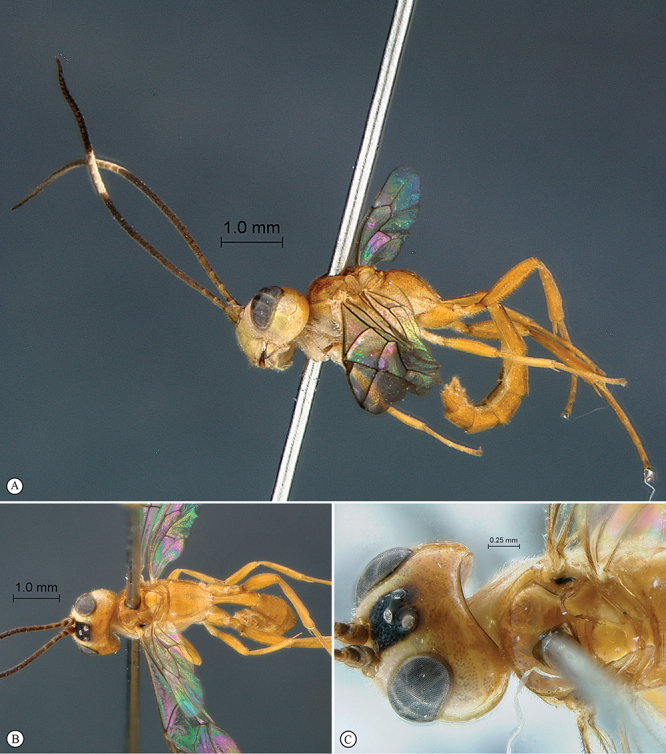
*Heterischnus mkomazi* Holotype male. **A** habitus lateral view **B** habitus dorsal view **C** head, anterior mesosoma, dorsal view.

**Figure 28.**
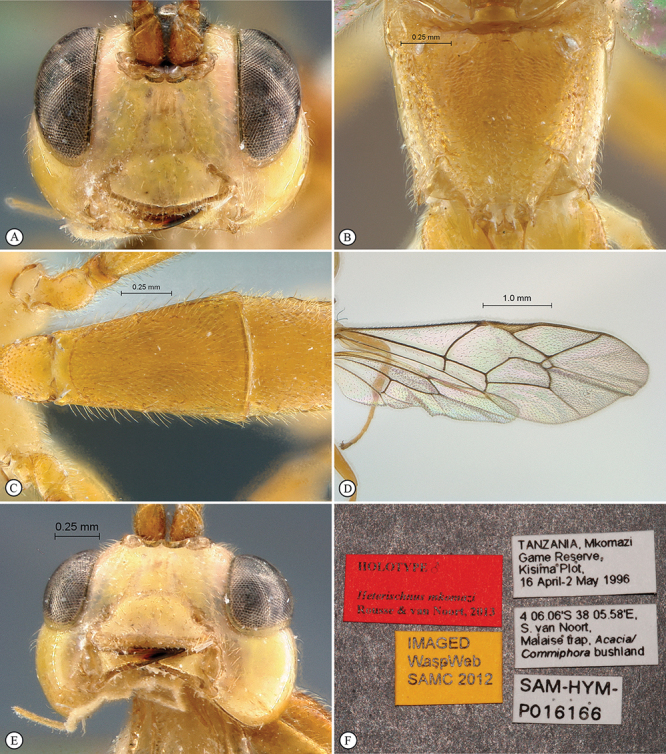
*Heterischnus mkomazi* Holotype male. **A** head, anterior view **B** propodeum dorsal view **C** tergites 1–3, dorsal view **D** wings **E** head, antero–ventral view **F** data labels.

#### Heterischnus olsoufieffi

(Heinrich, 1936)

http://species-id.net/wiki/Heterischnus_olsoufieffi

Figs 29 –30

Aethiopischnus olsoufieffi Heinrich, 1936

##### Material examined.

**HOLOTYPE.** Female: Vatondrangy, Madagascar, XII.29, leg. A. Seyrig (ZMPA). **PARATYPES.** 1 female: Fianar. Madagascar, xi.29 leg. A. Seyrig (ZMPA); 1 male: Madagaskar, Périnet, 100m, 1932.xi, leg. A. Seyrig (ZMPA).

##### Diagnosis.

Head black but mandible, scape and pedicel white; flagellum brown with a pale median ring; remainder of body reddish but dorsal margin of pronotum pale yellow, propleuron and tergites 7 and following black; face puncto–striate, clypeus moderately punctate, frons and vertex polished with fine and very sparse punctures; clypeus bluntly pointed apico–laterally; hypostomal carina joining occipital carina at mandibular base; antenna with 31 flagellomeres; mesosoma moderately punctate but most of pronotum, speculum and scutellum smooth, and antero–dorsal corner of mesopleuron, middle of mesoscutum and propodeum strigose; propodeum with apical transverse carina and some trace of lateral longitudinal carina present; metasoma densely and coarsely punctate; gastrocoelus moderate, thyridium hardly distinct; B 6.2–6.4; A 5.6–5.7; F 4.4–4.6 (Holotype 6.4; A 5.7; F 4.6); HdWi 1.6; HfWi 1.2; Ci 2.0; Mi 1.0; IOi 1.2; OOi 2.0; Fli_1_ 5.8; Fli_15_ 1.2; Fli_29_ 1.5; OTi 0. 3. Male: antenna without pale ring, otherwise similar to female; B 7.8; A 6.9; F 5.5.

##### Distribution.

Madagascar (Antananarivo, Toliara and Toamasina province) (Heinrich 1938).

**Figure 29.**
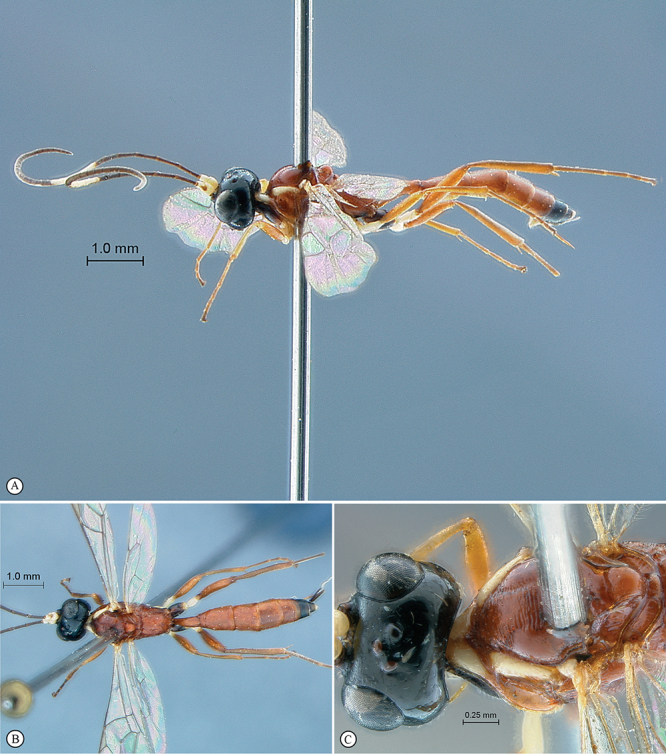
*Heterischnus olsoufieffi* Holotype female. **A** habitus lateral view **B** habitus dorsal view **C** head, mesosoma, dorsal view.

**Figure 30.**
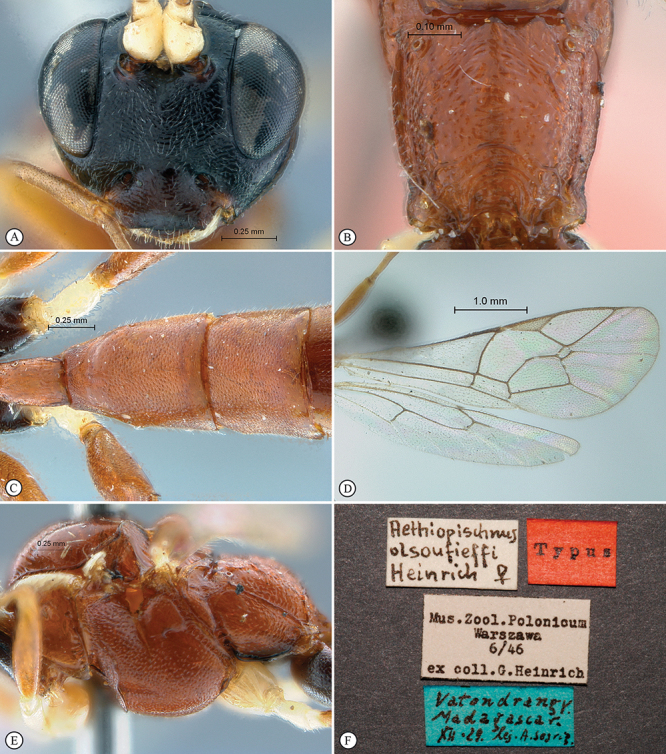
*Heterischnus olsoufieffi* Holotype female. **A** head, anterior view **B** propodeum dorsal view **C** tergites 1–3, dorsal view **D** wings **E** mesosoma, lateral view **F** data labels.

#### Hoplophaeogenes

Heinrich, 1938

http://species-id.net/wiki/Hoplophaeogenes

##### Diagnosis.

Mandible bidentate, upper tooth much longer than lower tooth; clypeus distinctly separated from face, its ventral margin acute and irregularly convex; hypostomal and occipital carinae joining distinctly above mandibular base; flagellum of female enlarged from middle on; pronotum somewhat enlarged ventrally, epomia weak; sternaulus deeply impressed, crenulate, reaching mid–length of mesopleuron; notaulus indistinct; postpectal carina interrupted in front of mid coxae; fore wing with areolet pentagonal, closed; hind wing with distal abscissa of Cu1 present, unpigmented; tarsal claws simple; propodeum fully carinate with strong spine–like apophyses; gastrocoelus and thyridium indistinct.

##### Species richness and distribution.

Genus restricted to Madagascar, with two species.

#### Hoplophaeogenes amoenus

Heinrich, 1938

http://species-id.net/wiki/Hoplophaeogenes_amoenus

Figs 31 –32

##### Material examined.

**SYNTYPES** 1 female: Madagascar, Dieg[o–Suarez], Montagne d’Ambre, 20–26.I.34 leg[. A. Seyrig] (ZMPA). **Other material** (*cf.* comments) 1 male: same label data (ZMPA); 1 male: Madagascar: Fianarantsoa Pr. Ranomafana National Park 21°15.685'S, 47°25.204'E, 927m, Malaise trap 4–21.xi.2005 G. Martin, D.L.J. Quicke, L. P. Holland BMNH (E) 2005–205 (BMNH).

##### Diagnosis.

Head pale yellow with occiput, vertex, and a thin transverse stripe at toruli level black; mesosoma dark red interspersed with black and pale yellow markings; metasoma reddish; antenna black with 29 flagellomeres (broken from flagellomeres 8 and 10 in the lectotype) with a median pale yellow ring; head and body entirely smooth and polished but some rugosity along propodeal carinae; mid–longitudinal bulge of face delimited by moderately deep submedian longitudinal furrow; propodeal carination strong. B: 9.4; A NA; F 6.0; HdWi 1.8; HfWi 1.2; Ci 2.1; Mi 0.7; IOi 1.2; OOi 0.8; Fli_1_ 3.1; Fli_15_ NA; Fli_28_ NA; OTi 0. 3. Male: *cf.* comments.

##### Distribution.

Madagascar (Antananarivo, Antsiranana and Fianarantsoa provinces) (Heinrich 1938).

##### Comments.

This species is very closely related to the following one. Heinrich (1938) considered *Hoplophaeogenes curticornis* to be a distinct species although both species are sympatric and the single known female specimen of *Hoplophaeogenes curticornis* differs from the two known females of *Hoplophaeogenes amoenus* only by its shorter antennae and darker face and metasomal apex. These differences being much slighter than the sexual dimorphism within the species. Heinrich could not attribute with certainty the three *Hoplophaeogenes* males he examined to either of these species. Subsequently, he decided to allocate them arbitrarly to *Hoplophaeogenes amoenus* but did not include them in the type material. Lacking enough supplementary material, we decide here to follow Heinrich’s choice and allocate the male collected at Fianarantsoa to *Hoplophaeogenes amoenus*. Compared to the type female of *Hoplophaeogenes amoenus*, these males differ by the entirely black flagellum, the more extensively black head, and the dark red metasoma. The only male with complete antennae (BMNH) has 26 flagellomeres.

**Figure 31.**
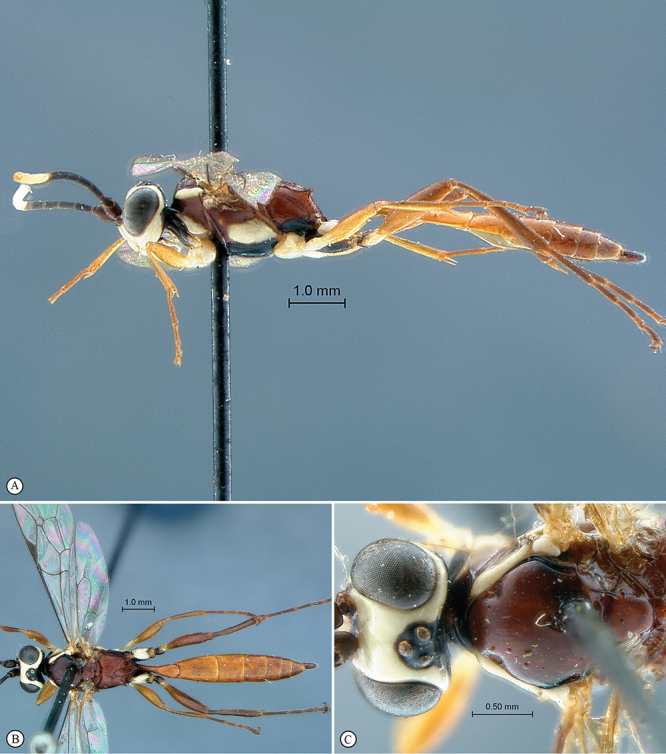
*Hoplophaeogenes amoenus* Holotype female. **A** habitus lateral view **B** habitus dorsal view **C** head, mesosoma, dorsal view.

**Figure 32.**
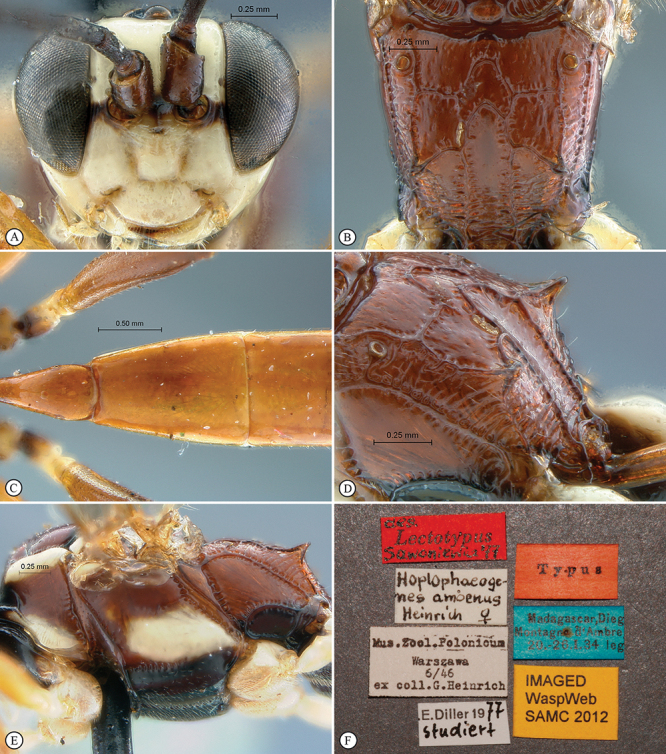
*Hoplophaeogenes amoenus* Holotype female. **A** head, anterior view **B** propodeum dorsal view **C** tergites 1–3, dorsal view **D** propodeum dorso–lateral view **E** mesosoma, lateral view **F** data labels.

#### Hoplophaeogenes curticornis

Heinrich, 1938

http://species-id.net/wiki/Hoplophaeogenes_curticornis

Figs 33 –34

##### Material examined.

**SYNTYPE** 1 female: Madagascar, Dieg[o–Suarez], Montagne d’Ambre, 20–26.I.34 leg[. A. Seyrig] (ZMPA).

##### Diagnosis.

Head pale yellow with occiput, genae and vertex black, and with face and facial orbits dark brown to black; mesosoma dark red interspersed with black and pale yellow markings; metasoma reddish with tergite 4 and following black; antenna with 23 flagellomeres basally testaceous, medially pale yellow, and apically black; otherwise entirely similar to *Hoplophaeogenes amoenus*. B 3.8; F 4.3; HdWi 2.0; HfWi 1.2; Ci 2.2; Mi 0.8; IOi 1.1; OOi 0.9; Fli_1_ 2.5; Fli_15_ 0.8; Fli_22_ 0.7; OTi 0. 3. Male: unknown with certainty (*cf.* *Hoplophaeogenes amoenus* comments).

##### Distribution.

Madagascar (Antsiranana province) (Heinrich 1938).

**Figure 33.**
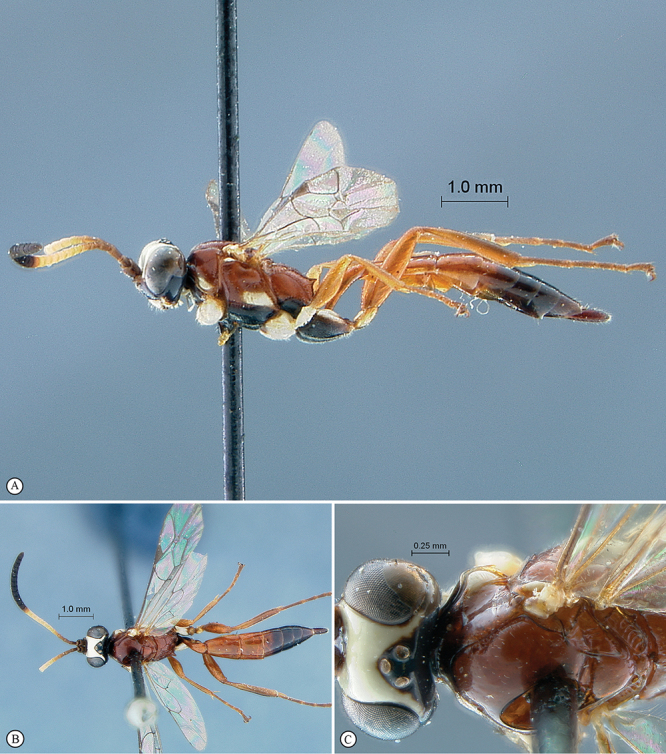
*Hoplophaeogenes curticornis* Holotype female. **A** habitus lateral view **B** habitus dorsal view **C** head, mesosoma, dorsal view.

**Figure 34.**
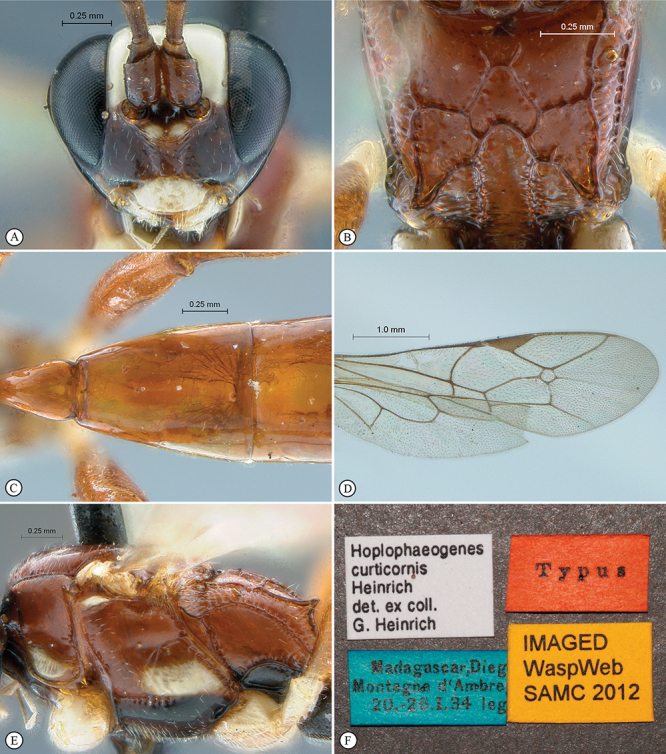
*Hoplophaeogenes curticornis* Holotype female. **A** head, anterior view **B** propodeum dorsal view **C** tergites 1–3, dorsal view **D** wings **E** mesosoma, lateral view **F** data labels.

#### Kibalus

Rousse, van Noort & Diller gen. n.

http://zoobank.org/1198C0DD-EB0E-42F0-A875-2C8018351791

http://species-id.net/wiki/Kibalus

##### Diagnosis.

Head hemispherical, coarsely sculptured; mandible bidentate, upper tooth much longer than lower tooth; mesoscutum steeply elevated above pronotum; postpectal carina complete and strong; propodeum elongate, regularly rounded without differentiated horizontal anterior part in profile view; propodeal carination weak, more or less complete; fore wing with areolet closed; hind wing with distal abscissa of Cu1 absent; gastrocoelus long and shallow, thyridium indistinct; ovipositor very shortly projecting beyond metasomal apex.

##### Description.

*Head*. Hemispherical and coarsely sculptured; temple long and distinctly swollen behind eyes; vertex long and slightly convex behind ocelli; occipital carina complete; face subquadrate; clypeus separated from lower face by a deep groove, its ventral margin acute, rounded and finely serrate with blunt lateral protuberance; tentorial pit deep; mandible bidentate with upper tooth much longer than lower tooth, triangular and regularly narrowed to apex; malar line short to very short; palpi elongate; occipital and hypostomal carinae joined at mandibular base; flagellum long and slender, of female moderately flattened beyond middle, flagellum of male without tyloids.

*Mesosoma*. Slightly elongate, coarsely sculptured; epomia present and strong; mesoscutum strongly elevated above pronotum, regularly rounded in profile view; notaulus weak to absent; scutellum moderately convex, incompletely carinate laterally; sternaulus hardly discernible; epicnemial carina strongly raised ventrally and angled forward between fore coxae; postpectal carina ventrally complete and strong; propodeum moderately long, regularly rounded in profile view, without differentiated horizontal anterior part; carination more or less reduced, spiracle quite round. *Wings*. Fore wing with areolet pentagonal, closed, 3Rs–m non–tubular and faintly pigmented; areolet receiving 2m–cu at its middle; cu–a opposite Rs&M; hind wing with distal abscissa of Cu1 absent, 1/Cu&cu–a reclivous and slightly concave. *Legs*. Hind tibia irregularly shaped, its basal third more or less abruptly constricted; tarsal claws simple.

*Metasoma*. Tergite 1 slender, spiracle near apex, its basal section slightly higher than wide, regularly widened from middle to apex, polished; tergite 2 with gastrocoelus long and shallow, thyridium near mid–length of tergite, transverse and wide; hypopygium hiding base of ovipositor sheath; ovipositor shortly projecting beyond metasomal apex.

##### Etymology.

Named after the Kibale National Park where all specimens have been collected.

##### Distribution records.

Uganda.

##### Discussion.

The general habitus of *Kibalus* gen. n., the hemispherical head with long vertex, the junction of the hypostomal and occipital carinaeat the mandibular base and the complete postpectal carina suggest that it is related to *Lusius* Tosquinet, 1903, from which it differs mainly by the bidentate mandible, the closed areolet and the absence of elongate male genitalia.

##### Genotype.

*Kibalus toro* sp. n.

#### Kibalus mubfs

Rousse & van Noort sp. n.

http://zoobank.org/2C220CF9-7460-4436-8453-E97CD8800FBC

http://species-id.net/wiki/Kibalus_mubfs

Figs 35 –36

##### Type material.

Holotype male: Uganda, Kibale National Park, Kanyawara, Makerere University Biological Field Station, 1523m, 0°33.84'N, 30°21.70'E, 9. viii. 2008, S. van Noort, UG08–KF8–S02, sweep, primary mid–altitude rainforest. SAM–HYM–P044120 (SAMC).

##### Diagnosis.

Female unknown. Male: head and mesosoma mainly reddish yellow, propodeum slightly infuscate dorsally, metasoma brownish with apices of tergites 2–7 yellow; body densely and deeply punctate with propodeum rugulose–reticulate, tergite 1 smooth, following tergites alutaceous; notaulus moderate; propodeum mid–posteriorly concave, carination almost complete with area superomedia present but weak, hexagonal, slightly wider than long. HdWi 1.5; HfWi 1.2; Ci 2.1; Mi 0.2; Di 3.8; IOi 0.8; OOi 1.2; Fli_1_ 5.7, Fli_15_ 2.1, Fli_24_ 1.8; OTi NA.

##### Description.

MALE (Holotype). B 4.2; A 3.2; F 2.8.

*Color*. Head, mesosoma and legs yellow, slightly darkenning dorsally to yellowish orange; propodeum medially infuscate; flagellum brown; metasoma brown with thyridia, spiracular areas and apex of all tergites yellow; wings hyaline, venation light brown.

*Head*. Clypeus transverse, almost smooth with isolated punctures; face slightly transverse and produced forwards; face and frons deeply and densely punctate but finely transversely puncto–striate medially; vertex and temple deeply and densely punctate; gena quite smooth; ocellar triangle slightly wider than long; temple strongly and regularly rounded, head distinctly swollen behind eyes; antenna slender with 25 flagellomeres.

*Mesosoma*. Pronotum deeply rugulose but dorsal margin deeply and densely punctate; mesopleuron and metapleuron deeply, densely and coarsely punctate, punctures sometimes confluent into longitudinal rugosities, speculum smooth; epicnemial carina reaching level of postero–ventral corner of pronotum; mesoscutum deeply and densely punctate, punctation rougher medially; scuto–scutellar groove smooth; scutellum similarly punctate; propodeum rugulose–reticulate, mid–longitudinally concave; propodeal carination anteriorly moderate and posteriorly faint, area superomedia defined, hexagonal, receiving costula anteriorly to middle.

*Metasoma*. Tergite 1 smooth, following alutaceous.

FEMALE. Unknown.

##### Etymology.

Named after the acronym of Makerere University Biological Field Station, where the holotype was collected. The field station is affectionately called “Mubfs” by those privileged to have experienced a stay there. Noun in apposition.

##### Distribution.

Uganda.

**Figure 35.**
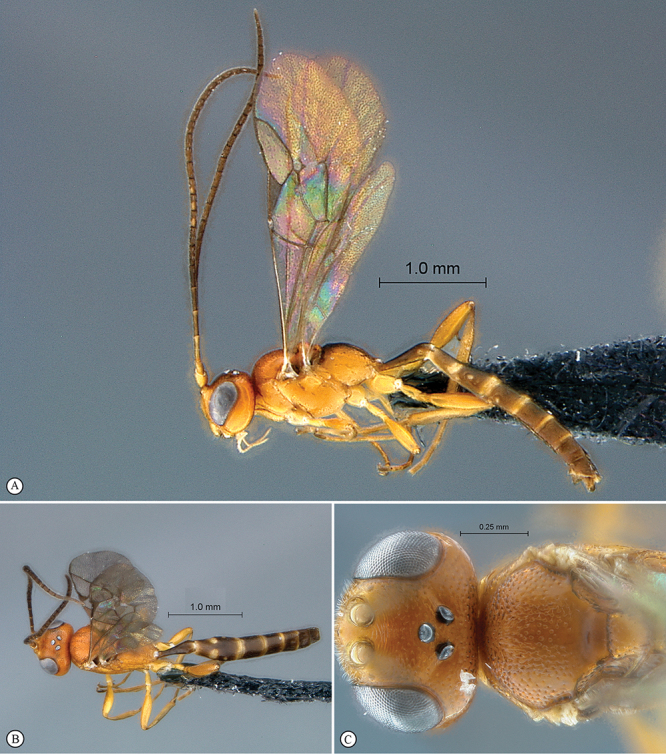
*Kibalus mubfs* Holotype male. **A** habitus lateral view **B** habitus dorsal view **C** head, mesosoma, dorsal view.

**Figure 36.**
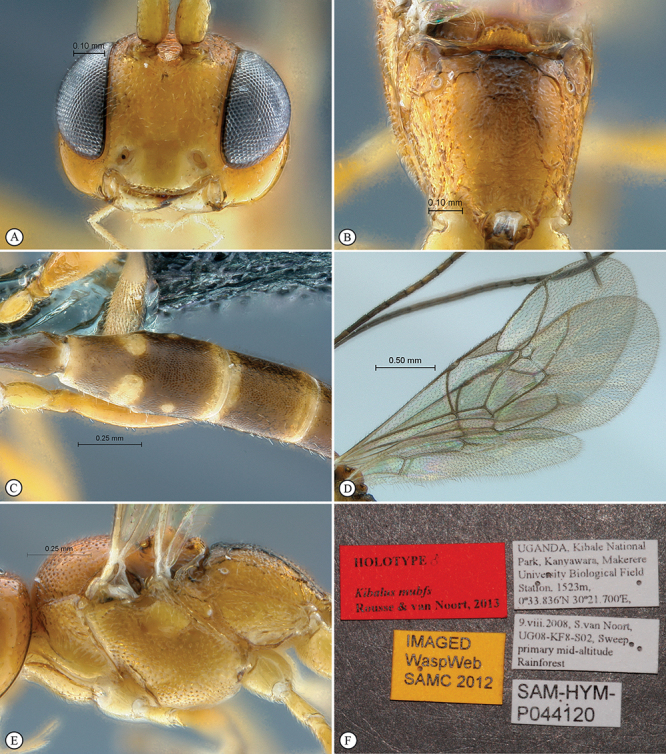
*Kibalus mubfs* Holotype male. **A** head, anterior view **B** propodeum, dorsal view **C** tergites 1–3, dorsal view **D** wings **E** mesosoma, lateral view **F** data labels.

#### Kibalus toro

Rousse, van Noort & Diller sp. n.

http://zoobank.org/2F62341C-E977-4B73-BE78-78E708B4F247

http://species-id.net/wiki/Kibalus_toro

Figs 37 – 39

##### Type material.

**HOLOTYPE.** Female: Uganda, Kibale National Park, Kanyawara, Makerere University Biological Field Station, 1523m, 0°33.836'N, 30°21.700'E, 4–26.viii.2008, S. van Noort, UG08–KF8–Y07, yellow pan trap, primary mid–altitude rainforest SAM–HYM–P044119 (SAMC). **PARATYPES.** 2 females: same data as holotype (SAMC); 1 female: same data as holotype except: 1495m, 0°33.871'N, 30°21.355'E, UG08–KF2–Y03, secondary mid–altitude rainforest, SAM–HYM–P046314 (SAMC); 1 male: same data as holotype except: 1582m, 0°33.823'N, 30°21.490'E, 2–12. viii. 2008, UG08–KF3–M03, Malaise trap, SAM–HYM–P044121 (SAMC); 1 female: Uganda Budongo Forest, 7.ii.1935. F.W. Edwards, B.M. E. Afr. Exp. B. M. 1935–203 (BMNH).

##### Diagnosis.

Head mainly black, mesosoma yellow and dark brown, metasoma dark brown with apices of tergites 2–7 yellow; head and mesosoma almost entirely deeply and densely punctate to punctate–reticulate, tergite 1 smooth, following tergites finely punctate; propodeum without longitudinal carina, transverse carinae present, but incomplete. HdWi 1.4; HfWi 1.1; Ci 1.8; Mi 0.2; Di 4.0; IOi 1.0; OOi 1.0; Fli_1_ 5.9, Fli_15_ 1.0, Fli_28_ 1.8; OTi 0.2.

##### Description.

FEMALE (5 specimens). B 5.1–6.2; A 3.8–4.4; F 3.5–3.9 (Holotype B 5.1; A 3.8; F 3.5).

*Color*. Head black with clypeus somewhat dark testaceous, mandible, palpi, scape and pedicel yellow, flagellum basally yellowish and progressively infuscate, totally fuscous from flagellomere 4, one female specimen with a pale yellow ring on flagellomeres 4–8; mesosoma mostly light testaceous to reddish–testaceous, with black markings of variable extent dorsally, markings absent in one female; legs pale testaceous with tibiae and tarsi sometimes darker; wings hyaline with venation light brown; tergite 1 testaceous to dark brown, following tergites testaceous, more or less extensively dark brown medially, apical margins yellow; thyridium yellow.

*Head*. Shining, almost entirely densely and deeply punctate, punctures somewhat confluent into transverse striations on frons and upper face, punctation much finer and sparser on clypeus and mandible; vertex long and slightly convex behind ocelli; ocellar triangle slightly wider than high; antenna with 25–29 flagellomeres.

*Mesosoma*. Slightly elongate, entirely shining, deeply punctate–reticulate with posterior half of propodeum scaly–reticulate; notaulus absent; propodeum with carination fairly reduced: basal transverse carina nearly absent but two median stubs present, apical transverse carina present though medially obsolete.

*Metasoma*. Tergite 1 polished and smooth; tergite 2 and following finely punctate–reticulate; gastrocoelus within anterior 2/5 of tergite 2, thyridium transverse, about twice as wide as long, inter–thyridiae interval as long as thyridium width; ovipositor straight, sheath densely setose.

MALE (1 specimen). B 5.4; A4.5; F 3.6. Mesosoma dark brown and pronotum yellowish; otherwise similar to female.

##### Etymology.

Named after the Toro Kingdom, the region of western Uganda where this species was collected. Noun in apposition.

##### Distribution.

Uganda.

**Figure 37.**
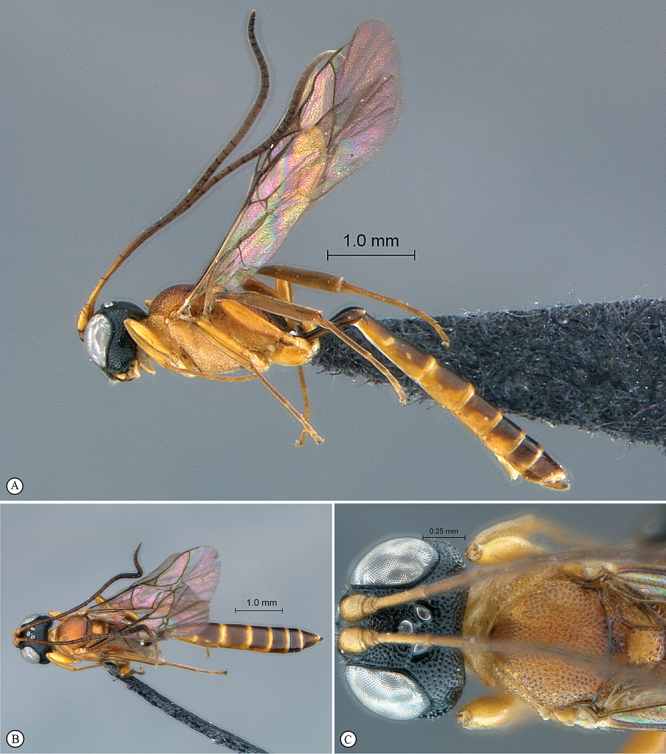
*Kibalus toro* Holotype female. **A** habitus lateral view **B** habitus dorsal view **C** head, mesosoma, dorsal view.

**Figure 38.**
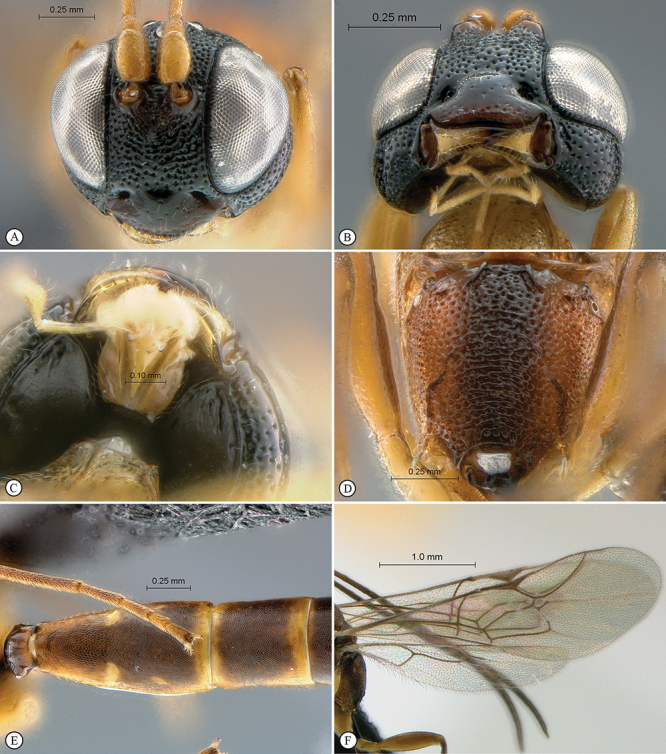
*Kibalus toro* Holotype female. **A** head, anterior view **B** head antero–ventral view, showing mandibles **C** head, ventral view **D** propodeum, dorsal view **E** tergites2–3, dorsal view **F** wings.

**Figure 39.**
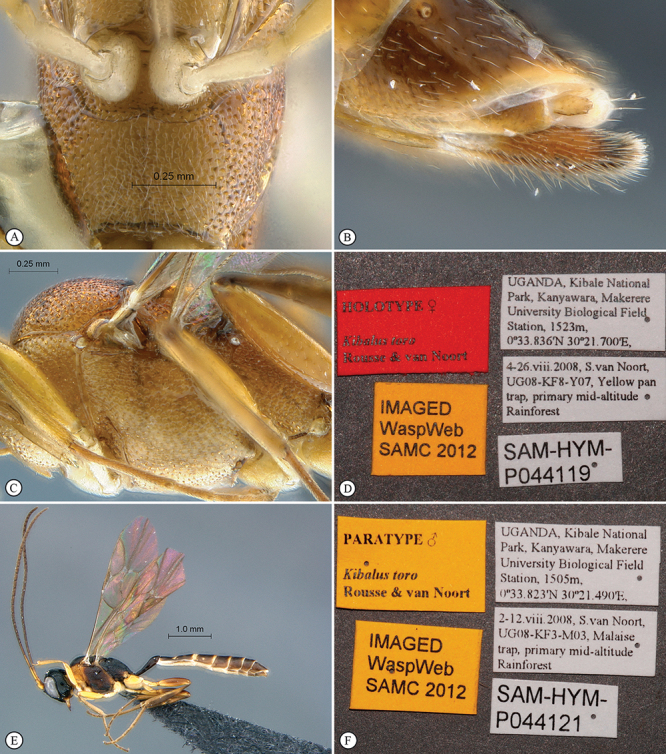
*Kibalus toro* Holotype female (**A–D**), Paratype male (**E–F**). **A** mesopleuron, ventral view **B** tergites 7–8 and ovipositor valves, ventro–lateral view **C** mesosoma lateral view **D** data labels **E** habitus, lateral view **F** data labels.

#### Lusius

Tosquinet, 1903

http://species-id.net/wiki/Lusius

Mesochorischnus Heinrich, 1938

##### Diagnosis.

*Lusius* is also a very distinct genus of Phaeogenini, close to *Heterischnus*. It can be separated from other genera in the tribe by the combination of the following characters: head hemispherical; mandible unidentate and falcate; basal flagellar segments slender and long; vertex long and slightly convex behind ocelli; occipital and hypostomal carinae joining at mandibular base; notaulus complete, ending posteriorly in a median depression; fore wing with areolet open, hind wing with distal abscissa of Cu1 absent; gastrocoelus long with thyridium faint; ovipositor extending beyond apex of metasoma; male with gonoforceps mesochorine–like, *i.e.* expanded into elongate process (Tosquinet 1903, Baltazar 1964; Diller 2006).

##### Species richness and distribution.

The genus is represented by seven species in the Afrotropical, Neotropical and Oriental regions, with one Afrotropical species. We describe here a new species from Uganda.

#### Lusius flummox

Rousse & van Noort sp. n.

http://zoobank.org/91B78FDC-0509-4028-8C0F-79F2F212011B

http://species-id.net/wiki/Lusius_flummox

Figs 40 –41

##### Type material.

**HOLOTYPE.** Female: Uganda, Kibale National Park, Kanyawara, Makerere University Biological Field Station, 1523m, 0°33.836'N, 30°21.700'E, 4–26. viii. 2008, S. van Noort, UG08–KF8–Y07, yellow pan trap, primary mid–altitude Rainforest, SAM–HYM–P044118 (SAMC). **PARATYPE.** 1 female: Mulange, Uganda, Nov. 1922, R. Dummer, SAM–HYM–P007141 (SAMC). **Other material.** 1 female: Sapoba, Nigeria, 3.IX.1962, D.C. Eidt, Malaise trap (CNCI).

##### Diagnosis.

Head and metasoma mostly yellowish, metasoma with dorsal brown maculae; head mostly faintly sculptured but face densely punctate; clypeus very high, strongly pointed apico–laterally; frons with a faint mid–longitudinal carina; antenna very long and slender; mesosoma laterally punctate–reticulate, mesoscutum wrinkled, propodeum reticulate; both transverse carinae of propodeum present; epicnemial carina mid–ventrally highly raised and medially anterior–pointing into a sharp angle; gastrocoelus moderately deep, thyridium wide and oblique. HdWi 1.7; HfWi 1.1; Ci 1.2; Mi 1.0; IOi 1.1; OOi 1.4; Fli_1_ 8.3; Fli_15_ 1.5; Fli_37_ 1.8; OTi 0.6. Male unknown.

##### Description.

FEMALE (2 specimens). B 8.0–8.4; A 6. 5; F 4.7–4.9.

*Color*. Head bright yellow with vertex, occiput, scape and pedicel infuscate, inter–ocellar area and flagellum dark brown (but *cf.* comments); mesosoma bright yellow with mesoscutal lobes and propodeum brown; wings hyaline, venation light brown; legs yellow with tibiae infuscate, hind tibia and all tarsi brown; tergite 1 dark brown, the following brown and apically yellow, thyridium yellow.

*Head*. Face quadrate, densely punctate; clypeus smooth, very high, its apical margin straight, hardly rounded medially and strongly pointed laterally; malar line long with subocular sulcus deep; palpi elongate, maxillary palpus reaching beyond middle of mesosternum; frons hardly sculptured with a faint Y–shaped median carina; vertex sparsely punctate, ocellar triangle wider than long; temple smooth, distinctly swollen behind eyes; antenna very long and slender, slightly enlarged from middle, toruli distinctly protruding, apical truncation of scape and first flagellomeres strongly oblique, flagellum with 38 flagellomeres (paratype with antennae apically broken).

*Mesosoma*. Entirely shining; pleurae densely and irregularly punctate to punctate–reticulate; pronotum with epomia moderate; sternaulus imperceptible; epicnemial carina ventrally highly raised, medially strongly produced anteriorly into a sharp angle; mesoscutum anteriorly transversely wrinkled, wrinkles coarser posteriorly, notaulus deep, median lobe moderately protruding; scutellum weakly convex, carinate to its apical quarter, smooth with some punctures; propodeum coarsely reticulate, basal and apical transverse carinae complete. *Legs*. Hind tibia irregularly shaped, its basal third abruptely constricted; tarsal claws with strong tuft of setae basally.

*Metasoma*. Tergite 1 slender, smooth with some isolated punctures, its apical third distinctly swollen; tergite 2 and following finely and densely reticulate; gastrocoelus moderately deep within anterior third of tergite 2, thyridium wide and oblique; ovipositor straight and moderately long.

MALE. Unknown.

##### Etymology.

Flummox: “be a mystery or bewildering” in reference to the atypical Ichneumoninae habitus of the genus, which originally flummoxed placement of this new species. Noun in apposition.

##### Distribution.

Uganda. Nigeria? (*cf.* comments).

##### Comments.

The CNCI specimen, from Nigeria, was not included in the type material. It indeed exhibits surprising differences: flagellum bi–colored, yellow from base to flagellomere 15, then with remaining flagellomeres black; overall coloration yellow without darker dorsal markings; and median carina of frons absent. The clypeus is, however, typical of *Lusius flummox*. Whether this specimen belongs to *Lusius flummox*, represents a distinct new species, or is an intermediate linking *Lusius flummox* and *Lusius tenuissimus* is currently unclear. We refrain from describing it as a new species until further material is available to enable an informed assessment of this species’ variability.

**Figure 40.**
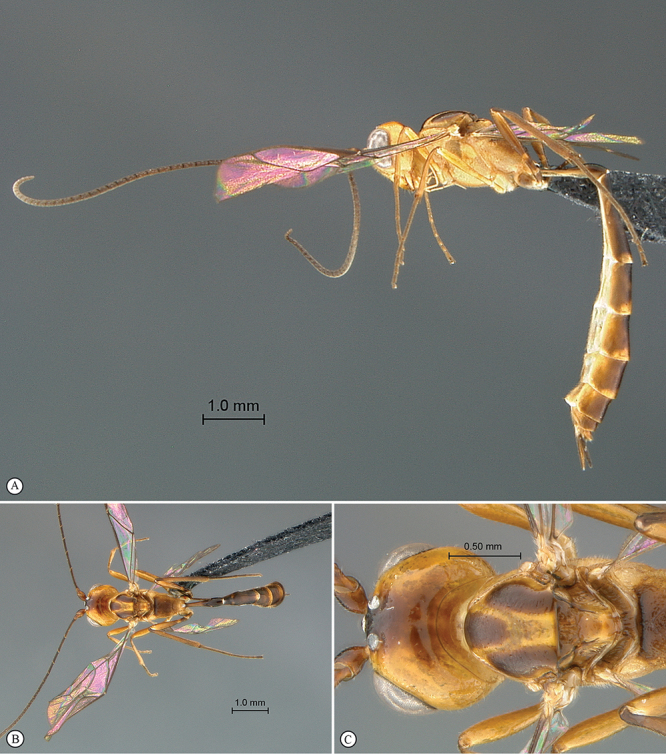
*Lusius flumox* Holotype female. **A** habitus lateral view **B** habitus dorsal view **C** head, mesosoma, dorsal view.

**Figure 41.**
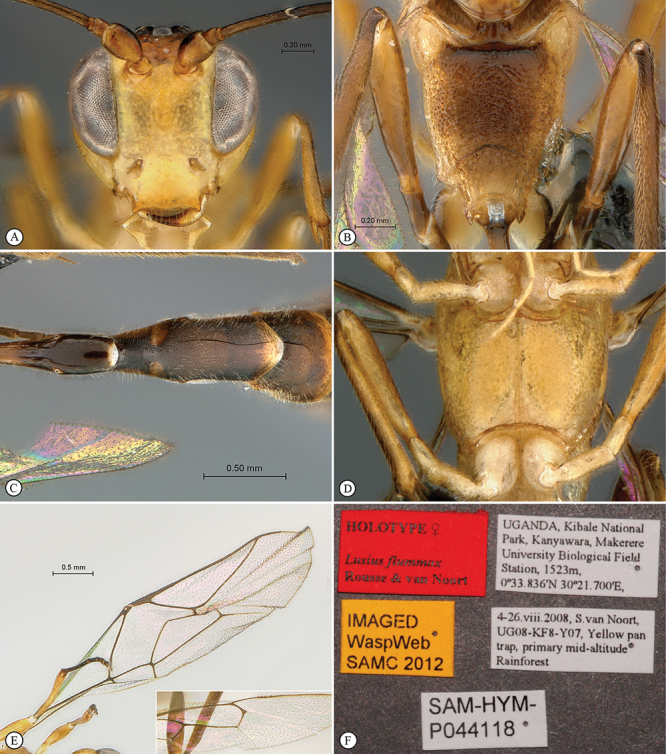
*Lusius flummox* Holotype female. **A** head, anterior view **B** propodeum dorsal view **C** tergites 1–3, dorsal view **D** mesopleuron, ventral view **E** wings **F** data labels.

#### Lusius tenuissimus

(Heinrich, 1938)

http://species-id.net/wiki/Lusius_tenuissimus

Figs 42 –43

##### Material examined.

Democratic Republic of Congo 2 females 3 males: Congo Belge: Kivu Rutshuru 1285m. 11.vii.1935. G.F de Witte: 1083b. (MRAC); Kenya 1 female: KENYA, Ruma National Park, 1250m, 0°39.121'S, 34°19.417'E, 26 July 2008, S. van Noort, UG08–KEN–S09, sweep, savanna woodland, SAM–HYM–P047367 (NMKE); Malawi 1 female: Nyasaland, Mlanje, 5.IX.13, 2, 300 ft, S.A. Neave, 1914–75, Imperial Bureau of Entomology (BMNH); Nigeria 1 female: Ibadan, Nigeria, Dec. 18 1962, D.C. Eidt (CNCI); South Africa 1 female: South Africa, Kwazulu–Natal, Umtamvuna Nature Reserve, 31°03.51'S, 30°10.48'E, 160m, 11–18. xi. 2000, S. van Noort, yellow pan, KW00–Y35, coastal forest / pondoland, coastal plateau sour grassland margin, SAM–HYM–P044125 (SAMC); Zimbabwe 1 male: Rhodesia: Salisbury [Harare] Chishawasha i.1979. A. Watsham (BMNH); 1 female: same data as previous specimen except for: Zimbabwe, xii.1980 (BMNH); 1 male: same data as previous specimen except for: ii.1981 (BMNH).

##### Diagnosis.

Entirely pale to bright yellow, more or less yellowish–orange dorsally; basal half of flagellum testaceous, apical half black with a white ring; antenna long and slender, apical half distinctly enlarged, apical truncation of scape strongly oblique; head mostly smooth but face densely and shallowly punctate; clypeus high, its ventral margin medially protruding; antenna with 32–37 flagellomeres; mesosoma laterally densely, shallowly and coarsely punctate, including speculum, but pronotum shallowly strigose; mesonotum nearly smooth, with notaulus long and crenulate and scuto–scutellar groove longitudinally striate; propodeum coarsely reticulate with apical transverse carina and some remnant of submedian longitudinal carinas present apically; epicnemial carina mid–ventrally highly raised and produced anteriorly into a sharp angle; metasoma finely and deeply reticulate but tergite 1 almost smooth; gastrocoelus large, oblique and shallow; gastrocoelus long, moderately deep, thyridium at basal fifth of tergite 2. B 6.4–8.2; A 4.5–5.4; F 3.8–4.8 (ranges measured on all observed females); HdWi 1.7; HfWi 1.1; Ci 1.7; Mi 1.1; IOi 1.3; OOi 1.3; Fli_1_ 7.4; Fli_15_ 1.3; Fli_35_ 2.0; OTi 0.3 (indices measured on SAMC female). Male: similar to female. B 6.9; A 5.1; F 4.2.

##### Distribution.

Democratic Republic of Congo,Malawi, Nigeria, South Africa, Zimbabwe (new records). Kenya, Tanzania and Madagascar.

**Figure 42.**
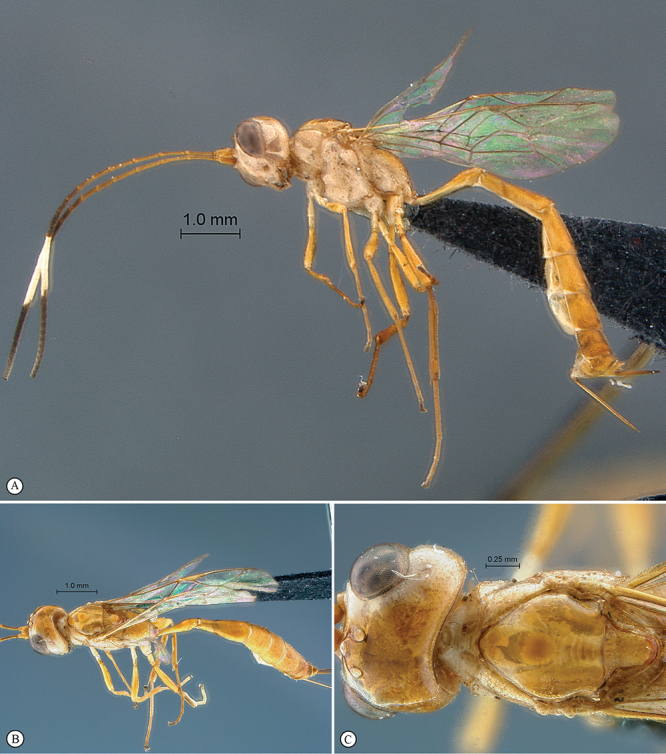
*Lusius tenuissimus* Holotype female. **A** habitus lateral view **B** habitus dorsal view **C** head, mesosoma, dorsal view.

**Figure 43.**
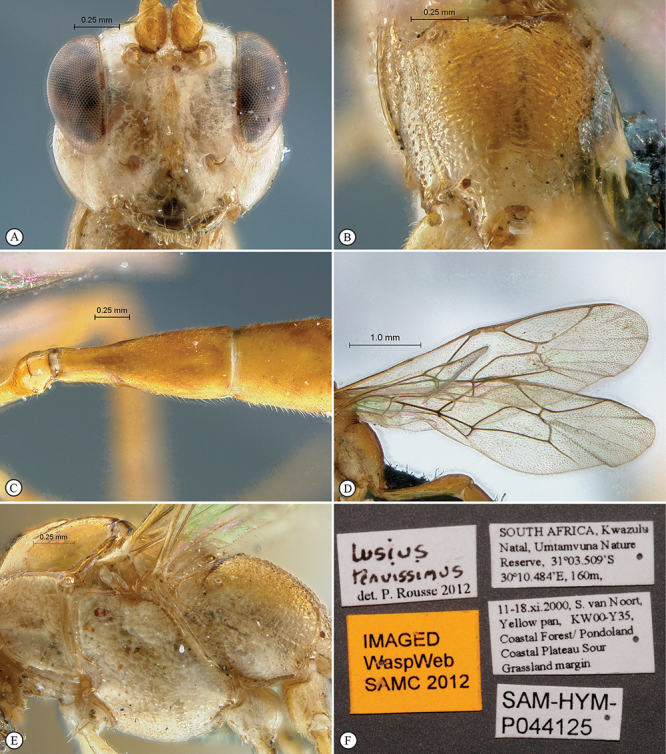
*Lusius tenuissimus* Holotype female. **A** head, anterior view **B** propodeum dorsal view **C** tergites 1–3, dorsal view **D** wings **E** mesosoma, ventral view **F** data labels.

#### Tycherus

Förster, 1869

http://species-id.net/wiki/Tycherus

Glyptichneumon Habermehl, 1917; *Micropa* Schulz, 1906; *Micrope* Förster, 1869; *Proscus* Homgren, 1890.

##### Diagnosis.

Mandible bidentate; clypeus generally smooth with apical margin thin and finely sculptured; face and clypeus conspicuously short; clypeus clearly separated medially from lower face by a distinct groove; occipital carina meeting hypostomal carina before mandibular base; hind coxa simple or at most witha weak ventral spine; fore wing with areolet closed; hind wing with distal abscissa of Cu1 present; gastrocoelus deep, thyridium large (Perkins 1959, Selfa and Diller 1994).

##### Species richness and distribution.

The genus is represented by about one hundred species in the Palaearctic, Neotropical and Oriental regions. The two species described below are the first species reported from the Afrotropical region.

#### Tycherus amatola

Rousse & van Noort sp. n.

http://zoobank.org/8864EA16-BAAB-4B50-9832-2D1E4594CA3E

http://species-id.net/wiki/Tycherus_amatola

Figs 44 –45

##### Type material.

**HOLOTYPE.** Female: South Africa, Hogsback, Amatola Mts, C. P., R. F. Lawrence, Feb. 1933, SAM–HYM–P05530 (SAMC).

##### Diagnosis.

Head mostly yellow, dorsally black; mesosoma black and reddish with pale yellow stripes; metasoma mostly dark reddish to dark brown; head and body mostly punctate to puncto–striate, all metasomal tergites finely punctate; face laterally delimited by two wide oblique sulci; clypeus short, strongly transverse; scutellum moderately punctate; propodeum transversely striate, propodeal carination anteriorly faint; hind coxa simple; thyridium wide and deep. HdWi 1.9. HdWi 1.2; Ci 2.6; Mi 0.5; Di 1.3; IOi 1.0; OOi 1. 0; Fli_1_ 3.2, Fli_15_ 1.0; Fli_25_ 0.8; OTi 0.2. Male unknown.

##### Description.

FEMALE (Holotype). B 4.0; A 2.1; F 2.7.

*Color*. Head yellow with upper clypeal margin, facial furrow, frons and antenna dark testaceous, vertex, occiput and temple black; mesosoma dorsally reddish with lateral lobe of mesoscutum slightly infuscate, laterally black with a yellow longitudinal stripe on lateral face of pronotum, another on mesopleuron, additional yellow markings on middle of mesoscutum and lateral sides of scutellum; wings hyaline, venation light brown; legs testaceous, coxae and trochanters largely interspersed with yellow; metasoma basally dark reddish–brown, progressively lighter toward apex, apical margins of tergites 3–7 yellow.

*Head*. Head slightly transverse in dorsal view; face medially finely wrinkled, laterally delimited by wide, deep, and punctate–rugose sulci; clypeus short, transverse, smooth with some punctures along apical margin, apical margin medially slightly convex; mandible stout, regularly tapered toward apex; malar line with subocular sulcus present as a deep groove; frons and vertex punctate–granulate, ocellar triangle slightly wider than high; temple and occiput closely punctate; temple moderately long, regularly rounded behind eyes; occipital and hypostomal carinae joining distinctly above mandibular base; antenna with 26 flagellomeres.

*Mesosoma*. Pronotum longitudinally strigose with a mid–dorsal smooth area; mesopleuron longitudinally puncto–striate, speculum finely punctate, sternaulus hardly perceptible; metapleuron longitudinally puncto–striate; mesoscutum densely punctate, anteriorly and around lateral lobe finely puncto–striate, notaulus reduced to a moderate impression near anterior margin; scutellum finely and moderately densely punctate, weakly convex, carinate near base only; propodeum transversely striate, carination complete but faint on anterior half, area superomedia as long as wide, heart–shaped, receiving costula at middle. *Wings*. Hind wing with distal abscissa of Cu1 faint, connected to 1/Cu&cu–a, cu–a very short. *Legs*. Densely punctate; hind coxa simple, without ventral tooth.

*Metasoma*. All tergites finely and very densely punctate but gastrocoelus and lateral area of postpetiole coarsely punctate–reticulate, thyridium smooth; gastrocoelus within basal quarter of tergite 2, moderately deep, thyridium deep and wide, about twice as wide as inter–thyridia interval; ovipositor moderately short.

MALE. Unknown.

##### Etymology.

Named after the type locality in Amatola Mountains, meaning “place of many calves” in the Xhosa local language. Noun in apposition.

##### Distribution.

South Africa.

**Figure 44.**
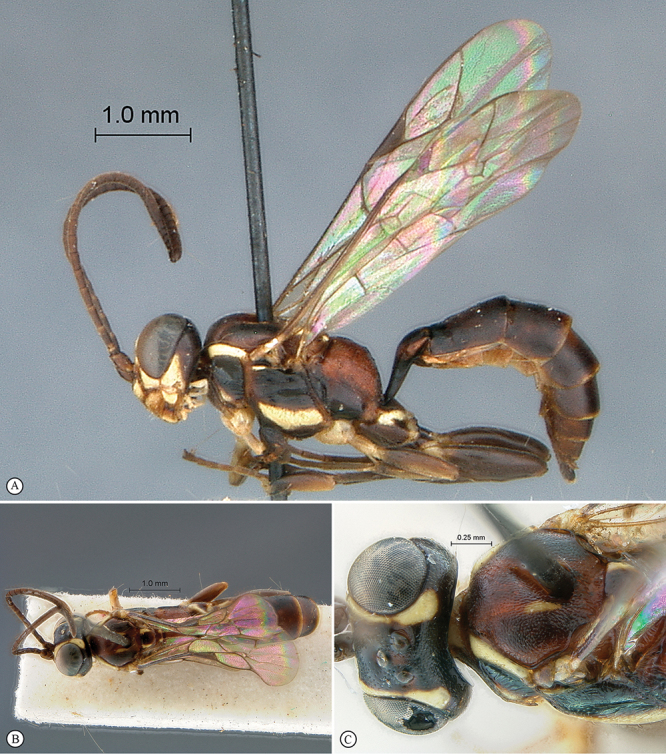
*Tycherus amatola* Holotype female. **A** habitus lateral view **B** habitus dorsal view **C** head, mesosoma, dorsal view.

**Figure 45.**
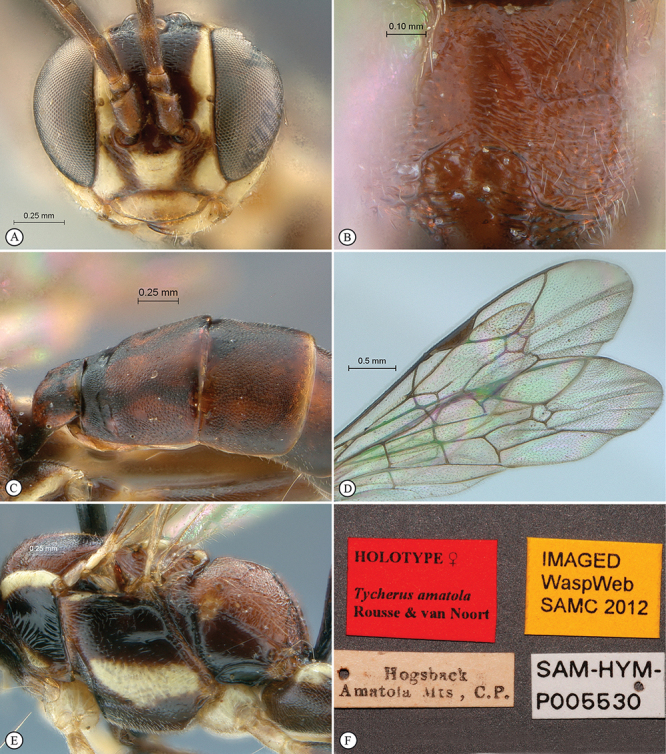
*Tycherus amatola* Holotype female. **A** head, anterior view **B** propodeum dorsal view **C** tergites 1–3, dorsal view **D** wings **E** mesosoma, ventral view **F** data labels.

#### Tycherus nardousberg

Rousse & van Noort sp. n.

http://zoobank.org/A84631EB-F285-467F-BD50-CAA55D38AB18

http://species-id.net/wiki/Tycherus_nardousberg

Figs 46 –47

##### Type material.

**HOLOTYPE** Female: South Africa, Eastern Cape, Asante Sana Game Reserve, 32°14.930'S, 24°56.975'E, 1642m, 6.x.2010–17.i.2011, S. van Noort, Malaise trap, Southern Karoo Riviere *Leucosidea* dominated, ASA09–OUB1–M16, SAM–HYM–P047368 (SAMC). **PARATYPE** 1 female: South Africa, Eastern Cape, Asante Sana Game Reserve, 32°16.762'S, 24°57.309E, 1186m, 28 July–6 Oct 2010, S. van Noort, Yellow Pan, Southern Karoo Riviere, Riverine Woodland, ASA09–WOO1–Y18, SAM–HYM–P047369 (SAMC); 1 female, same label data except for: 29.x.2009–23.ii.2010 ASA09–WOO1–Y03 SAM–HYM–P047370 (SAMC); 1 female, same label data except for: 32°14.990'S, 24°55.962'E, 2183m, 23 Feb–7 April 2010, Malaise trap, Karoo Escarpment Grassland, ASA09–GRA1–M03, SAM–HYM–P047371 (SAMC); 1 female, same label data except for: 32°15.841'S, 24°57.091E, 1354m, 23 Feb–7 April 2010, Camdeboo Escarpment Thicket, ASA09–BUS1–Y07, SAM–HYM–P047372 (SAMC).

##### Diagnosis.

Head black and white; background of body reddish–orange to dark testaceous, mesosoma mid–laterally black with upper margin of pronotum white; antenna short and stout, all flagellomeres but basal ones shorter than wide; head mostly densely and shallowly punctate; mesosoma laterally puncto–striate, dorsally densely punctate; metasomal tergites almost punctate–reticulate; hind coxa simple; gastrocoelus moderate, thyridium wide. HdWi 1.8; HfWi 1.3; Ci2.1; Mi 0.8; Di 3.7; IOi 2.0; OOi 1.3; Fli_1_ 2.0; Fli_15_ 0.6; Fli_26_ 0.8; OTi 0.4. Male unknown.

##### Description.

FEMALE (4 specimens). B 4.6–4.9; A 2.3–2.4; F 2.9–3.1 (Holotype B 4.8; A 2.3; F 3.0).

*Color*. Head black with inner orbits, middle of face, clypeus, mandible, palpi and lower gena whitish; antenna blackish, apical half somewhat lighter testaceous; mesosoma reddish–orange with most of pronotum, dorsal half of mesopleuron, axillary trough and metanotum black, scutellum, post–scutellum and ventral corner of pronotum lighter yellowish–orange, and upper margin of pronotum and tegula whitish; metasoma orange, sometimes fading to yellowish apically; legs testaceous with fore and mid coxae and trochanters and all trochantelli whitish, hind coxa and trochanter mottled with whitish and dark testaceous, all tarsi but last tarsomeres fading to yellowish; wings hyaline, venation brownish–orange.

*Head*. Polished and moderately setose; face medially bulging, closely and shallowly punctate, punctures somewhat confluent into transverse rugosities; clypeus more smoothly punctate–granulate, transverse, its ventral margin truncate and thickened; mandibles moderately slender and long; frons, vertex and temple closely punctate; occipital carina joining hypostomal carina distinctly above mandibular base; ocellar triangle slightly wider than long; antenna with 27–29 flagellomeres, short and stout, flagellomere 5 and following wider than long.

*Mesosoma*. Polished and moderately setose; pronotum, mesopleuron and metapleuron roughly longitudinally puncto–striate, speculum smooth; mesoscutum finely and closely punctate, punctures somewhat confluent into transverse striations, notaulus weak in anterior third of mesoscutum; scutellum densely and shallowly punctate; carinate at base; propodeum coarsely and shallowly punctate, sculpture smoother medially, carination complete, area superomedia heart–shaped, receiving costula a little posterior to middle. *Wings*. Hind wing with distal abscissa of Cu1 faint, connected to 1/Cu&cu–a, cu–a very short. *Legs*. Hind coxa simple, without ventral tooth.

*Metasoma*. Entirely closely punctate, almost punctate–reticulate; gastrocoelus within basal fifth of tergite 2, short and moderately shallow, thyridium moderately deep and slightly wider than inter–thyridia interval.

MALE. Unknown.

##### Etymology.

Named after the type locality. Asante Sana Game Reserve includes aspects of the Nardousberg Mountain. Specimens were collected over an altitudinal range of 1354–2183 m on the south–eastern slopes of the Nardousberg. Noun in apposition.

##### Distribution.

South Africa (Eastern Cape).

**Figure 46.**
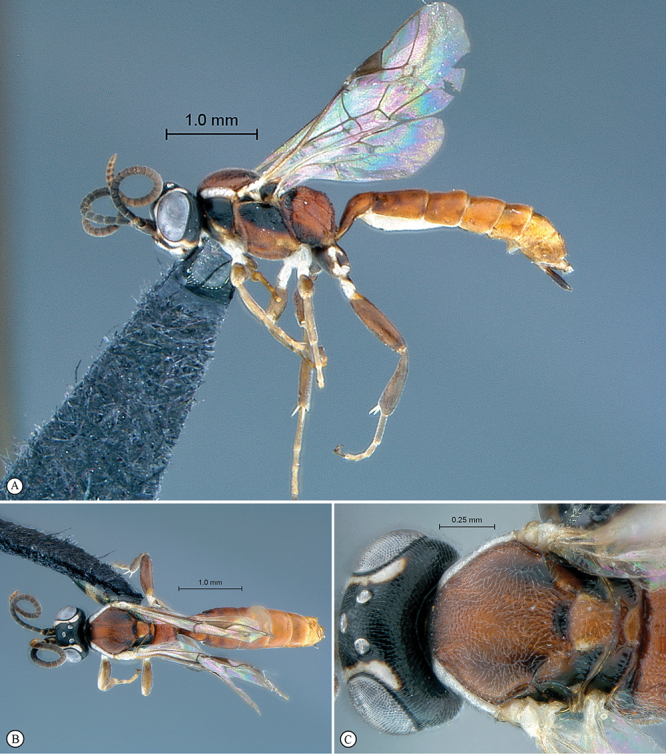
*Tycherus nardousberg* Holotype female. **A** habitus lateral view **B** habitus dorsal view **C** head, mesosoma, dorsal view.

**Figure 47.**
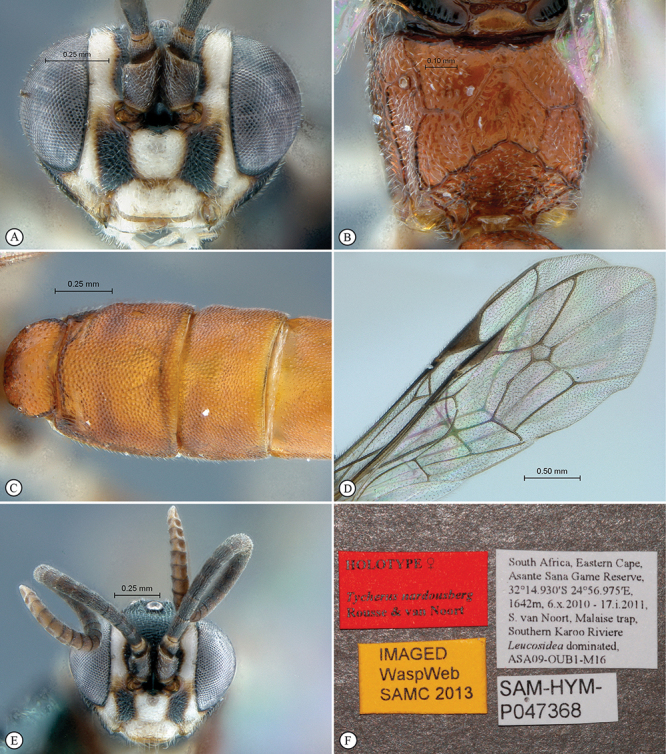
*Tycherus nardousberg* Holotype female. **A** head, anterior view **B** propodeum dorsal view **C** tergites 1–4, dorsal view **D** wings **E** head, antennae, anterior view **F** data labels.

#### Appendix: Doubtful geographical record of *Aethecerus foveolatus* (*cf.* comments)

##### Aethecerus

Wesmael, 1845

http://species-id.net/wiki/Aethecerus

###### Diagnosis.

Genus mainly characterized by the the hypostomal carina strongly raised above mandible base, and the more or less distinctly bell–shaped scape (especially in male). Gena distincly excavate near mandible base in male, slightly excised in female; mandible bidentate, upper tooth much longer than lower tooth; clypeus distinct from face, with a mid–ventral concavity, ventral margin impressed and regularly convex; face quadrate to strongly transverse; face and clypeus very short in female; occipital carina ventrally abruptly curved inwards, joining hypostomal carina above mandible base, hypostomal carina strongly raised above mandible; scape more or less bell–shaped: dorso–apically raised and basally widened, more strongly in male; antenna filiform, not enlarged in either sex, flagellomere 1as long as or longer than flagellomere 2; postpectal carina interrupted in front of mid coxa; fore wing with areolet closed, hind wing with distal abscissa of Cu1 connected to Cu&cu–a; apex of female hind coxa simple or with a short carina; tarsal claws simple; thyridium and gastrocoelus distinct, moderate (Perkins 1959; Valemberg 1988).

###### Species richness and distribution.

*Aethecerus* is presumed to be purely Holarctic, with 27 species reported from North America, Eurasia, Maghreb, Middle East and Japan.

##### Aethecerus foveolatus

Gregor, 1940

http://species-id.net/wiki/Aethecerus_foveolatus

Figs 48 –49

###### Material examined.

1 male: San Thomé [Sao Tome and Principe], J. Ghesquière 1922 (MRAC).

###### Diagnosis

(Valemberg 1988). Female: head and mesosoma black; antenna dark testaceous, basally lighter, sometimes with a weak median pale ring; legs mostly yellow with coxae black; tergites 1–4 reddish, following tergites dark testaceous; head strongly transverse, scape not distinctly enlarged; entire head deeply, regularly and sparsely to moderately densely punctate but frons medially smooth; antenna with 21–22 flagellomeres; mesosoma sparsely to densely punctate; notaulus weak; propodeal carination strong and complete, area superomedia subquadrate to twice longer than wide, area petiolaris concave and transversely striate; tergite 1 apically striate, following tergites rugose punctate. Male: scape stongly swollen basally; antenna slenderer with 24 flagellomeres; tergite 1 black, tergites 2–4 reddish and mid–longitudinally infuscate; otherwise similar to female. B 6.3; A 3.8; F 4.1; HdWi 2.0; HfWi 1.3; Ci 2.7; Mi 0.5; Di 2.2; IOi 1.7; OOi 1.9; Fli_1_3.3; Fli_23_1.4; Fli_23_1.1 (measured on the MRAC male specimen).

###### Distribution.

France, Norway, Poland, Slovakia,Czech Republic, Spain. Sao Tome and Principe ? (*cf.* comments).

###### Comments.

A single male specimen of this species was found in MRAC collections. The island of Sao Tome and Principe being a former Portugese colony, we first suspected that the presence of *Aethecerus foveolatus* there was an accidental introduction. The label on the specimen unfortunately lacks further details about the collection locality, particularly we do not know whether or not it was collected in an anthropogenic habitat. Additionally, there are some other examples in MRAC collections of Hymenoptera labelled as collected in Sao Tome, but which are actually from Madagascar or even Belgium (A. Pauly, *pers. comm*.). The presence, accidental or not, of *Aethecerus foveolatus* in Sao Tome or even in the Afrotropical Region is highly doubtful. We, however, decided to keep the present description and illustrations in the publication because it provides useful and mostly original information for the identification of this widespread European species.

**Figure 48.**
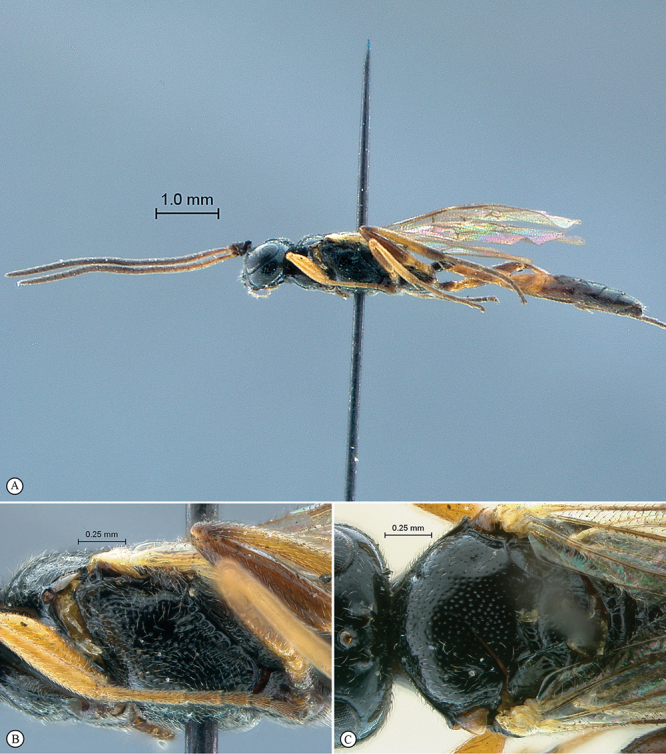
*Aethecerus foveolatus* Gregor, 1940 male. **A** habitus lateral view **B** mesosoma lateral view **C** head, mesosoma, dorsal view.

**Figure 49.**
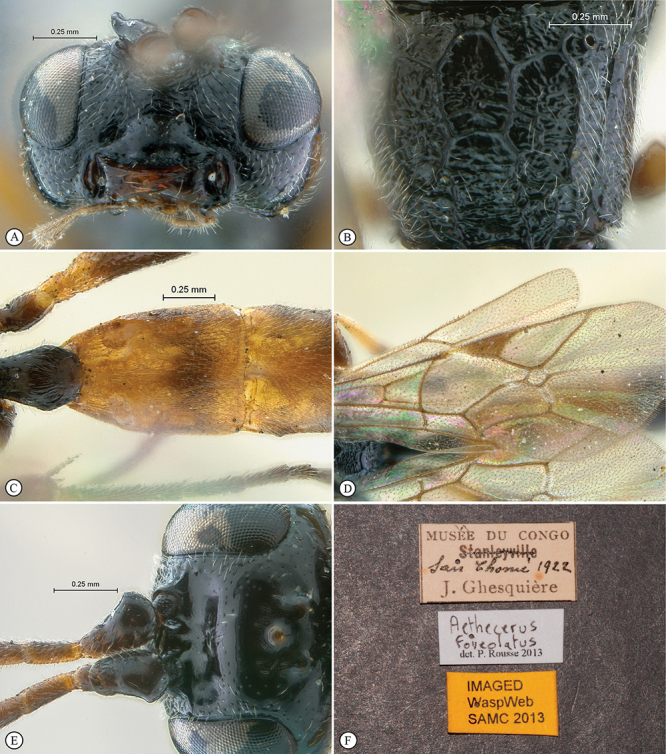
*Aethecerus foveolatus* Gregor, 1940 male. **A** head, antero–ventral view **B** propodeum dorsal view **C** tergites 1–3, dorsal view **D** wings **E** head, antennae, dorsal view **F** data labels.

## Supplementary Material

XML Treatment for Arearia

XML Treatment for Arearia paradoxa

XML Treatment for Arearia oxymoron

XML Treatment for Chauvinia

XML Treatment for Chauvinia nitida

XML Treatment for Chauvinia nyanga

XML Treatment for Chauvinia pelecinoides

XML Treatment for Diadromus (Thyraeella)

XML Treatment for Diadromus collaris

XML Treatment for Dicaelotus

XML Treatment for Dicaelotus asantesana

XML Treatment for Dicaelotus cariniscutis

XML Treatment for Dicaelotus hoerikwaggoensis

XML Treatment for Dicaelotus tablemountainensis

XML Treatment for Heterischnus

XML Treatment for Heterischnus africanus

XML Treatment for Heterischnus krausi

XML Treatment for Heterischnus mfongosi

XML Treatment for Heterischnus mkomazi

XML Treatment for Heterischnus olsoufieffi

XML Treatment for Hoplophaeogenes

XML Treatment for Hoplophaeogenes amoenus

XML Treatment for Hoplophaeogenes curticornis

XML Treatment for Kibalus

XML Treatment for Kibalus mubfs

XML Treatment for Kibalus toro

XML Treatment for Lusius

XML Treatment for Lusius flummox

XML Treatment for Lusius tenuissimus

XML Treatment for Tycherus

XML Treatment for Tycherus amatola

XML Treatment for Tycherus nardousberg

XML Treatment for Aethecerus

XML Treatment for Aethecerus foveolatus
